# Light‐Induced Anion Translocation to Control Helical Folding in an Artificial Communication System

**DOI:** 10.1002/chem.71031

**Published:** 2026-04-24

**Authors:** Indigo M. Bekaert, Suchismita Saha, Julia Villalva, David Villarón, Maxime A. Siegler, Sander J. Wezenberg

**Affiliations:** ^1^ Leiden Institute of Chemistry Leiden University Leiden The Netherlands; ^2^ Department of Chemistry Johns Hopkins University Baltimore Maryland USA

**Keywords:** anion recognition, foldamers, molecular receptors, molecular switches, stiff‐stilbene

## Abstract

The processing of incoming signals is key to many biological functions and involves the transfer of chemical messengers. Emulation of such signaling pathways in artificial molecular systems could enable complex, intelligent behavior. Toward this end, we demonstrate that reversible anion release and uptake by a light‐responsive synthetic receptor can in turn induce helical folding and unfolding of a complementary secondary receptor, of which a change in circular dichroism (CD) absorption serves as output signal. The helical folding process is thus indirectly controlled by light through communication between the two receptors *via* anion translocation. This work will trigger development of complex chemical networks in which signals are transmitted, aiding in the creation of smart materials and molecular computers.

## Graphical abstract

Anion‐induced helical folding of an oligomer is controlled indirectly and reversibly by light through anion uptake/release by a photoresponsive receptor. Thus, a light signal is processed into a chemical signal in a communication‐type three‐component supramolecular system.

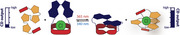

## 1 Introduction

The ability of proteins to decode signals from the environment and transmit them to trigger a cellular response is of vital importance to all living systems. This process—referred to as signal transduction or signal processing—involves the transfer of chemical messengers to other protein targets, to induce conformational changes and control their biological function [1]. One of the most well‐studied examples is visual phototransduction, in which light‐induced double bond isomerization of retinal results in translocation of a G‐protein subunit to a phosphodiesterase, to activate hydrolysis of a second messenger in the transduction cascade. The depletion of this second messenger causes ion channels in the cell membrane to close, which eventually can lead to a neural signal [2, 3]. The imitation of such signaling pathways in artificial, multicomponent (supra)molecular systems could be of great benefit to the development of materials with autonomous and intelligent behavior [4], as well as molecular neuromorphic (computing) devices [5], but is still a fundamental challenge.

Taking inspiration from signaling pathways in biological systems, chemists have created rudimentary communication‐type events in which a chemical messenger is released from a host molecule upon stimulation [6, 7, 8, 9, 10]. This messenger can then interact with another molecule that generates an output signal. Most of these artificial systems rely on the addition of a chemical stimulus, which either displaces the messenger [11, 12, 13, 14, 15, 16, 17] or induces allosteric changes [18, 19]. With respect to light as an input signal, mainly proton release/uptake by photoacids has been used in combination with acid‐base responsive processes [20, 21, 22, 23, 24, 25, 26]. In addition, Berna and McClenaghan exploited photocontrolled release of a neutral barbiturate guest to influence rotaxane ring shuttling [27]. However, despite the importance of protein folding to acquire a functional state, a change in molecular folding as the result of signal processing has not been achieved in artificial systems. Further, where anionic species play important regulatory roles in various biological processes, they have seldom been used as messengers to enable communication between hosts [12, 18, 28].

**SCHEME 1:**
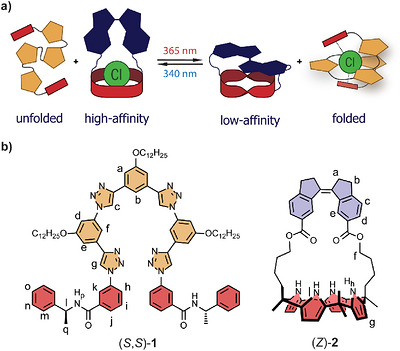
(a) Schematic representation of communication between a light‐responsive receptor and a helical foldamer by chloride translocation and (b) molecular structures of the used aryl‐triazole foldamer (*S*,*S*)‐**1** and stiff‐stilbene strapped calix[4]pyrrole receptor (*Z*)‐**2**.

Previously, our group and others have developed various light‐switchable anion receptors [29, 30, 31, 32, 33, 34, 35, 36, 37, 38], of which a stiff‐stilbene strapped calix[4]pyrrole exhibited the largest difference in chloride binding affinity between isomers [38]. We envisioned its use as a light‐transducing receptor, where the released anion would induce helical folding of a second receptor [39] (Scheme 1a). In such a system, it is crucial that the secondary receptor has a binding affinity in between those of the photoaddressable low‐ and high‐affinity states of the signal‐transducing receptor [i.e. *K*_a_(high) > *K*_a_(foldamer) > *K*_a_(low)] [27]. To obey this criterion, our attention was drawn to aryl‐triazole foldamers originally developed by the groups of Craig [40], Hecht [41], Flood [42], and Jiang [43], which are known to adopt a helical conformation upon binding of halide ions. Interestingly, it has been shown that their helicity is biased by incorporating chiral motifs in their backbone [31, 41, 44]. The resulting circular dichroism (CD) would serve as an ideal output signal for our proposed rudimentary communication‐type system. However, we found the already reported chiral aryl‐triazole foldamers to not be fully compatible with our previously developed photoswitchable calix[4]pyrrole receptor in terms of absorption properties, solubility, and chloride binding affinity. Therefore, a new derivative was designed, with long alkyl instead of oligoether [40, 41, 42] or alkylammonium tails [43], and containing chiral units (that induce a preferred handedness) at both termini as in a previous oligoindole foldamer [45].

In this study, we describe aryl‐triazole foldamer **1** having dodecyl side chains for solubility and a chiral phenylethylamido group at the termini to aid in anion‐hydrogen bonding and bias the helicity (Scheme 1b). Its chloride binding affinity is shown to be right in between that of the *Z*‐ and *E*‐isomer of stiff‐stilbene strapped calix[4]pyrrole **2**, allowing photocontrolled transfer of the anion between the foldamer and the receptor. Hereby, the folding process is indirectly controlled by light. That is, an incoming light signal is translated into chemical messenger release or uptake, which in turn causes helical folding or unfolding, respectively. By having some similarity to the first step in the visual phototransduction cycle, we expect this work to inspire the development of multicomponent (supra)molecular systems able to receive, transmit, and respond to external optical signals, ultimately leading to materials that can process information and exhibit autonomous and intelligent behavior [4, 5].

## 2 Results and Discussion

### 2.1 Foldamer Synthesis and Binding Behavior

**SCHEME 2:**
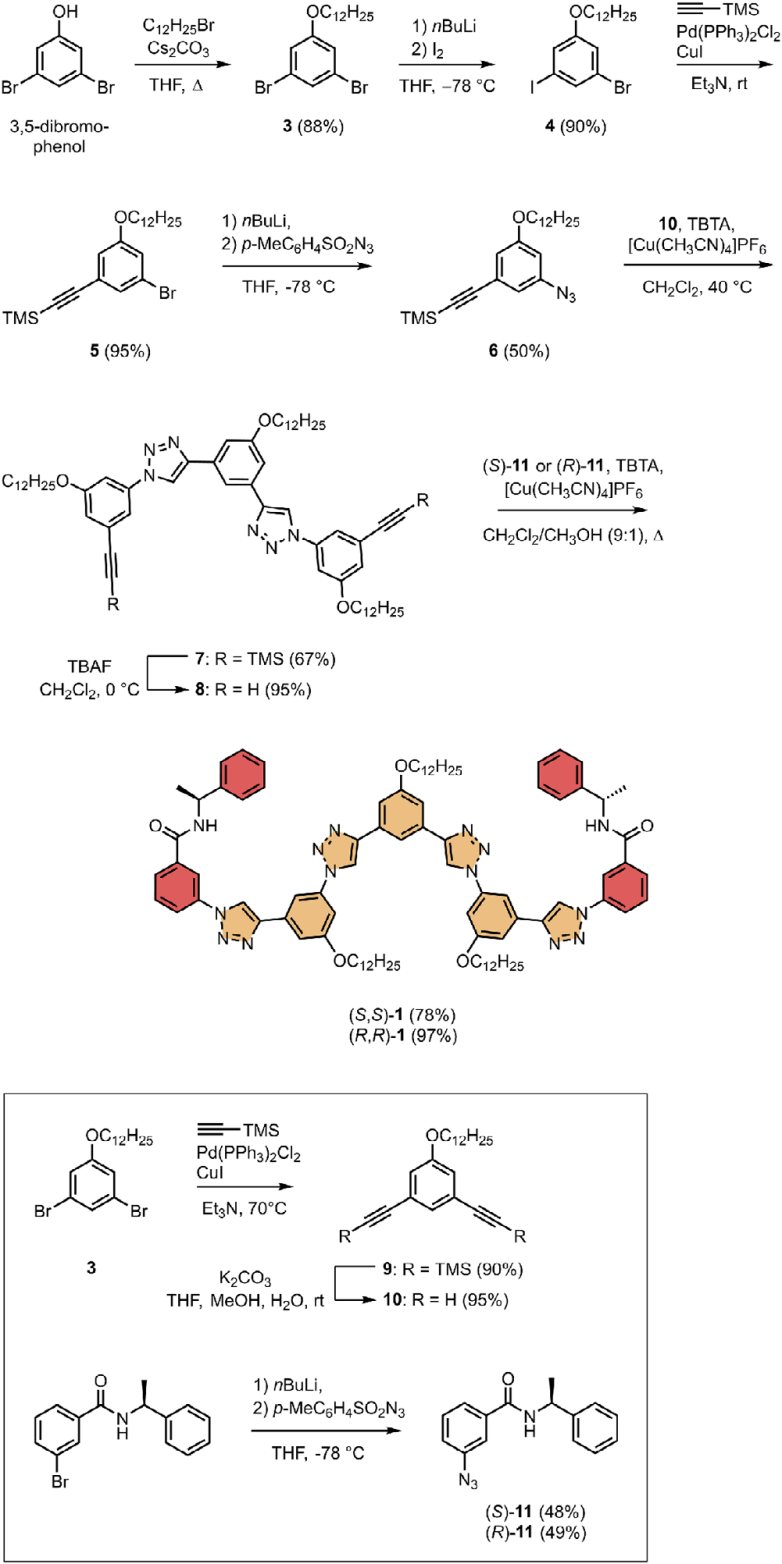
Synthesis of aryl‐triazole foldamer **1**. Only the (*S*,*S*)‐enantiomer of **1** and the (*S*)‐enantiomer of **11** are drawn for convenience.

The synthetic route toward foldamer enantiomers (*S*,*S*)‐**1** and (*R*,*R*)‐**1** is outlined in Scheme 2. First, 3,5‐dibromophenol was functionalized with a dodecyl chain through Williamson ether synthesis to afford compound **3**. Mono‐iodination was achieved by careful addition of one equivalent of *n*‐butyl lithium followed by iodine, to give compound **4**. Subsequent Sonogashira cross‐coupling with trimethylsilylacetylene, under conditions that were sufficiently mild to functionalize solely the iodo‐ and not the bromo‐position, afforded mono‐alkynylated derivative **5**. This intermediate was then subjected to bromo‐lithium exchange, which was followed by treatment with *p*‐toluenesulfonyl azide, to obtain azido‐alkyne building block **6**. Simultaneously, dibromide **3** was doubly alkynylated in a Sonogashira cross‐coupling reaction using trimethylsilylacetylene, to give intermediate **10** after deprotection with potassium carbonate. A copper‐catalyzed azide‐alkyne cycloaddition between this terminal dialkyne and azido‐alkyne building block **6** yielded the trimethylsilyl‐protected foldamer core **7**, which was deprotected using tetrabutylammonium fluoride (TBAF) to give compound **8**. Another cycloaddition reaction using the respective enantiomer of azide‐functionalized amide **11** afforded (*S*,*S*)‐**1** and (*R*,*R*)‐**1** in good overall yields. These chiral amides were prepared from their previously reported aryl bromide precursors [45], through the same lithiation and azidation procedure as used to obtain building block **6**.

**FIGURE 1:**
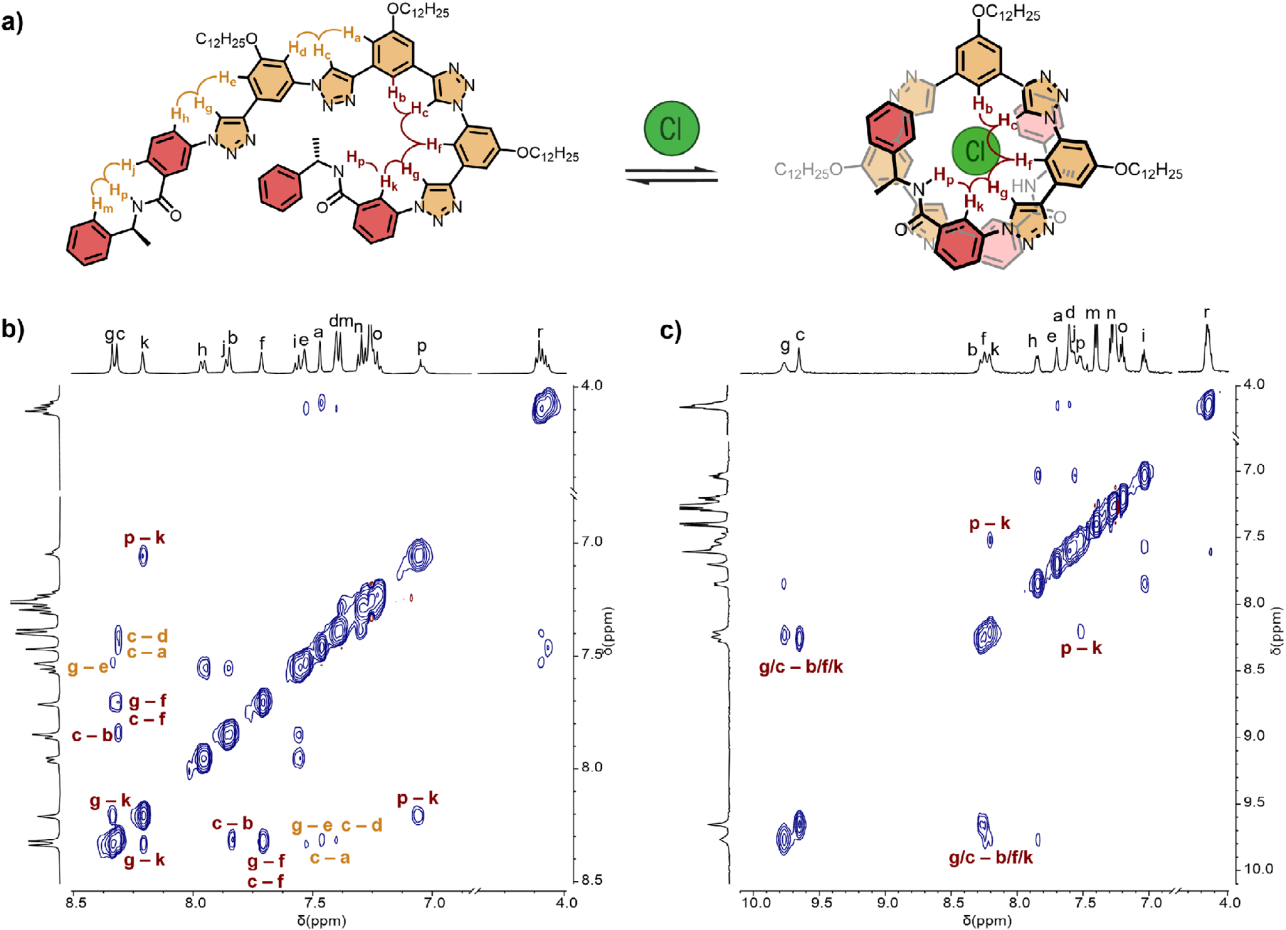
Schematic representation of chloride‐induced helical folding of **1** with possible outer NOE (yellow) and inner NOE (red) interactions indicated (a) and ^1^H‐^1^H NOESY spectrum of (*S*,*S*)‐**1** recorded at 1.0 mM in CDCl_3_ before (b) and after (c) addition of 5 equiv. [Bu_4_N]^+^[Cl]^−^, including the assignment of NOE contacts.

The chloride complexation properties of foldamer **1** were first examined by ^1^H NMR spectroscopy in CDCl_3_ using the (*S*,*S*)‐enantiomer. When [Bu_4_N]^+^[Cl]^−^ was added incrementally to the foldamer solution, downfield displacements of up to 1.5 ppm were seen for the signals of triazole protons H_c_ and H_g_, where also aromatic proton signals H_b_ and H_f_, as well as amide proton signal H_p_, shifted downfield considerably (Figure S23 and Scheme 1 for the lettering assignment). The downfield shifts of the signals for these protons indicate their involvement in hydrogen bonding to the chloride ion in a putative folded state, as depicted in Figure 1a. Conversely, *π*‐*π* stacking induced ring‐current effects caused upfield shifts for the signals that belong to protons H_h_, H_i_, H_j_ H_l_, and H_q_ at the periphery of the foldamer, which was also observed for other aryl‐triazole foldamers upon anion‐induced helical folding [35, 40, 46]. The observed chemical shift changes were fitted to a 1:1 binding model using HypNMR [47], giving an association constant [*K*_a_(**1**, CDCl_3_)] of 1.31 × 10^3^ M^−1^ (Figure S24). To exclude an influence of the countercation on the determined binding affinity, the foldamer was also titrated with [Et_4_N]^+^[Cl]^−^, which resulted in similar ^1^H NMR chemical shift changes (Figure S25). Fitting of the data to a 1:1 binding model now gave an association constant of 1.26 × 10^3^ M^−1^ (Figure S26), showing that the countercation does not significantly influence binding in this case.

Next, 2D ^1^H‐^1^H NOESY experiments were performed to gain additional insight into the preferred conformation before and after chloride binding. In the absence of [Bu_4_N]^+^[Cl]^−^, NOE contacts between all neighboring protons were observed (Figure 1a, b). That is, both triazole protons (H_c_ and H_g_) give an NOE coupling with the protons on either side of the phenyl rings (e.g. H_c_ with both H_a_ and H_b_, H_g_ with both H_e_ and H_f_), indicating unrestricted rotation around all single bonds and the presence of different conformations in solution. Interestingly, in the presence of [Bu_4_N]^+^[Cl]^−^, only (strong) contacts between protons that would point inwards in a helically‐folded state, that is, H_b_–H_c_–H_f_–H_g_–H_k_–H_p_, remained (Figure 1a, c). Similar changes in the NOESY spectrum upon anion addition were observed for structurally related aryl‐triazole foldamers [35, 40, 46], which was related to helical folding. Together with the ^1^H NMR chemical shift changes described above, this 2D NOESY experiment thus supports transitioning from a nonfolded to a helically‐folded state upon chloride addition.

**FIGURE 2:**
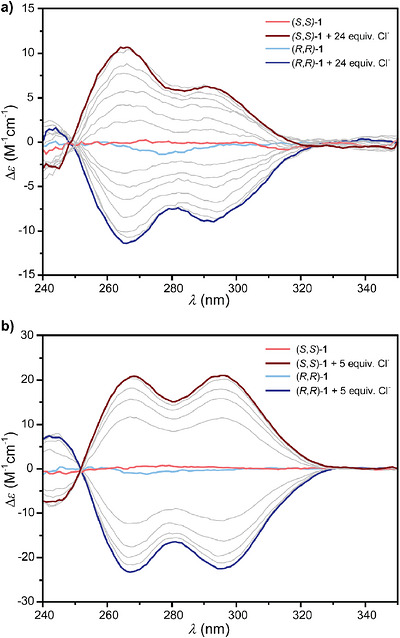
CD spectra of (*S*,*S*)‐**1** and (*R*,*R*)‐**1** before and after addition of [Bu_4_N]^+^[Cl]^−^ in CHCl_3_ (a) and 2.5 vol% CHCl_3_/CH_2_Cl_2_ (b) at 0.10 mM.

To determine a preference for one helical sense over the other in the folded state, CD spectra were recorded. Gratifyingly, when [Bu_4_N]^+^[Cl]^−^ was added to (*S*,*S*)‐**1** in CHCl_3_, a positive CD signal with maxima at *λ* = 268 and 296 nm emerged, whereas the use of (*R*,*R*)‐**1** gave exactly the opposite spectra (Figure 2a). However, at the concentration used for CD studies (0.10 mM; compared to 1.0 mM for the NMR studies) binding saturation was not reached until 24 equiv. of chloride were added, that is, beyond this amount no further spectral changes were noted. Therefore, we repeated the experiment using the less polar CH_2_Cl_2_, in which chloride binding was expected to be stronger. For reasons of solubility, a stock solution of (*S*,*S*)‐**1** or (*R*,*R*)‐**1** in CHCl_3_ was diluted 40× in CH_2_Cl_2_ to result in a 2.5 vol% CHCl_3_/CH_2_Cl_2_ solvent mixture. Indeed, when [Bu_4_N]^+^[Cl]^−^ was added to the foldamer in this solvent mixture, 5 equiv. were sufficient to saturate the sample, while similar but more intense CD absorption was observed than in CHCl_3_, with maxima at *λ* = 269 and 296 nm (Figure 2b). Importantly, in both cases, an isodichroic point was present at *λ* = 252 nm, which alludes to a unimolecular folding process and reveals that the formation of higher‐order species is very unlikely. Furthermore, only minor UV‐vis absorbance changes were observed upon [Bu_4_N]^+^[Cl]^−^ addition and, also here, clear isosbestic points were seen (Figures S27–S30). When the gradual emergence of CD absorption upon chloride addition at *λ* = 266 and 295 nm was fitted to a 1:1 equilibrium using Bindfit [48, 49], association constants of [*K*_a_(**1**, CHCl_3_)] 1.43 × 10^3^ M^−1^ and [*K*_a_(**1**, 2.5 vol% CHCl_3_/CH_2_Cl_2_)] 1.30 × 10^4^ M^−1^ were found (Figures S31–S34). The former value is in excellent agreement with the one determined by ^1^H NMR titration [*K*_a_(**1**, CHCl_3_) = 1.31 × 10^3^ M^−1^, *vide supra*]. The binding strength is thus nearly ten times enhanced by changing solvent from CHCl_3_ to 2.5 vol% CHCl_3_/CH_2_Cl_2_ [50]. Lastly, it should be noted that the observed CD absorption patterns are similar to those reported for other chiral aryl‐triazole foldamers upon anion‐induced folding [41, 43, 51], while in this case the helicity bias is induced by containing the chiral information in the peripheries instead of the central core.

### 2.2 DFT‐Optimization of Foldamer Structure

**FIGURE 3:**
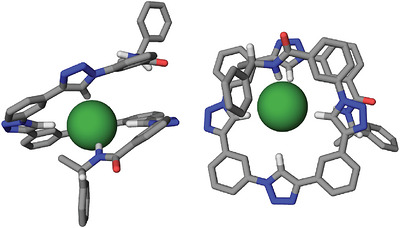
Side‐ and bottom‐view of DFT‐optimized geometry of (*P*)‐(*S*,*S*)‐**1**⊂Cl^−^ with the foldamer shown in stick and the chloride ion in space‐filling representation. Hydrogen atoms that are not involved in chloride binding have been omitted for clarity.

The positive CD signal observed upon chloride addition to (*S*,*S*)‐**1** suggests that the induced helix is preferably right‐handed and *vice versa* for (*R*,*R*)‐**1**_,_ in line with reports for other chiral foldamers [45, 52, 53]. We performed TD‐DFT calculations to support this helical assignment and to gain insight into the relative stabilities of the possible *P*‐ and *M*‐helical chloride complexes of either (*S*,*S*)‐**1** or (*R*,*R*)‐**1**, that is, (*P*)‐(*S*,*S*)‐**1**⊂Cl^−^, (*M*)‐(*S*,*S*)‐**1**⊂Cl^−^, (*P*)‐(*R*,*R*)‐**1**⊂Cl^−^, and (*M*)‐(*R*,*R*)‐**1**⊂Cl^−^. As the former and latter, as well as the middle two complexes, are enantiomeric pairs, we chose to optimize only the *P*‐helical structures (Scheme S1). The DFT‐geometry optimizations were performed at the B3LYP/6‐31+G(d,p) level of theory using an IEFPCM CH_2_Cl_2_ solvation model. To reduce computational costs, the dodecyl chains were removed. The energy‐minimized structure of (*P*)‐(*S*,*S*)‐**1**⊂Cl^−^ in Figure 3 shows the chloride anion bound slightly off‐center through one N(H)_amide_^…^Cl^−^ (distance of 3.56 Å) and four C(H)_triazole_^…^Cl^−^ hydrogen bonds (distance of shorter three average to 3.83 Å, and one longer one of 4.50 Å), which is in line with the large shifts of the signals belonging to these protons in the ^1^H NMR spectrum (Figure S23). The minimization of steric interactions of the terminal phenyl group with the helical aryl‐triazole backbone appears to be the most crucial in steering the handedness. In the case of (*P*)‐(*R*,*R*)‐**1**, a distorted structure with only partial helical folding was found (Figure S50), which was 29.1 kJ mol^−1^ higher in energy (ΔΔ*G*) than the one of (*P*)‐(*S*,*S*)‐**1**⊂Cl^−^. Finally, the TD‐DFT [B3LYP/6‐311++G(d,p), IEFPCM CH_2_Cl_2_] computed ECD spectrum for (*P*)‐(*S*,*S*)‐**1**⊂Cl^−^ had the same positive sign as the experimental CD spectrum observed with (*S*,*S*)‐**1** (Figure S51). This result confirms that that (*S*,*S*)‐**1** preferably adopts a *P*‐helix upon chloride binding.

### 2.3 Photoreceptor Solution and Solid‐State Analysis

We envisioned combining foldamer **1** with our previously developed photoswitchable calix[4]pyrrole receptor **2** to control folding indirectly by light (see Scheme 1). However, this receptor has not yet been characterized in the solvents used to study the foldamer. Therefore, first photoisomerization was studied in CH_2_Cl_2_ by UV‐vis spectroscopy, which showed similar absorbance changes upon irradiation as found before in MeCN and DMSO [38]. That is, exposure of a solution of (*Z*)‐**2** to 365 nm light caused a red shift of the maximum from *λ* = 318 to 324 nm, while the longer wavelength absorption between *λ* = 350–375 nm decreased (Figure S36). These spectral changes are indicative of stiff‐stilbene *Z*→*E* isomerization [54, 55, 56], and could be reversed by 340 nm irradiation to regenerate the *Z*‐isomer. This photoisomerization process was additionally followed in CDCl_3_ by ^1^H NMR spectroscopy, showing *E*/*Z* ratios of 81:19 and 58:42 once the photostationary states (PSS) had been reached by irradiation with 365 and 340 nm light, respectively (Figure S37).

**FIGURE 4:**
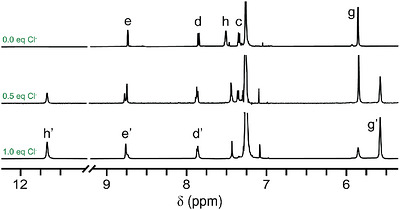
Selected regions in the ^1^H NMR spectrum of (*Z*)‐**2** in CDCl_3_ (0.30 mM), before and after addition of upto 1 equiv. of [Et_4_N]^+^[Cl]^−^. See Scheme 1 for lettering assignment; please note that the signal for H_c’_ overlaps with the residual protiated solvent signal.

Next, the chloride binding properties were investigated by ^1^H NMR titration in CDCl_3_ [57]. While binding in this solvent was initially expected to be stronger than in the more polar MeCN and DMSO [38], addition of excess [Bu_4_N]^+^[Cl]^−^ to a solution of (*Z*)‐**2** did not result in significant spectral changes (Figure S38). This observation reveals that there is virtually no binding in CDCl_3_, as opposed to MeCN, where saturation was already reached in the presence of one equivalent of [Bu_4_N]^+^[Cl]^−^ (Figure S39). A similar solvent effect on anion binding to calix[4]pyrrole was described by Sessler, Schmidtchen, and Gale, who ascribed this to the stronger ion pairing between the anion and the tetrabutylammonium countercation in less polar solvents, which competes with the binding process [58]. Nevertheless, it was shown that the use of a smaller countercation, which fits the bottom side of the calix[4]pyrrole cone, results in strong ion‐pair binding to the host. Indeed, when we used [Et_4_N]^+^[Cl]^−^ instead of [Bu_4_N]^+^[Cl]^−^ in the titration to (*Z*)‐**2**, a new signal set appeared, which could be ascribed to the chloride‐bound species (Figures 4 and S40). For example, new signals were observed for the pyrrolic N–H(H_h’_) protons and the *β*‐pyrrolic protons (H_g’_) at *δ* = 11.67 ppm and 5.58 ppm, respectively, which for the unbound forms are located at *δ* = 7.51 ppm and 5.85 ppm. The observation of distinct signal sets reveals that binding is slow on the NMR timescale. Further, beyond 1 equiv. of chloride, the signals of the unbound species had almost fully disappeared, meaning that binding saturation had been reached. By ^1^H NMR signal integration, the ratio between bound and unbound forms during the titration was estimated, allowing calculation of a binding constant [*K*_a_(**2**, CDCl_3_)] of 2.8 × 10^4^ M^−1^ (Figure S41).

**FIGURE 5:**
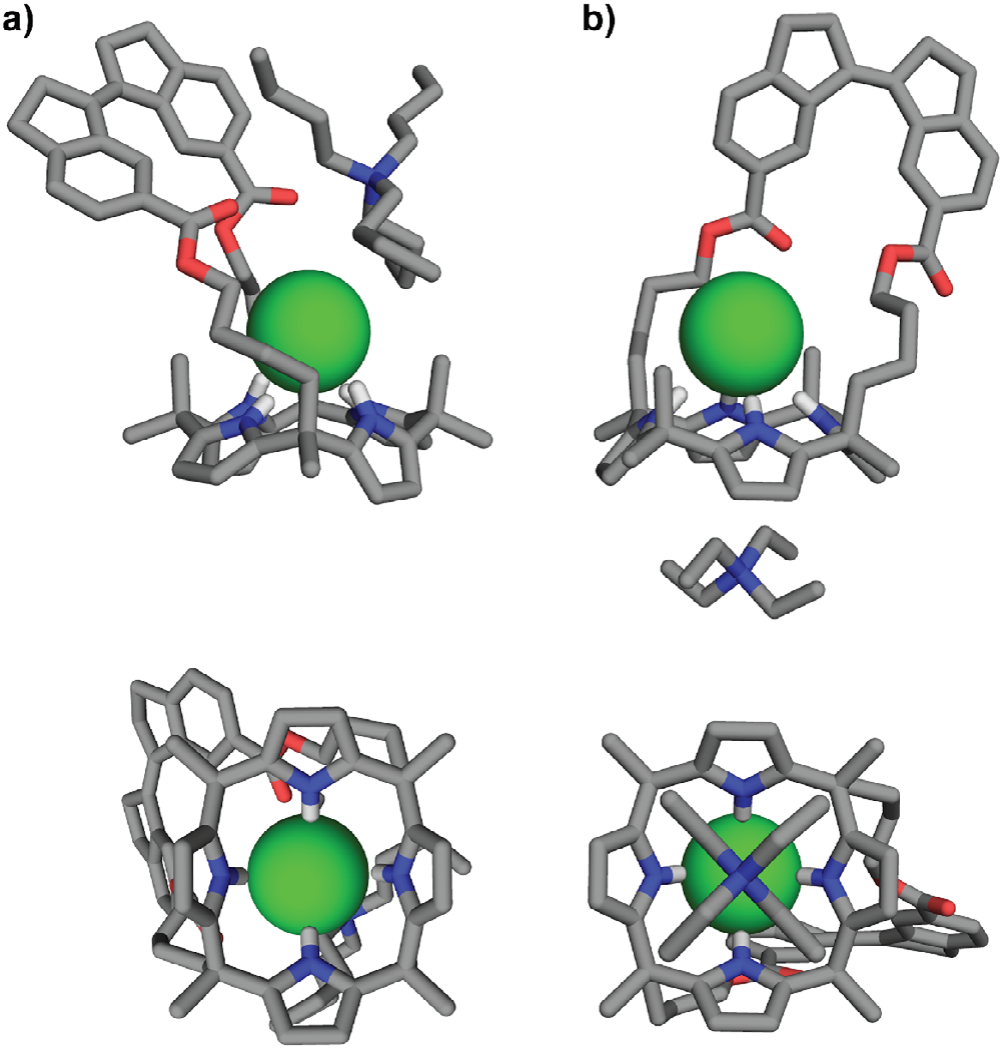
Side‐ and bottom views of (*Z*)‐**2**⊂[NBu_4_]^+^[Cl]^−^ (a) and (*Z*)‐**2**⊂[NEt_4_]^+^[Cl]^−^ (b) as found in the crystal structures, with the receptor and countercation shown in stick and the chloride ion in space‐filling representation.

Pleasingly, single crystals suitable for X‐ray structure determination were obtained for both (*Z*)‐**2**⊂[Bu_4_N]^+^[Cl]^−^ and (*Z*)‐**2**⊂[Et_4_N]^+^[Cl]^−^ by vapor diffusion of *i*Pr_2_O into CHCl_3_ solutions of the receptor combined with either of the salts. The crystal structures, shown in Figure 5, illustrate the noninnocence of the countercation in binding. In both cases, the calix[4]pyrrole unit is found in the cone conformation and the chloride ion is bound *via* four hydrogen bonds with the pyrrolic N‐H atoms [N(H)^…^Cl^−^ distances found in the range 3.2758(16) − 3.3875(15) Å]. However, where the tetrabutylammonium countercation is located in close proximity to the chloride anion and the stiff‐stilbene unit, the tetraethylammonium cation is positioned in the electron‐rich bowl provided by the calix[4]pyrrole cone. In the latter structure, a higher symmetry is seen in this cone, which is reflected in the C_Me_‐C‐C‐N(H) dihedral angles. That is, when the [NEt_4_]^+^[Cl]^−^ ion pair is bound by the calix[4]pyrrole unit, a fully symmetrical cone‐shape is observed with all C_Me_‐C‐C‐N(H) dihedral angles at 47.9(7)°−51.9(6)°, as is illustrated in the bottom view in Figure 5b. For the structure with [Bu_4_N]^+^[Cl]^−^, a more distorted cone conformation is found with C_Me_‐C‐C‐N(H) dihedral angles ranging from 31.9(2)° to 65.0(2)°.

Additionally, the *E*‐isomer (obtained through irradiation of a CDCl_3_ solution of the *Z*‐isomer) was titrated with [Et_4_N]^+^[Cl]^−^. In this case, only minor and gradual shifting of all ^1^H NMR signals was observed (Figure S42). Since now the bound and unbound species were in fast exchange on the NMR timescale, the signal shifts could be fitted to a 1:1 binding model by HypNMR [47], giving an association constant [*K*_a_((*E*)‐**2**, CDCl_3_)] of 18 M^−1^ (Figure S43). Gratifyingly, this constant is much smaller than the one calculated for the *Z*‐isomer [*K*_a_(*Z*)/*K*_a_(*E*) = 1500]. Moreover, the chloride binding constant of foldamer **1** falls right in between, that is, *K*_a_[(*Z*)‐**2**] > *K*_a_(**1**) > *K*_a_[(*E*)‐**2**], which is crucial to achieve effective chloride transfer between the foldamer and the calix[4]pyrrole receptor upon *E*/*Z* photoisomerization of the latter.

### 2.4 Light‐Induced Chloride Translocation

**FIGURE 6:**
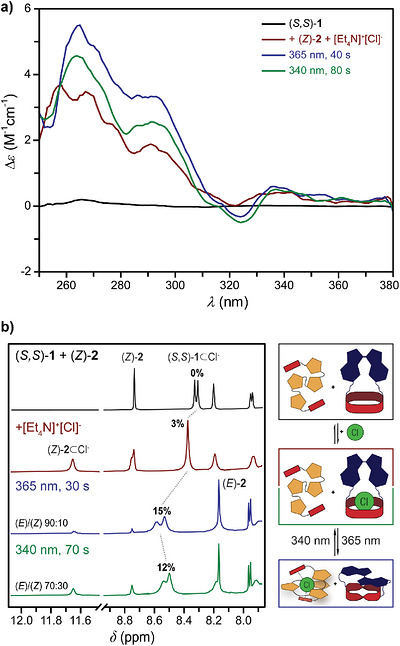
(a) CD spectral changes of a 2.5 vol% CHCl_3_/CH_2_Cl_2_ solution of (*S*,*S*)‐**1** upon addition of (*Z*)‐**2** (both 0.20 mM) and [Et_4_N]^+^[Cl]^−^ (0.10 mM) and after irradiation at 365 and 340 nm until PSS was reached. (b) ^1^H NMR spectral changes of a CDCl_3_ solution of (*S*,*S*)‐**1** and (*Z*)‐**2** (1.0 mM) upon addition of [Et_4_N]^+^[Cl]^−^ (0.5 mM) and after irradiation at 365 and 340 nm until PSS was reached; The percentage of chloride‐bound foldamer is given relative to overall foldamer concentration; The *E*/*Z* ratios of the receptor at the PSS were determined by relative integration of the ^1^H NMR signals.

CD spectroscopy was used to demonstrate that light‐triggered anion uptake and release by receptor **2** controls the conformation of foldamer **1**. Only the anion‐bound foldamer has significant CD absorption, which therefore serves as an ideal output signal of chloride transfer. As shown above, as well as in Figures 6a and S46, foldamer (*S*,*S*)‐**1** in 2.5 vol% CHCl_3_/CH_2_Cl_2_ was virtually CD silent, but when receptor (*Z*)‐**2** (1 equiv.) and [Et_4_N]^+^[Cl]^−^ (0.5. equiv.) were added, a positive CD signal appeared. This CD absorption originates from partial binding of chloride by (*S*,*S*)‐**1** to induce *P*‐helical folding, while the majority of chloride remains bound to (*Z*)‐**2**. Irradiation of the sample with 365 nm light resulted in an increase of the CD absorption, showing that now more of (*S*,*S*)‐**1** was in an anion‐bound folded state as the result of chloride release by **2** upon *Z*→*E* photoisomerization. When subsequently the solution was irradiated with 340 nm, the CD absorption decreased, as chloride was picked up again by the regenerated *Z*‐isomer of receptor **2**. It should be noted that the original CD spectrum was not fully recovered as *E*→*Z* photoconversion is not quantitative (*vide supra*). Importantly, we confirmed that the foldamer is photostable to rule out that the CD absorbance changes would be due to side reactions; No UV‐vis absorption changes were noted upon irradiation of a solution of (*S*,*S*)‐**1** with 365 and 340 nm light (Figure S35).

This process was additionally monitored by ^1^H NMR spectroscopy, which allowed quantification of the amount of chloride bound by the foldamer after each step (Figures 6b and S47). Here, [Et_4_N]^+^[Cl]^−^ (0.5 equiv.) was added to an equimolar solution of (*S*,*S*)‐**1** and (*Z*)‐**2** in CDCl_3_, which led to shifts in the signals of both molecular components, analogous to what was observed in the individual titrations (*vide supra*). That is, for (*Z*)‐**2** the characteristic NH signal (H_h_) for the bound form was found at 11.6 ppm, meaning that most of the chloride present was bound by this receptor. For **1**, only a small downfield shift in the signals of the triazole protons (H_c/g_) was observed, corresponding to minimal chloride binding to the foldamer (3%) [59]. Upon irradiation of the solution with 365 nm light, these signals underwent a large downfield shift, consistent with an increase in the amount of chloride‐bound foldamer (15% of **1** associated). This is the result of chloride release by receptor **2** upon light‐induced *Z*→*E* isomerization. Indeed, the ^1^H NMR signals ascribed to the (*Z*)‐**2**⊂Cl^−^ complex had almost disappeared upon 365 nm irradiation and, by ^1^H NMR signal integration, the *E*/*Z* ratio at this point was determined as 90:10. Subsequent irradiation with 340 nm light led to reappearance of the NH signal (H_h_) for the chloride‐bound (*Z*)‐**2** species and an upfield shift of the triazole proton signals (H_c/g_) of **1**, indicating that the amount of bound foldamer decreased (to 12% of **1** associated). Part of the available chloride that was bound to the foldamer was thus picked up again by the receptor. Here, ^1^H NMR signal integration revealed that 30% of the *Z*‐isomer had been recovered after 340 nm irradiation. These combined CD and ^1^H NMR experiments thus confirm reversible chloride translocation between the light‐responsive receptor and the foldamer under consecutive 365 and 340 nm irradiation.

A speciation analysis was then performed with Hyss software [60], to verify the amount of chloride‐bound species under the conditions used in the ^1^H NMR experiment. The concentrations of all species were calculated at the start of the experiment, and at PSS_365_ and PSS_340_, using the *E*/*Z* ratios as obtained by ^1^H NMR signal integration and the binding constants as determined in CDCl_3_ (Table S3). Satisfyingly, the amount of chloride‐bound foldamer calculated in this simulation, that is, 4%, 21%, and 14% binding saturation at the start, PSS_365_ and PSS_340_, respectively, corresponds well with the experimentally determined values. Further, the total amount of receptor (*E*‐ and *Z*‐isomer) that is bound with chloride changes from 44% to 9% to 24%, respectively, where the *Z*‐isomer always picks up the available chloride and binding to the *E*‐isomer is negligible. This analysis thus corroborates our experimental findings and confirms the transfer of chloride between the receptor and the foldamer.

Lastly, as a control, these CD and ^1^H NMR experiments were repeated under similar conditions, but in the absence of foldamer **1**. In the CD spectrum, no changes were noted upon 365 and 340 nm irradiation, whereas in the UV‐vis spectrum the indicative absorption changes for *Z*→*E*, followed by (partial) *E*→*Z* isomerization were observed (Figure S48). This result confirms that the CD absorption changes in the chloride translocation study described above are indeed due to (un)folding of (*S*,*S*)‐**1**. In addition, the ^1^H NMR experiment shows that photoisomerization of receptor **2** still occurs in the presence of [Et_4_N]^+^[Cl]^−^, and absence of foldamer (Figure S49). Furthermore, the PSS_365_ and PSS_340_ *E*/*Z* ratios were determined as 90:10 and 67:33, similar as in the experiment that included the foldamer. However, in these cases, both PSS mixtures are slightly less enriched in *Z*‐isomer than upon irradiation of the photoreceptor alone (∼10%, *vide supra*). This difference indicates that the anion has a minor effect on the photoisomerization properties, which will be studied in more detail in the future.

## 3 Conclusions

We demonstrated light‐controlled reversible translocation of chloride between our previously developed calix[4]pyrrole receptor [38] and an aryl‐triazole foldamer. This foldamer contains chiral motifs at the peripheries to induce a preferred helicity upon anion binding. Its binding affinity falls right in between that of the *E*‐ and *Z*‐isomer of the light‐responsive calix[4]pyrrole receptor, allowing communication between this receptor and the foldamer using chloride as the messenger. In other words, light‐induced chloride release from the photoreceptor and concomitant uptake by the foldamer induces helical folding, which can be detected by CD spectroscopy. This work is expected to stimulate the implementation of more complex chemical networks in (supra)molecular systems, giving the ability to respond to light by the transfer of chemical messengers. Our next step will be to incorporate catalytic groups into the foldamer as well as to embed the responsive components in bilayer membranes, which would represent a closer mimic to the visual phototransduction process. Ultimately, this will facilitate the development of smart and adaptive materials, as well as neuromorphic devices.

## Conflicts of Interest

The authors declare no conflicts of interest.

## Supporting information



Synthetic procedures and full characterization, ^1^H and ^13^C NMR spectra of new compounds (Figures S1‐S22), ^1^H NMR, UV‐Vis, and CD studies, DFT calculations, and X‐ray analysis (CCDC 2470676 and 2470677; Figures S44‐S45) are provided in the Supporting Information. The authors have cited additional references within the Supporting Information [61, 62, 63].

## Data Availability

The data that support the findings of this study are available in the supplementary material of this article.
