# Supplementary material for: Light‐Induced Anion Translocation to Control Helical Folding in an Artificial Communication System
Source: Chemistry. 2026 Apr 24;32(26):e71031. doi: 10.1002/chem.71031 (PMC13356402; doi:10.1002/chem.71031)
Supplement: Supplementary file 1 — Synthetic procedures and full characterization, 1H and 13C NMR spectra of new compounds (Figures S1‐S22), 1H NMR, UV‐Vis, and CD studies, DFT calculations, and X‐ray analysis (CCDC 2470676 and 2470677; Figures S44‐S45) are provided in the Supporting Information. The authors have cited additional references within the Supporting Information [61, 62, 63]. [file CHEM-32-e71031-s001.pdf]

SUPPORTING INFORMATION

Light-Induced Anion Translocation to Control Helical Folding in and Artificial Communication System

Indigo M. Bekaert,[a] Suchismita Saha,[a] Julia Villalva,[a] David Villarón,[a] Maxime A. Siegler,[b] and Sander J. Wezenberg*[a]

[a] Leiden Institute of Chemistry, Leiden University, Einsteinweg 55, 2333 CC Leiden (The Netherlands)

[b] Department of Chemistry, Johns Hopkins University, 3400 North Charles St., Baltimore, MD 21218 (United States)

Email: s.j.wezenberg@lic.leidenuniv.nl

Table of Contents

Experimental section S2
^{1}H and ^{13}C NMR spectra of new compounds S11
^{1}H NMR titration experiment of (*S*,*S*)-**1** with $[Bu_4N]^+[Cl]^-$ S33
^{1}H NMR titration experiment of (*S*,*S*)-**1** with $[Et_4N]^+[Cl]^-$ S35
Circular dichroism absorption studies of (*S*,*S*)-**1** and (*R*,*R*)-**1** S37
CD titration experiments of (*S*,*S*)-**1** with $[Bu_4N]^+[Cl]^-$ S41
UV-vis photostability studies of foldamer **1** S45
UV-vis photoisomerization studies of receptor **2** S46
^{1}H NMR photoisomerization studies of receptor **2** S47
^{1}H NMR titration experiments of (*Z*)-**2** with $[Bu_4N]^+[Cl]^-$ S48
^{1}H NMR titration experiments of (*Z*)-**2** with $[Et_4N]^+[Cl]^-$ S50
^{1}H NMR titration experiments of (*E*)-**2** with $[Et_4N]^+[Cl]^-$ S52
X-ray crystallographic data of receptor **2** S54
CD communication experiment between (*S*,*S*)-**1** and **2** S59
^{1}H NMR communication experiment between (*S*,*S*)-**1** and **2** S60
CD control experiment of isomerization of **2** in presence of $[Et_4N]^+[Cl]^-$ S61
^{1}H NMR control experiment of isomerization of **2** in presence of $[Et_4N]^+[Cl]^-$ S62
Speciation analysis of ^{1}H NMR communication experiment S63
DFT calculations of foldamer **1** S64
References S71

Experimental section

General methods and materials:

Tetrahydrofuran, dichloromethane and methanol were dried using a Pure Solve 400 solvent purification system from Innovative Technology. Dry DMF was purchased from Acros Organics. CD_3CN and $CDCl_3$ were purchased from Eurisotop. $CDCl_3$ was filtered over basic aluminium oxide to remove DCl. The degassing of solvents was carried out by purging with N_2 for 15 min unless stated otherwise. (*S*)-3-bromo-*N*-(1-phenylethyl)benzamide and receptor **2** were prepared according to procedures reported in literature.[1,2] All other chemicals and solvents were commercial products and were used without further purification. Column chromatography was performed using silica gel (SiO_2) purchased from Screening Devices BV (pore diameter 55-70 Å, surface area 500 m^2g^{-1}) and neutral aluminum oxide (Al_2O_3) was purchased from Fluka Analytical. Thin-layer chromatography (TLC) was carried out on aluminium sheets coated with silica 60 F254 obtained from Merck. Compounds were visualized with UV light (254 nm) or by staining with potassium permanganate. ^1H and ^{13}C NMR spectra were recorded on Bruker AV 400, Bruker 500 Ultra Shield and Bruker AV 600 instruments at 295 K unless indicated otherwise. All ^1H NMR assignment is based on HSQC and COSY spectra. Chemical shifts (δ) are denoted in parts per million (ppm) relative to residual protiated solvent ($CDCl_3$: for ^1H detection, δ = 7.26 ppm; for ^{13}C detection, δ = 77.16 ppm). The splitting pattern of peaks is designated as follows: s (singlet), d (doublet), t (triplet), q (quartet), m (multiplet), br (broad). Infrared spectra were recorded on a PerkinElmer FT-IR Spectrum Two spectrometer using an ATR unit. Absorbance maxima are reported in wavenumbers (ν, cm^{-1}) and only selected intensities are reported, designated as follows: s (strong), m (medium), w (weak), very w (very weak), br (broad), and sh (shoulder). Melting points were determined with a Büchi M560 apparatus. High-resolution mass spectrometry (ESI-MS) was performed on a Thermo Scientific Q Exactive HF spectrometer with electron spray ionization (ESI). Optical rotation was recorded on an Anton Paar MCP100 polarimeter at 25 °C with cell length 10 cm. UV-vis spectra were recorded on an Agilent Cary 8454 spectrometer in a 1 cm or 1 mm quartz cuvette at 20 °C unless stated otherwise. Circular dichroism spectra were recorded on a Jasco J-815 CD spectrometer in a 1 mm quartz cuvette using the following parameters unless stated otherwise; T = 20 °C, sensitivity = 100 mdeg, band width = 1 nm, data pitch = 1 nm, response time = 1 s, 2 accumulations, 50 nm/min, 230-350 nm. The spectra were background corrected and processed with OriginPro 9.1 using a 10 p Adjacent-Averaging smoothening function.

Irradiation of UV-vis, CD and NMR samples was carried out using a Thorlabs model M340F3 LED (0.85 mW) and a Thorlabs model M365F1 LED (3.00 mW), positioned at a distance of 1 cm to the sample.

1,3-Dibromo-5-(dodecyloxy)benzene (**3**)

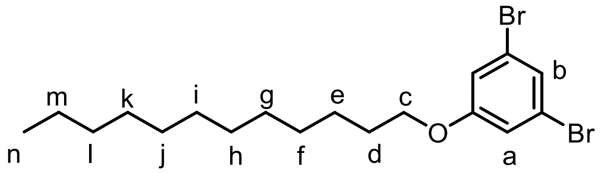

A mixture of 3,5-dibromophenol (5.00 g, 19.8 mmol) and Cs_2CO_3 (9.70 g, 29.8 mmol) in THF (150 mL) was stirred at reflux for 15 minutes under nitrogen atmosphere. Then, 1-bromododecane (15.0 mL, 62.5 mmol) was added and the mixture was stirred at reflux for a further 18 h. The mixture was allowed to cool to rt, treated with ice cold water (100 mL) and extracted with CH_2Cl_2 (3 × 100 mL). The combined organic layers were dried over Na_2SO_4 and concentrated. Purification by column chromatography (SiO_2, pentane) afforded **3** as a colorless liquid (7.30 g, 17.4 mmol, 88%). R_f = 0.84 (SiO_2, pentane). ^{1}H NMR ($CDCl_3$, 500 MHz): δ = 7.22 (t, ^4J = 1.6 Hz, 2H; H_a), 6.98 (d, ^4J = 1.6 Hz, 1H; H_b), 3.91 (t, ^3J = 6.6 Hz, 2H; H_c), 1.72-1.78 (m, 2H; H_d), 1.40-1.45 (m, 2H; H_e), 1.27-1.35 (m, 16H; $H_{f\text{-}m}$), 0.88 (t, ^3J = 6.8 Hz, 3H; H_n) ppm. ^{13}C NMR ($CDCl_3$, 126 MHz): δ = 160.5, 126.3, 123.2, 117.1, 68.8, 32.1, 29.8 (2C), 29.7 (2C), 29.5, 29.4, 29.1, 26.1, 22.8, 14.3 ppm. IR (ATR): ν = 2922 (vs), 2848 (vs), 1580 (m), 1556 (s), 1465 (m), 1439 (s), 1418 (m), 1389 (m), 1298 (w), 1252 (m), 1228 (m), 1108 (vw), 1091 (vw), 1044 (w), 1028 (s), 1001 (w), 986 (w), 887 (s), 857 (m), 840 (m), 826 (s), 741 (s), 723 (s), 666 (s), 613 (w), 571 (w) cm^{-1}. The product was poorly ionizable impeding mass spectrometry analysis.

1-Bromo-3-(dodecyloxy)-5-iodobenzene (**4**)

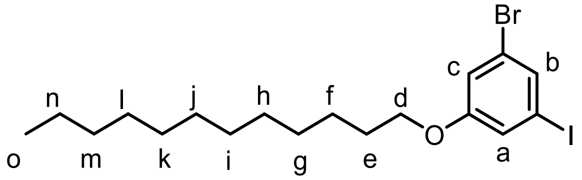

A solution of 1,3-dibromo-5-(dodecyloxy)benzene (**3**, 2.22 g, 5.28 mmol) in THF (40 mL) was cooled to –78 °C under nitrogen atmosphere. *n*-BuLi (1.6 M in hexanes, 3.63 mL, 5.81 mmol) was added dropwise over 10 min and the mixture was stirred for 2 h at –78 °C. Then, a solution of I_2 (2.69 g, 10.6 mmol) in THF (15 mL) was slowly added at –78 °C and the mixture was

allowed to warm to rt and stirred for a further 18 h. The reaction mixture was treated with a sat. aqueous solution of $Na_2S_2O_3$ (30 mL) and extracted with CH_2Cl_2 (3 × 50 mL). The combined organic layers were dried over Na_2SO_4 and concentrated. Purification by column chromatography (SiO_2, pentane) afforded **4** as a pale yellow liquid (2.22 g, 4.75 mmol, 90%). R_f = 0.78 (SiO_2, pentane). ^{1}H NMR ($CDCl_3$, 500 MHz): δ = 7.41 (t, ^4J = 1.6 Hz, 1H; H_b), 7.17 (dd, ^4J = 2.2, 1.6 Hz, 1H; H_c/H_a), 7.00 (dd, ^4J = 2.2, 1.6 Hz, 1H; H_a/H_c), 3.89 (t, ^3J = 6.6 Hz, 2H; H_d), 1.72-1.78 (m, 2H; H_e), 1.39-1.45 (m, 2H; H_f), 1.27-1.35 (m, 16H; $H_{g\text{-}n}$), 0.89 (t, ^3J = 7.0 Hz, 3; H_o) ppm. ^{13}C NMR ($CDCl_3$, 126 MHz): δ = 160.3, 131.9, 123.2, 122.9, 117.7, 94.3, 68.7, 32.1, 29.8 (2C), 29.7 (2C), 29.5 (2C), 29.1, 26.1, 22.9, 14.3 ppm. IR (ATR): ν = 2920 (s), 2846 (m), 1576 (m), 1548 (s), 1464 (m), 1434 (m), 1410 (br w), 1388 (m), 1295 (w), 1256 (m), 1228 (m), 1027 (m), 1004 (m), 983 (w), 880 (m), 858 (m), 840 (w), 826 (s), 722 (s), 667 (m), 610 (w), 566 (vw) cm^{-1}. The product was poorly ionizable impeding mass spectrometry analysis.

[{3-Bromo-5-(dodecyloxy)phenyl}ethynyl]trimethylsilane (**5**)

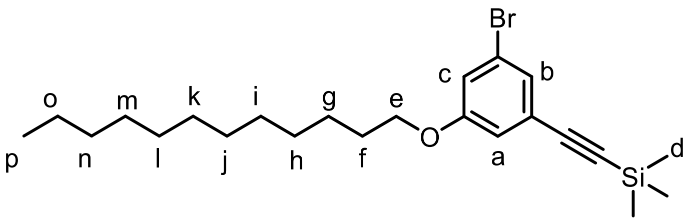

1-Bromo-3-(dodecyloxy)-5-iodobenzene (**4**, 2.00 g, 4.28 mmol), $Pd(PPh_3)_2Cl_2$ (300 mg, 0.428 mmol) and CuI (82.0 mg, 0.428 mmol) were dissolved in Et_3N (100 mL). The solution was degassed by purging with nitrogen for 30 minutes. Trimethylsilyl acetylene (0.73 mL, 5.14 mmol) was added and the resulting mixture was stirred at rt for 3 h and concentrated. Purification by column chromatography (SiO_2, pentane) afforded compound **5** as a yellow viscous liquid (1.80 g, 4.07 mmol, 95%). R_f = 0.56 (SiO_2, pentane). ^{1}H NMR ($CDCl_3$, 500 MHz): δ = 7.18 (dd, ^4J = 1.8, 1.4 Hz, 1H; H_b), 7.01 (dd, ^4J = 2.4, 1.8 Hz, 1H; H_a), 6.90 (dd, ^4J = 2.4, 1.4 Hz, 1H; H_c), 3.91 (t, ^3J = 6.6 Hz, 2H; H_e), 1.72-1.78 (m, 2H; H_f), 1.39-1.45 (m, 2H; H_g), 1.26-1.34 (m, 16H; $H_{h\text{-}o}$), 0.88 (t, ^3J = 6.8 Hz, 3H; H_p), 0.24 (s, 9H; H_d) ppm. ^{13}C NMR ($CDCl_3$, 126 MHz): δ = 159.6, 127.1, 125.5, 122.5, 119.0, 116.5, 103.5, 95.5, 68.6, 32.1, 29.8 (2C), 29.7 (2C), 29.5 (2C), 29.2, 26.1, 22.9, 14.3, 0.0 ppm. IR (ATR): ν = 628 (w), 663 (m), 676 (m) , 700 (w), 722 (w), 759 (s), 841 (s), 968 (w), 988 (w), 1040 (m), 1158 (s), 1249 (s), 1277 (s), 1307 (m), 1389 (m), 1424 (s), 1440 (m), 1466 (m), 1564 (s), 1590 (m), 2161 (w), 2853 (s), 2923 (s) cm^{-1}. The product was poorly ionizable impeding mass spectrometry analysis.

[{3-azido-5-(dodecyloxy)phenyl}ethynyl]trimethylsilane (6)

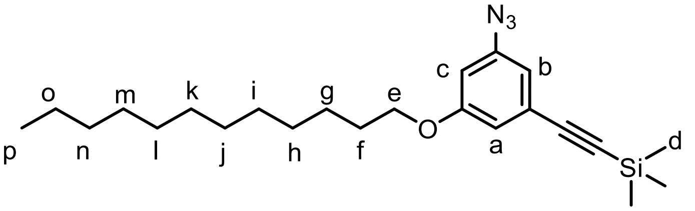

A solution of compound **5** (1.45 g, 3.31 mmol) in THF (30 mL) was cooled to –78 °C under nitrogen atmosphere. *n*-BuLi (1.6 M in hexanes, 2.28 mL, 3.66 mmol) was added dropwise over 10 min and the resulting mixture was stirred for 1 h at –78 °C. Then, 4-methylbenzenesulfonyl azide (10-15% w/v in toluene, 7.87 mL) diluted in THF (10 mL) was slowly added. The mixture was allowed to warm to rt and stirred for a further 18 h, after which it was treated with ice-cold water (50 mL). The mixture was extracted with CH_2Cl_2 (3 × 50 mL) aided by addition of brine (20 mL). The combined organic layers were dried over Na_2SO_4 and concentrated. Purification by column chromatography (First: SiO_2, CH_2Cl_2/pentane 1:19, second: neutral Al_2O_3, CH_2Cl_2/pentane 1:99) afforded **6** as a pale yellow solid (661 mg, 1.66 mmol, 50%). R_f = 0.50 (SiO_2, CH_2Cl_2/pentane 1:19). m.p. 34.5-35.4 °C. ^{1}H NMR ($CDCl_3$, 500 MHz): δ = 6.76 (dd, ^4J = 2.2, 1.2 Hz, 1H; H_b/H_a), 6.73 (dd, ^4J = 2.2, 1.2 Hz, 1H; H_a/H_b), 6.50 (t, ^4J = 2.2 Hz, 1H; H_c), 3.92 (t, ^3J = 6.6 Hz, 2H; H_e), 1.73-1.78 (m, 2H; H_f), 1.40-1.46 (m, 2H; H_g), 1.27-1.33 (m, 16H; $H_{h\text{-}o}$), 0.89 (t, ^3J = 6.8 Hz, 3H; H_p), 0.25 (s, 9H; H_d) ppm. ^{13}C NMR ($CDCl_3$, 126 MHz): δ = 160.1, 141.4, 125.3, 114.8, 114.3, 106.8, 104.1, 95.1, 68.5, 32.1, 29.8 (2C), 29.7 (2C), 29.5 (2C), 29.2, 26.1, 22.8, 14.3, 0.0 ppm. IR (ATR): ν = 614 (w), 632 (s), 677 (s), 701 (m), 724 (s), 733 (s), 759 (s), 790 (m), 835 (s), 855 (s), 976 (s), 992 (s), 1013 (m), 1040 (s), 1048 (s), 1110 (s), 1169 (s), 1238 (s), 1251 (s), 1314 (m), 1336 (w), 1397 (m), 1422 (s), 1434 (m), 1447 (m), 1469 (s), 1587 (s), 1733 (w), 2109 (s), 2168 (w), 2849 (s), 2874 (sh), 2918 (s), 2956 (sh) cm^{-1}. The product was poorly ionizable impeding mass spectrometry analysis.

4,4'-[5-(Dodecyloxy)-1,3-phenylene]bis[1-{3-(dodecyloxy)-5-[(trimethylsilyl)ethynyl] phenyl}-1H-1,2,3-triazole] (**7**)

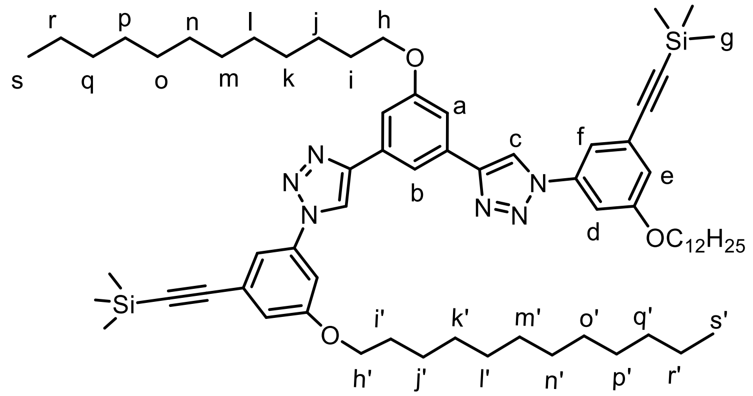

1-(Dodecyloxy)-3,5-diethynylbenzene (**10**, 50.0 mg, 161 μmol), [{3-azido-5-(dodecyloxy) phenyl}ethynyl]trimethylsilane (**6**, 161 mg, 403 μmol), $[Cu(CH_3CN)_4]PF_6$ (12.0 mg, 32.0 μmol) and TBTA (17.0 mg, 32.0 μmol) were dissolved in CH_2Cl_2 (30 mL) under nitrogen atmosphere. The mixture was stirred at reflux for 36 h, treated with H_2O (50 mL) and extracted with CH_2Cl_2 (3 × 30 mL). The combined organic layers were dried over Na_2SO_4 and concentrated. Purification by column chromatography (neutral Al_2O_3, pentane/CH_2Cl_2 1:1) afforded **7** as a white solid (120 mg, 108 μmol, 67%). $R_f = 0.55$ (SiO_2, CH_2Cl_2). m.p. 60.3-61.2 °C. ^{1}H NMR ($CDCl_3$, 500 MHz): δ = 8.26 (s, 2H; H_c), 7.91 (t, ^4J = 1.4 Hz, 1H; H_b), 7.46 (d, ^4J = 1.4 Hz, 2H; H_a), 7.42 (dd, ^4J = 2.2, 1.2 Hz, 2H; H_f), 7.37 (t, ^4J = 2.2 Hz, 2H; H_d), 7.03 (dd, ^4J = 2.2, 1.2 Hz, 2H; H_e), 4.08 (t, ^3J = 6.6 Hz, 2H; H_h), 4.02 (t, ^3J = 6.6 Hz, 4H; $H_{h'}$), 1.77-1.83 (m, 6H; H_i, $H_{i'}$), 1.43-1.49 (m, 6H; H_j, $H_{j'}$), 1.26-1.37 (m, 48H; $H_{k\text{-}r}$, $H_{k'\text{-}r'}$), 0.87 (t, ^3J = 7.0 Hz, 9H; H_s, $H_{s'}$), 0.28 (s, 18H; H_g) ppm. ^{13}C NMR ($CDCl_3$, 126 MHz): δ = 160.3, 160.0, 148.1, 137.8, 132.0, 125.6, 118.1, 117.9, 115.5 (2C), 111.9, 107.5, 103.6, 96.1, 68.8, 68.5, 32.1, 32.0, 29.8 (6C), 29.7 (2C), 29.6, 29.5 (2C), 29.4, 29.2 (2C), 26.2, 26.1, 22.8 (2C), 14.2 (2C), 0.0 ppm. IR (ATR): ν = 636 (w), 679 (m), 723 (sh), 750 (s), 758 (s), 797 (w), 841 (s), 1002 (m), 1039 (s), 1060 (s), 1160 (s), 1206 (s), 1227 (s), 1250 (s), 1260 (s), 1276 (s), 1332 (w), 1357 (w), 1390 (w), 1441 (sh), 1465 (m), 1585 (s), 2160 (w), 2852 (s), 2922 (s) cm^{-1}. HRMS (ESI) m/z: 1109.7776 ([M+H]+, calcd for $C_{68}H_{105}N_6O_3Si_2^+$: 1109.7781).

4,4'-[5-(Dodecyloxy)-1,3-phenylene]bis[1-{3-(dodecyloxy)-5-ethynylphenyl}-1H-1,2,3-triazole] (8)

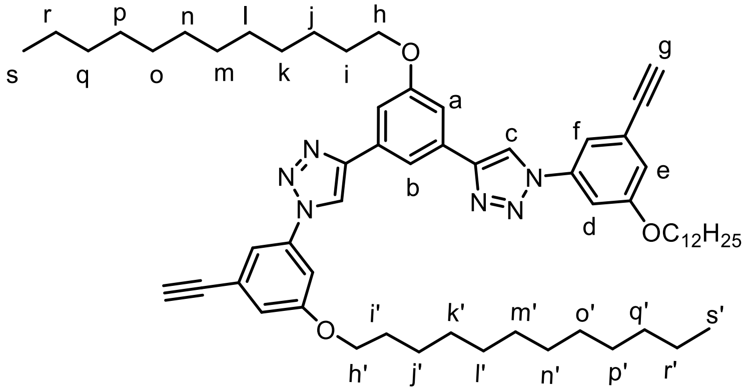

A solution of TBAF•3H_2O (85.3 mg, 270 μmol) in CH_2Cl_2 (10 mL) was added slowly to compound **7** (100 mg, 90.1 μmol) in CH_2Cl_2 (30 mL) at 0 °C and the mixture was stirred for 2 h. The mixture was extracted with H_2O (4 × 50 mL) and the combined aqueous layers were extracted with CH_2Cl_2 (4 × 50 mL). The combined organic layers were dried over Na_2SO_4 and concentrated to afford **8** as a white solid (83 mg, 86 μmol, 95%). R_f = 0.50 (SiO_2, CH_2Cl_2). m.p. 57.0-58.2 °C. ^{1}H NMR ($CDCl_3$, 500 MHz): δ = 8.25 (s, 2H, H_c), 7.88 (t, ^4J = 1.4 Hz, 1H; H_b), 7.44 (d, ^4J = 1.4 Hz, 2H; H_a), 7.43 (t, ^4J = 1.4 Hz, 2H; H_f), 7.37 (t, ^4J = 2.2 Hz, 2H; H_d), 7.03-7.04 (m, 2H; H_e), 4.06 (t, ^3J = 6.6 Hz, 2H; H_h), 4.01 (t, ^3J = 6.6 Hz, 4H; $H_{h'}$), 3.14 (s, 2H; H_g), 1.77-1.83 (m, 6H; H_i, $H_{i'}$), 1.43-1.49 (m, 6H; H_j, $H_{j'}$), 1.26-1.37 (m, 48H; $H_{k\text{-}r}$, $H_{k'\text{-}r'}$), 0.87 (t, ^3J = 7.0 Hz, 9H; H_s, $H_{s'}$) ppm. ^{13}C NMR ($CDCl_3$, 126 MHz): δ = 160.3, 160.1, 148.1, 137.9, 131.9, 124.5, 118.3, 118.1, 115.6, 115.4, 111.9, 107.7, 82.4, 78.7, 68.8, 68.5, 32.1, 32.0, 29.8 (5C), 29.7 (3C), 29.6, 29.5 (3C), 29.2 (2C), 26.2, 26.1, 22.8 (2C), 14.2 (2C) ppm. IR (ATR): ν = 625 (m), 647 (s), 677 (m), 721 (m), 763 (w), 797 (m), 840 (s), 863 (sh), 894 (w), 1004 (sh), 1038 (s), 1060 (s), 1146 (m), 1172 (s), 1207 (sh), 1225 (m), 1282 (m), 1298 (m), 1324 (w), 1349 (w), 1390 (w), 1466 (s), 1586 (s), 2158 (br, very w), 2852 (s), 2921 (s), 3310 (w) cm^{-1}. HRMS (ESI) m/z: 965.6985 ([M+H]+, calcd for $C_{62}H_{89}N_6O_3^+$: 965.6991).

[{5-(Dodecyloxy)-1,3-phenylene}bis(ethyne-2,1-diyl)]bis(trimethylsilane) (9)

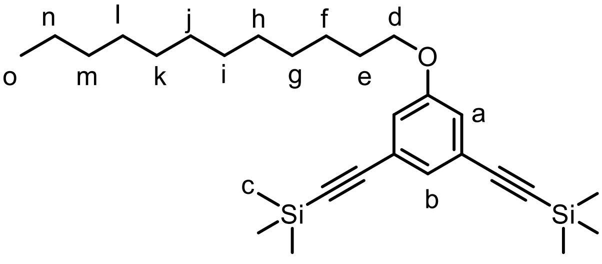

1,3-Dibromo-5-(dodecyloxy)benzene **3** (800 mg, 1.90 mmol), $Pd(PPh_3)_2Cl_2$ (267 mg, 0.381 mmol) and CuI (73.0 mg, 0.381 mmol) were dissolved in Et_3N (30 mL). The solution was degassed by purging with nitrogen for 15 minutes. Trimethylsilyl acetylene (1.10 mL, 7.61 mmol) was added and the solution was stirred at 70 °C for 12 h. The solvent was evaporated and purification by column chromatography (SiO_2, pentane) afforded compound **9** as a pale yellow liquid (777 mg, 1.71 mmol, 90%). R_f = 0.23 (SiO_2, pentane). ^{1}H NMR ($CDCl_3$, 500 MHz): δ = 7.17 (t, ^4J = 1.4 Hz, 1H; H_b), 6.93 (d, ^4J = 1.4 Hz, 2H; H_a), 3.91 (t, ^3J = 6.6 Hz, 2H; H_d), 1.71-1.77 (m, 2H; H_e), 1.39-1.45 (m, 2H; H_f), 1.26-1.33 (m, 16H; H_{g-n}), 0.88 (t, ^3J = 6.8 Hz, 3H; H_o), 0.23 (s, 18H; H_c) ppm. ^{13}C NMR ($CDCl_3$, 126 MHz): δ = 158.7, 128.1, 124.3, 118.4, 104.3, 94.6, 68.4, 32.1, 29.8 (2C), 29.7 (2C), 29.5 (2C), 29.3, 26.1, 22.9, 14.3, 0.0 ppm. IR (ATR): ν = 2956 (w), 2924 (m), 2852 (w), 2161 (w), 2153 (sh), 1579 (m), 1469 (vw), 1417 (w), 1330 (w), 1298 (vw), 1248 (s), 1167 (s), 1049 (m), 984 (m), 839 (vs), 761 (s), 722 (m), 702 (m), 681 (s), 653 (s), 638 (sh), 586 (vw) cm^{-1}. HRMS (ESI) m/z: 455.3137 ([M+H]+, calcd for $C_{28}H_{47}O_1Si_2^+$: 455.3160).

1-(Dodecyloxy)-3,5-diethynylbenzene (10)

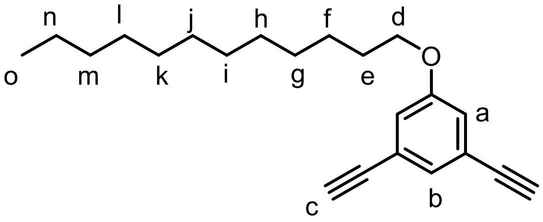

Compound **9** (750 mg, 1.65 mmol) and K_2CO_3 (1.14 g, 8.24 mmol) were dispersed in a mixture of THF (10 mL), MeOH (10 mL) and H_2O (10 mL) and stirred at rt for 2 h. Solvents were evaporated and the residue was extracted with CH_2Cl_2 (3 × 50 mL). The combined organic layers were washed with water (100 mL), dried over Na_2SO_4 and concentrated to obtain **10** as a yellow solid (487 mg, 1.57 mmol, 95%). R_f = 0.32 (SiO_2, pentane). ^{1}H NMR ($CDCl_3$, 500 MHz): δ = 7.20 (t, ^4J = 1.4 Hz, 1H; H_b), 7.00 (d, ^4J = 1.4 Hz, 2H; H_a), 3.93 (t, ^3J = 6.6 Hz, 2H; H_d), 3.05 (s, 2H; H_c), 1.73-1.79 (m, 2H; H_e), 1.42-1.46 (m, 2H; H_f), 1.27-1.40 (m, 16H; H_{g-n}), 0.89 (t, ^3J = 6.8 Hz, 3H; H_o) ppm. ^{13}C NMR ($CDCl_3$, 126 MHz): δ = 158.9, 128.2, 123.5, 119.0, 82.8, 77.7, 68.4, 32.1, 29.8 (2C), 29.7 (2C), 29.5 (2C), 29.2, 26.1, 22.8, 14.3 ppm. IR (ATR): ν = 3278 (s), 2952 (m), 2921 (s), 2847 (m), 1590 (sh), 1581 (m), 1463 (w), 1420 (w), 1393 (vw), 1324 (m), 1296 (m), 1249 (w), 1160 (s), 1147 (w), 1050 (m), 954 (w), 869 (m), 852 (m), 841 (w), 725 (w), 686 (w), 671 (s), 629 (s), 607 (w) cm^{-1}. HRMS (ESI) m/z: 311.2370 ([M+H]+, calcd for $C_{22}H_{31}O_1^+$: 311.2369).

(*S*)-/(*R*)-3-azido-*N*-(1-phenylethyl)benzamide (11):

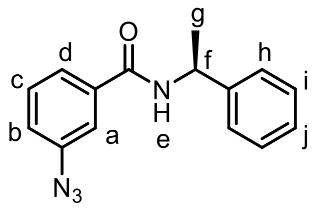

n-Butyllithium (1.6 M solution in hexanes, 1.3 mL, 2.1 mmol) was slowly added to (*S*)-3-bromo-*N*-(1-phenylethyl)benzamide (200 mg, 0.657 mmol) in degassed THF (17 mL) under nitrogen atmosphere at –78 °C. The mixture was stirred at this temperature for 2 h after which 4-methylbenzenesulfonyl azide (10-15%wt in toluene, 2 mL, ~1.0 mmol) was added. After 50 min of stirring, the mixture was allowed to warm to rt and stirred for a further 2.5 h. The mixture was treated with H_2O (30 mL) and extracted with EtOAc (3 × 30 mL). The combined organic layers were washed with brine (50 mL), dried over Na_2SO_4, and concentrated. Purification by column chromatography (SiO_2, EtOAc/pentane 1:4) yielded compound (*S*)-**11** as an off-white solid (84 mg, 0.32 mmol, 48%). The enantiomer (*R*)-**11** (86 mg, 0.32 mmol, 49%) was obtained through the same procedure starting with (*R*)-3-bromo-*N*-(1-phenylethyl)benzamide. R_f = 0.38 (SiO_2, EtOAc/pentane 1:4); m.p. 141.4-142.1 °C; (*R*)-**11** $[\alpha]_D^{25}$ +28°(*c* 0.80, $CHCl_3$), (*S*)-**11** $[\alpha]_D^{25}$ –28°(*c* 0.50, $CHCl_3$); ^{1}H NMR (600 MHz, $CDCl_3$): δ = 7.42-7.40 ppm (m, 2 H; H_a, H_j), 7.34-7.28 ppm (m, 5 H; H_c, H_h, H_i), 7.22 ppm (dt, ^4J = 6.6, 1.8 Hz, 1 H; H_d), 7.07 ppm (ddd, ^3J = 8.4,^4J = 2.4, 1.2 Hz, 1 H; H_b), 6.32 ppm (d, ^3J = 7.8 Hz, 1 H; H_e), 5.32 ppm (dq, ^3J = 7.8, 7.2 Hz, 1 H; H_f), 1.61 ppm (d, ^3J = 7.2 Hz, 3 H; H_g); ^{13}C NMR (151 MHz, $CDCl_3$): δ = 165.7, 143.0, 141.0, 136.5, 130.1, 129.0, 127.7, 126.4, 123.1, 122.0, 118.1, 49.6, 21.8; IR (ATR): ν= 697 (s), 753 (m), 811 (m), 897 (m), 1096 (m), 1124 (m), 1156 (s), 1298 (s), 1147 (m), 1491 (m), 1538 (s), 1585 (m), 1634 (s), 2118 (s), 2871 (w), 2930 (w), 2961 (w), 3061 (w), 3260 (m), 3302 (m), 3355 (m) cm^{-1}; HRMS (ESI) m/z: 267.12397 ([M+H]+, calcd for $C_{15}H_{15}N_4O^+$: 267.12404).

Foldamer 1:

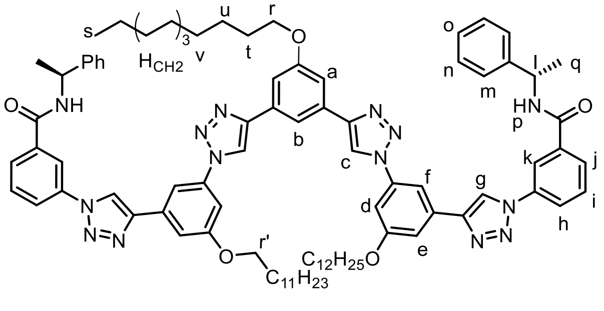

A solution of **8** (6.0 mg, 6.2 μmol) in CH_2Cl_2/MeOH (1.2 mL, 5:1 w/w) was added to (*S*)-**11** (7.2 mg, 27.0 μmol), $[Cu(CH_3CN)_4]PF_6$ (1.8 mg, 4.8 μmol) and tris((1-benzyl-4-triazolyl)methyl)amine (1.5 mg, 2.8 μmol) in CH_2Cl_2 (1 mL) under an argon atmosphere. The mixture was heated to 40 °C for 22 h, allowed to cool to rt, diluted with CH_2Cl_2 (8 mL) and treated with Chelex(Na). The mixture was filtered, and the filtrate was washed with H_2O (3 × 10 mL), dried over Na_2SO_4, and concentrated. Precipitation from CH_2Cl_2/MeOH afforded (*S,S*)-**1** (38 mg, 25.4 μmol, 78%) as a white solid. (*R,R*)-**1** (9 mg, 6.0 μmol, 97%) was obtained through the same procedure starting with (*R*)-**11**. R_f = 0.25 (SiO_2, 2% MeOH in CH_2Cl_2); m.p. 215.8-216.3 °C; (*R,R*)-**1** $[\alpha]_D^{25}$ –5.7°(*c* 0.30, $CHCl_3$), (*S,S*)-**1** $[\alpha]_D^{25}$ +5.8°(*c* 1.00, $CHCl_3$); ^{1}H NMR (600 MHz, $CDCl_3$): δ = 8.32 ppm (s, 2 H; H_g), 8.28 ppm (s, 2 H; H_c), 8.20 ppm (s, 2H; H_k), 7.95 ppm (d, ^3J = 8.4 Hz, 2H; H_h), 7.85 ppm (d, ^3J = 7.8 Hz, 2H; H_j), 7.79 ppm (s, 1H; H_b), 7.66 ppm (s, 2H; H_f), 7.54 ppm (m, 2H; H_i), 7.51 ppm (s, 2H; H_e), 7.44 ppm (s, 2H; H_a), 7.39-7.37 ppm (m, 6H; H_d, H_m), 7.28-7.25 ppm (m, 4H; H_n), 7.23 ppm (d, ^3J = 7.8 Hz, 2H; H_p) 7.22-7.19 ppm (m, 2H; H_o), 5.30 ppm (dq, ^3J = 7.2, 6.6 Hz, 2H; H_l), 4.08 ppm (t, ^3J = 6.6 Hz, 4 H; $H_{r'}$), 4.04 ppm (t, ^3J = 6.6 Hz, 2 H; H_r), 1.86-1.79 ppm (m, 6H; H_t), 1.60 ppm (d, ^3J = 7.2 Hz, 6H; H_q), 1.52-1.46 ppm (m, 6H; H_u), 1.41-1.36 ppm (m, 6H; H_v), 1.29-1.25 ppm (m, 42H; H_{CH2}), 0.87 ppm (t, ^3J = 7.2 Hz, 9H; H_s); ^{13}C NMR (151 MHz, $CDCl_3$): δ = 165.4 ppm (C_q), 160.8 ppm (C_q), 160.3 ppm (C_q), 148.0 ppm (C_q), 147.4 ppm (C_q), 143.1 ppm (C_q), 138.3 ppm (C_q), 137.0 ppm (C_q), 136.4 ppm (C_q), 132.7 ppm (C_q), 131.8 ppm (C_q), 130.3 ppm (C-i), 128.9 ppm (C-n), 127.7 ppm (C-o), 126.5 ppm (C-m), 123.3 ppm (C-h/C-j), 118.9 ppm (C-k), 118.7 ppm (C-c/C-g), 118.2 ppm (C-c/C-g), 115.3 ppm (C-b), 111.9 ppm (C-a), 111.8 ppm (C-e), 109.1 ppm (C-f), 106.7 ppm (C-d), 68.9 ppm (C-r), 68.5 ppm (C-r'), 50.0 ppm (C-l), 32.1 ppm ($C-CH_2$), 29.8 ppm ($C-CH_2$), 26.2 ppm ($C-CH_2$), 22.9 ppm ($C-CH_2$), 21.8 ppm (C-q), 14.3 ppm (C-s); IR (ATR): ν = 681 (s), 699 (s), 720 (w), 755 (m), 798 (m), 857 (w), 1038 (s), 1167 (s), 1211 (m), 1261 (m), 1345 (w), 1378 (w), 1456 (s), 1489 (m), 1536 (m), 1591 (s), 1639 (m), 2853 (s), 2922 (s), 3065 (vw), 3286 (w) cm^{-1}; ESI-MS m/z: 1520.54 ([M+Na]+, calcd for $C_{92}H_{116}N_{14}O_5Na^+$: 1520.92). ESI-HRMS was not successful for this compound, therefore ESI-MS was obtained from a Thermo Fisher LTQ-XL spectrometer with ion trap and electron spray ionization.

^{1}H and ^{13}C NMR spectra of new compounds

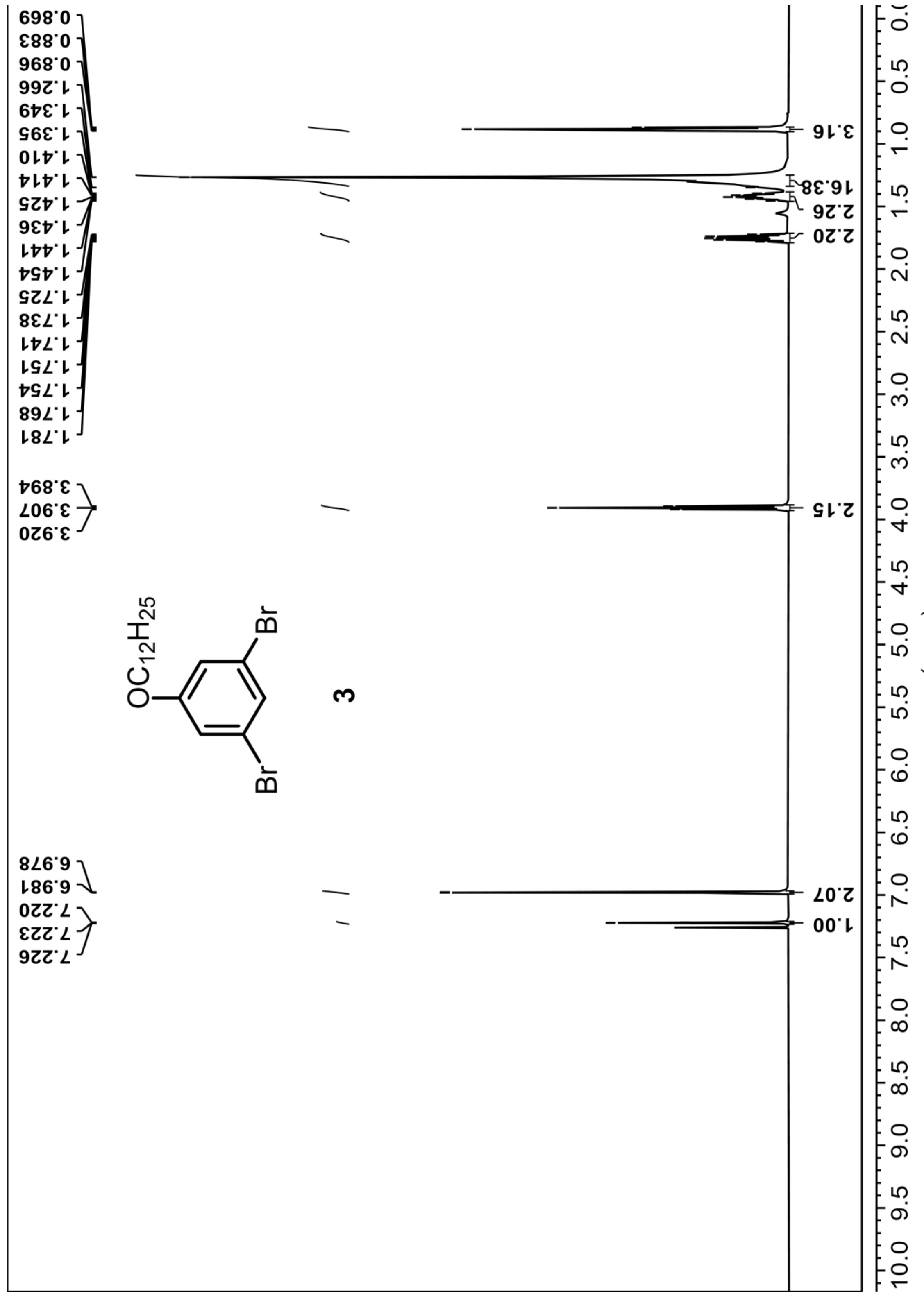

Figure S1. ^{1}H NMR spectrum of compound **3** in $CDCl_3$ (500 MHz).

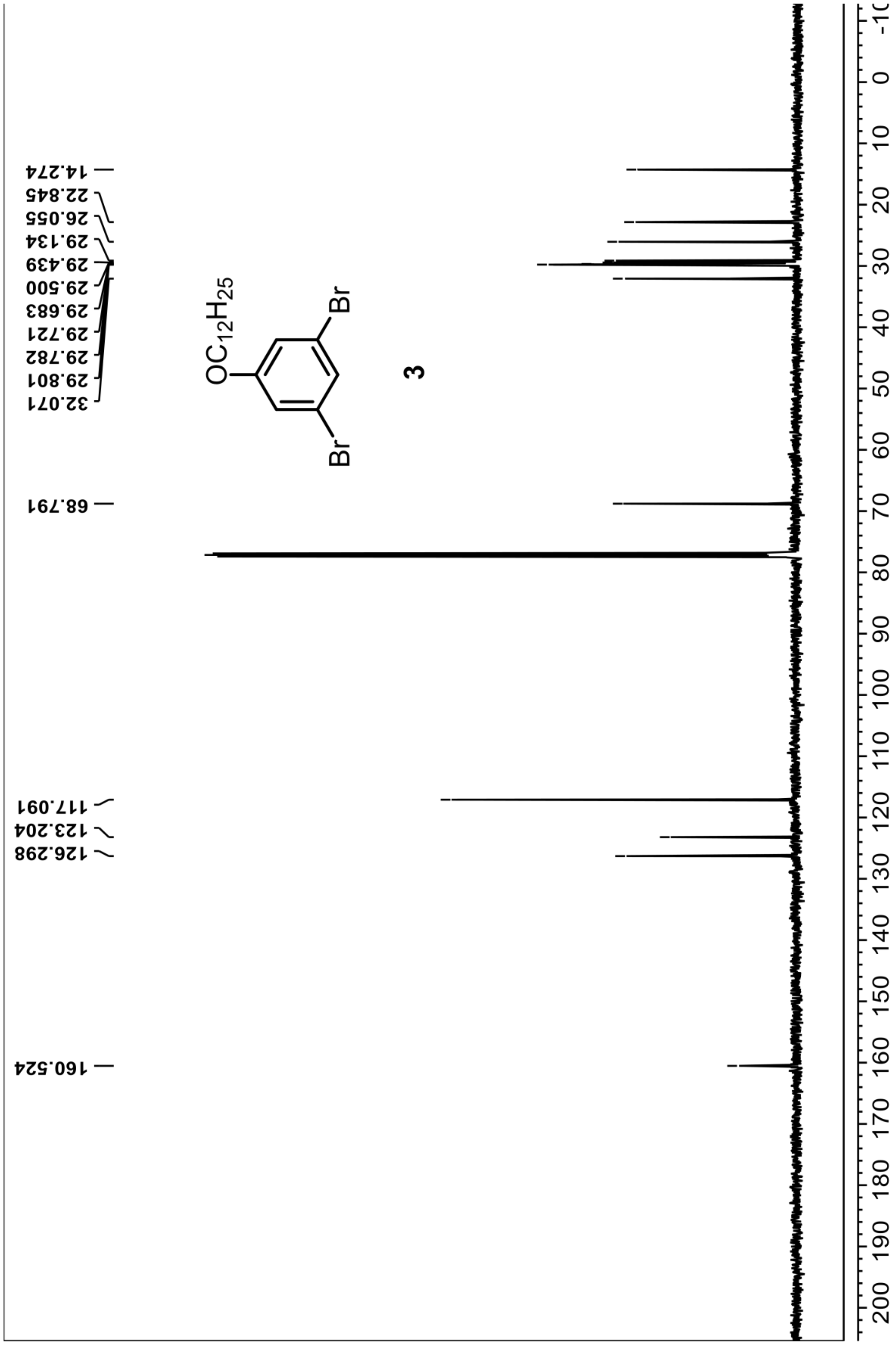

Figure S2. ^{13}C NMR spectrum of compound **3** in $CDCl_3$ (126 MHz).

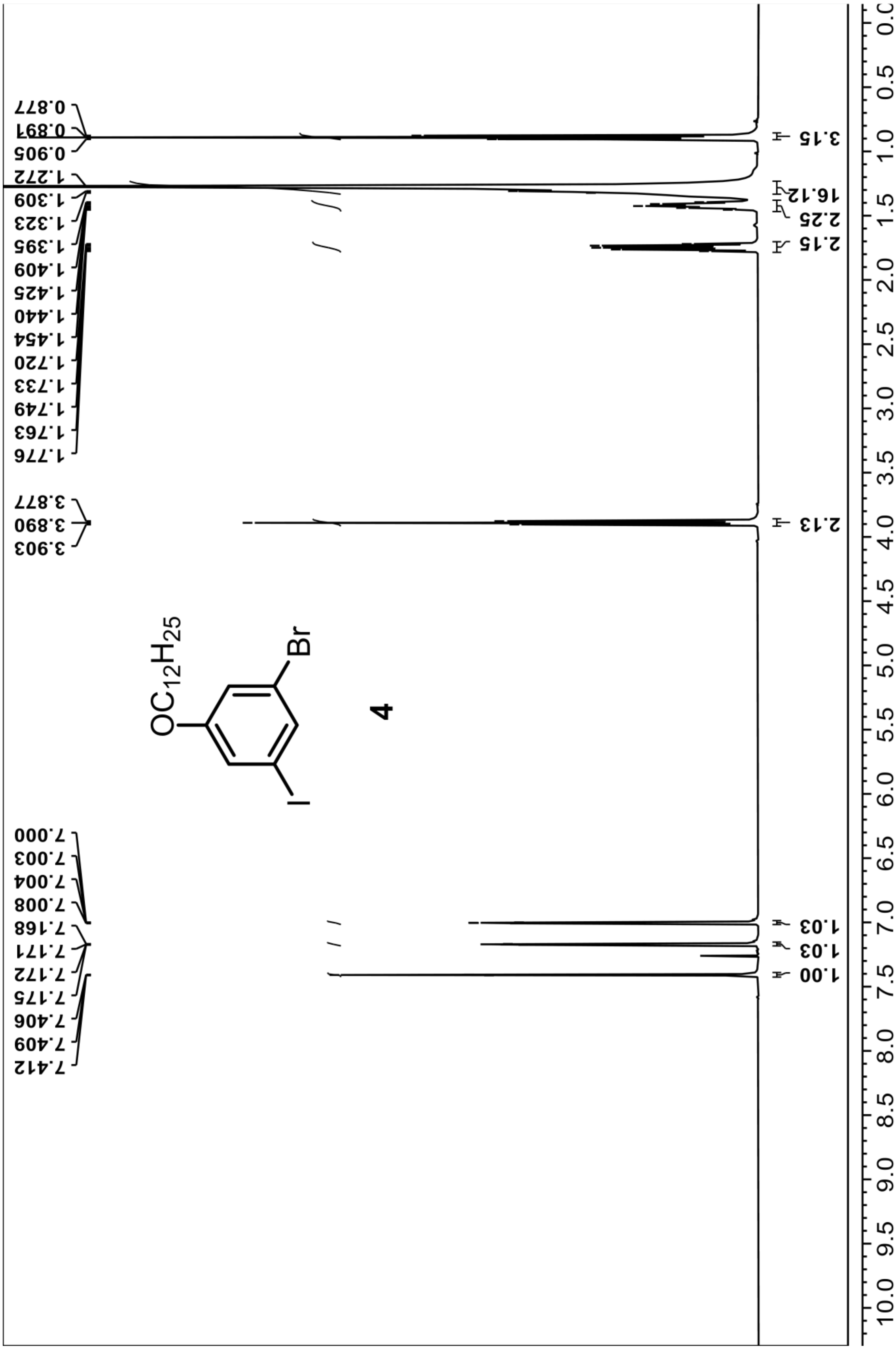

Figure S3. ^{1}H NMR spectrum of compound **4** in $CDCl_3$ (500 MHz).

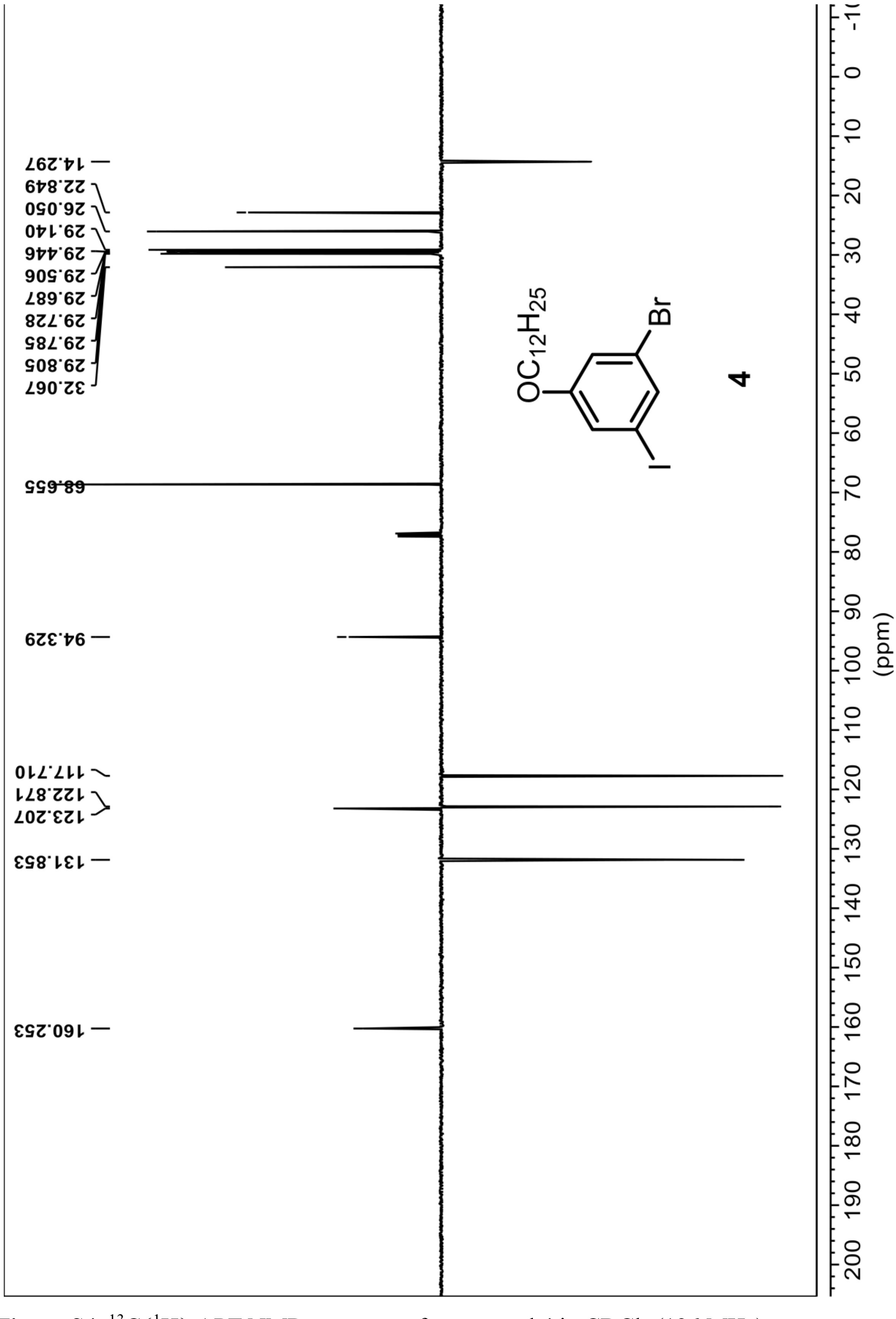

Figure S4. $^{13}C\{^{1}H\}$ APT NMR spectrum of compound **4** in $CDCl_3$ (126 MHz).

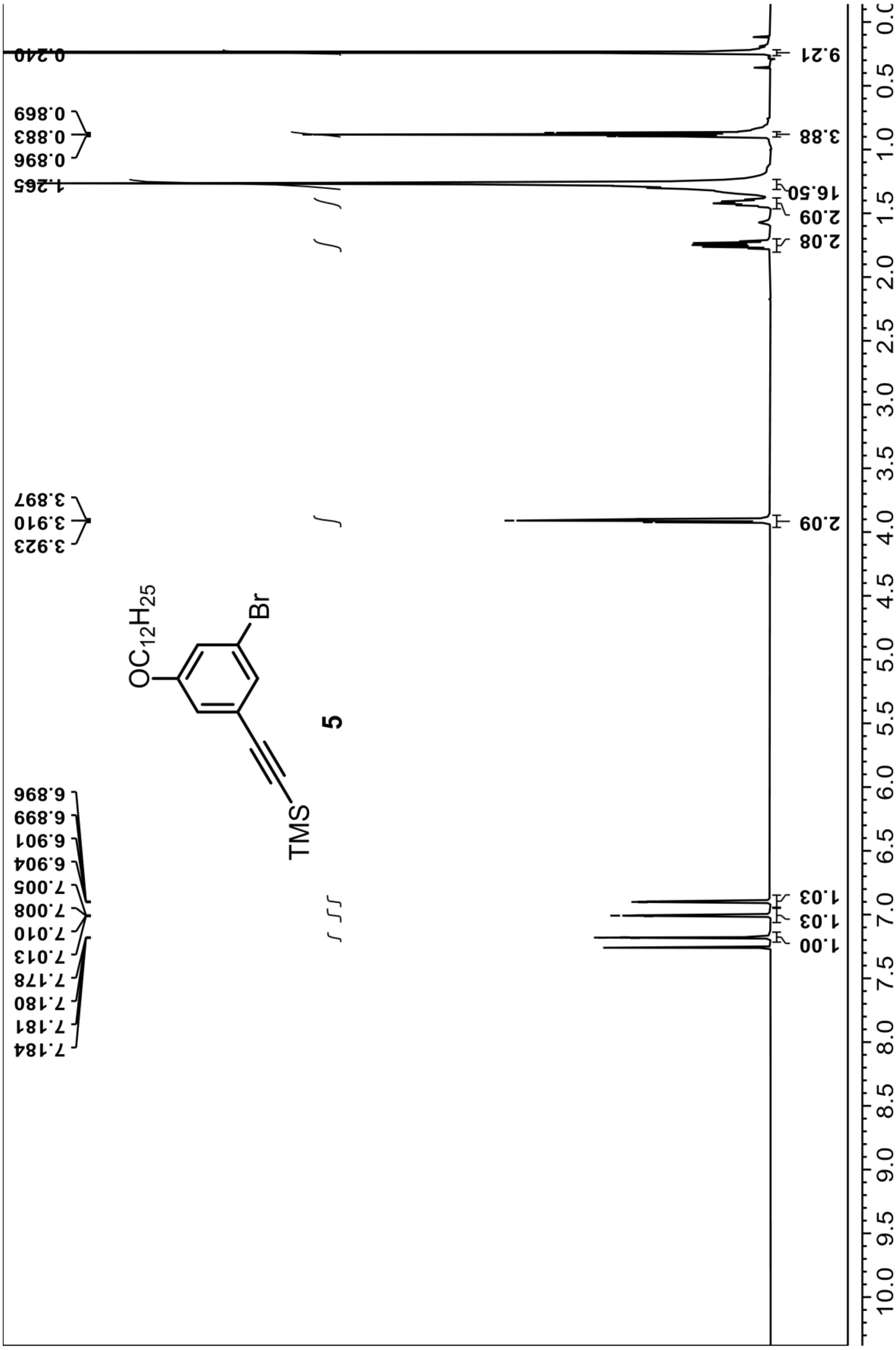

Figure S5. ^{1}H NMR spectrum of compound **5** in $CDCl_3$ (500 MHz).

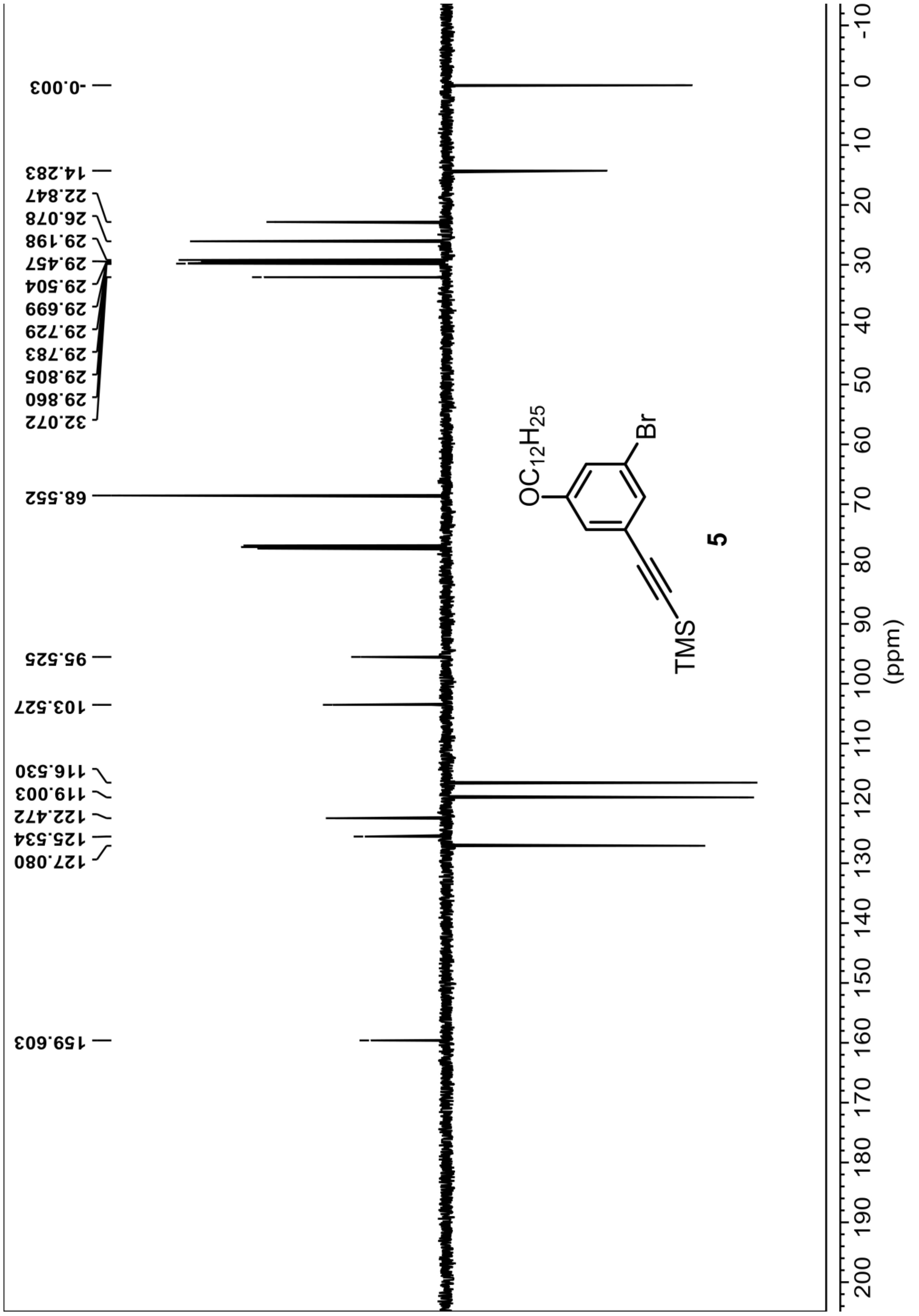

Figure S6. $^{13}C\{^{1}H\}$ APT NMR spectrum of compound **5** in $CDCl_3$ (126 MHz).

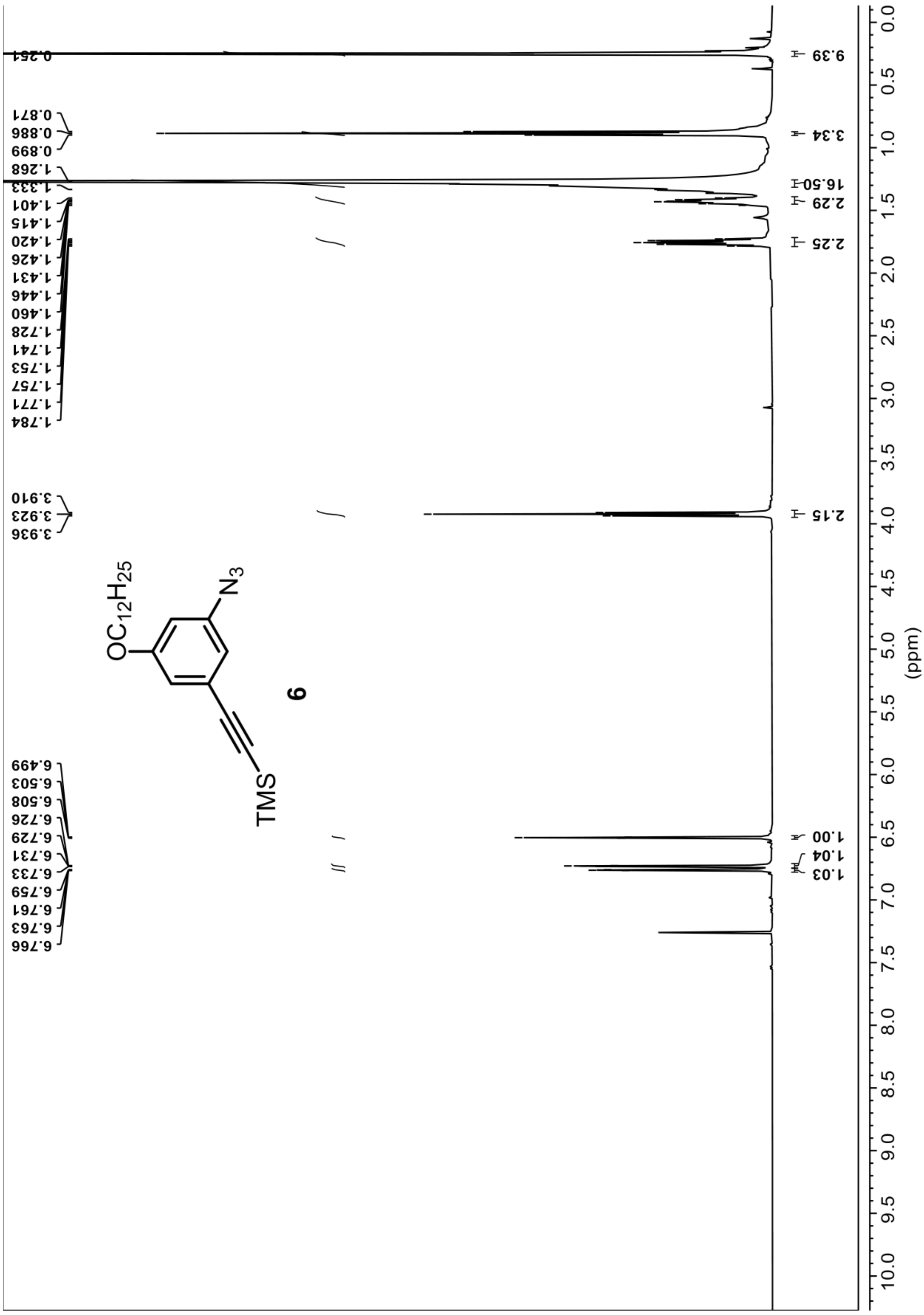

Figure S7. ^{1}H NMR spectrum of compound **6** in $CDCl_3$ (500 MHz).

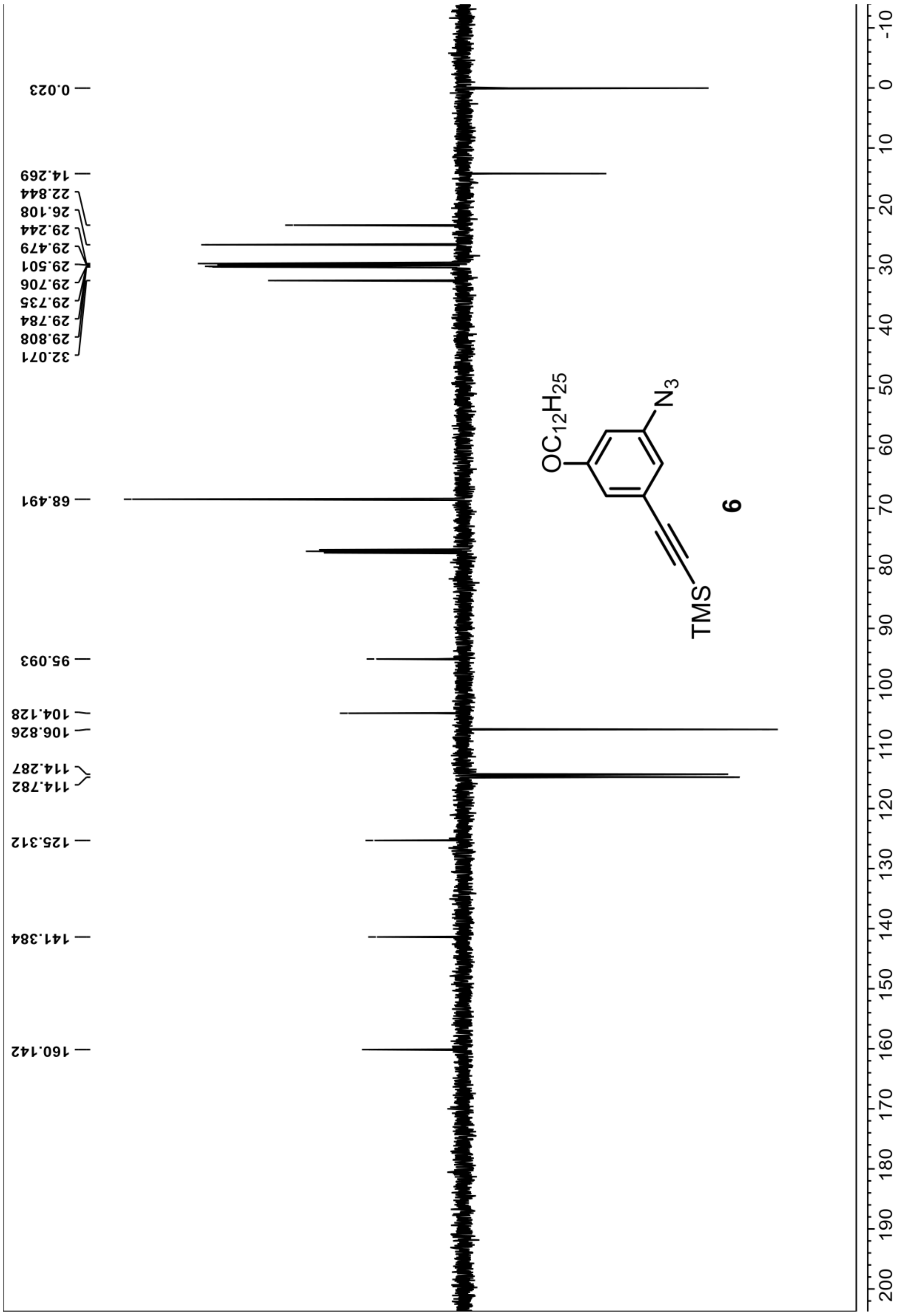

Figure S8. $^{13}C\{^1H\}$ APT NMR spectrum of compound **6** in $CDCl_3$ (126 MHz).

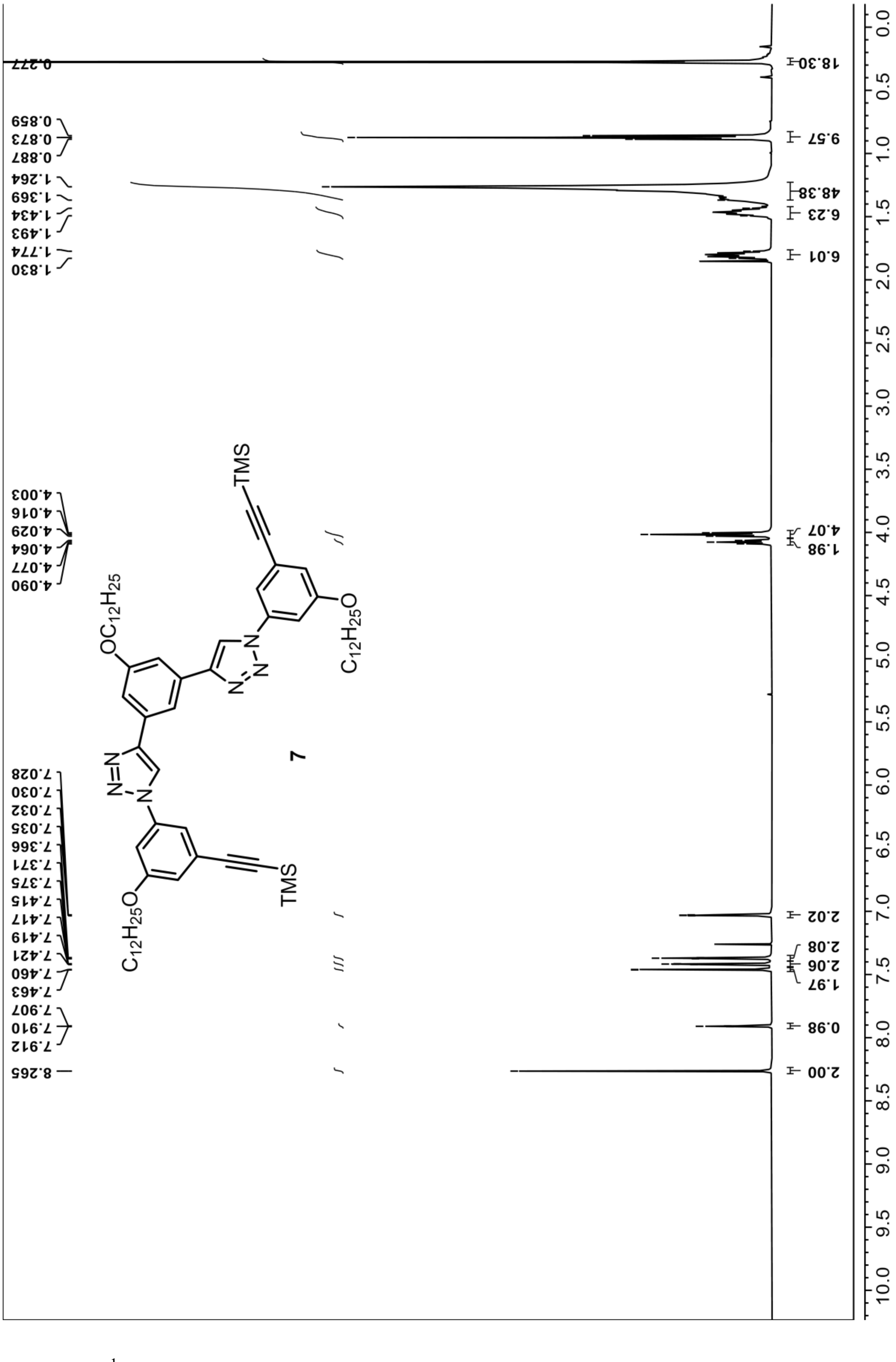

Figure S9. ^{1}H NMR spectrum of compound **7** in $CDCl_3$ (500 MHz).

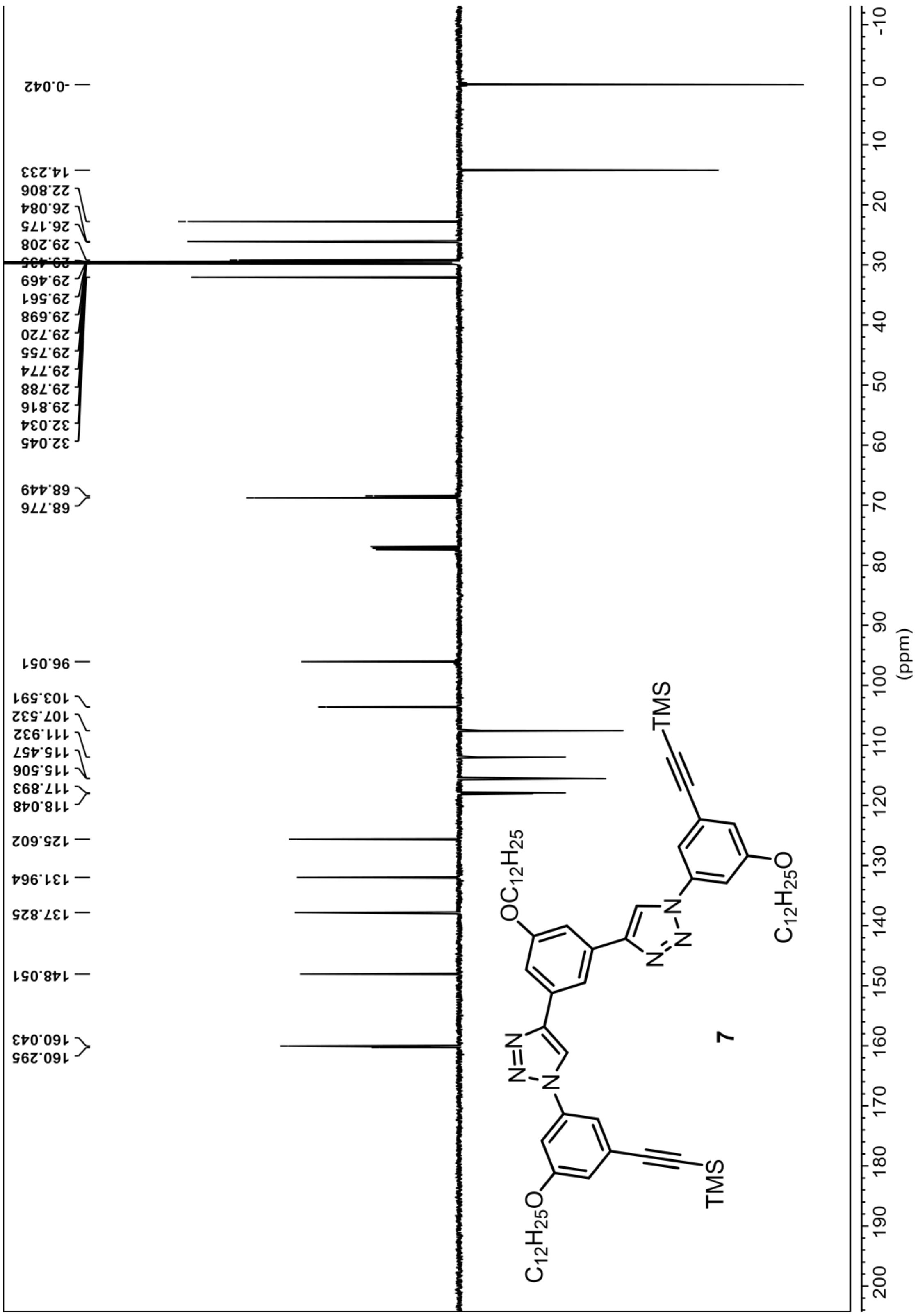

Figure S10. $^{13}C\{^1H\}$ APT NMR spectrum of compound **7** in $CDCl_3$ (126 MHz).

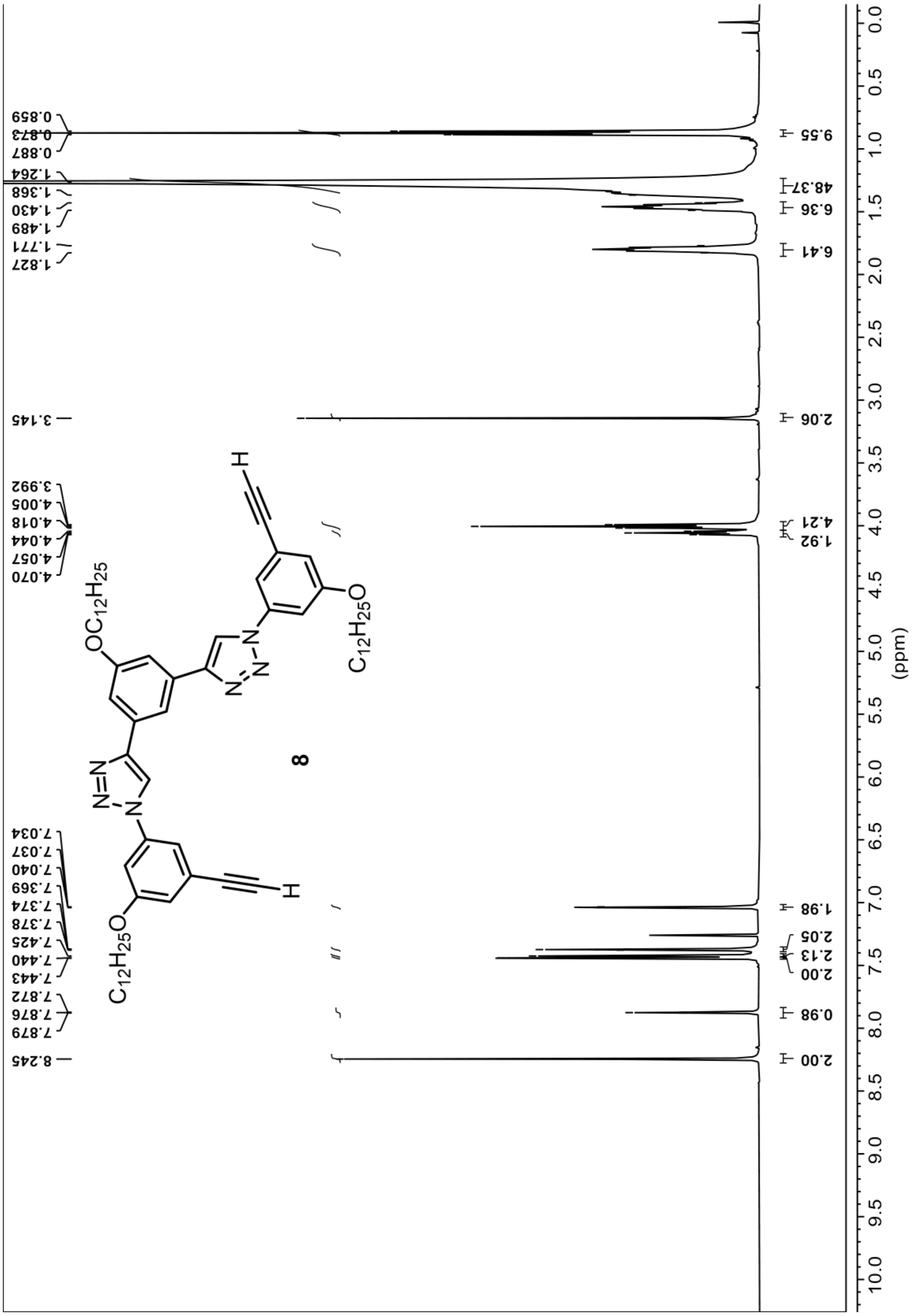

Figure S11. ^{1}H NMR spectrum of compound **8** in $CDCl_3$ (500 MHz).

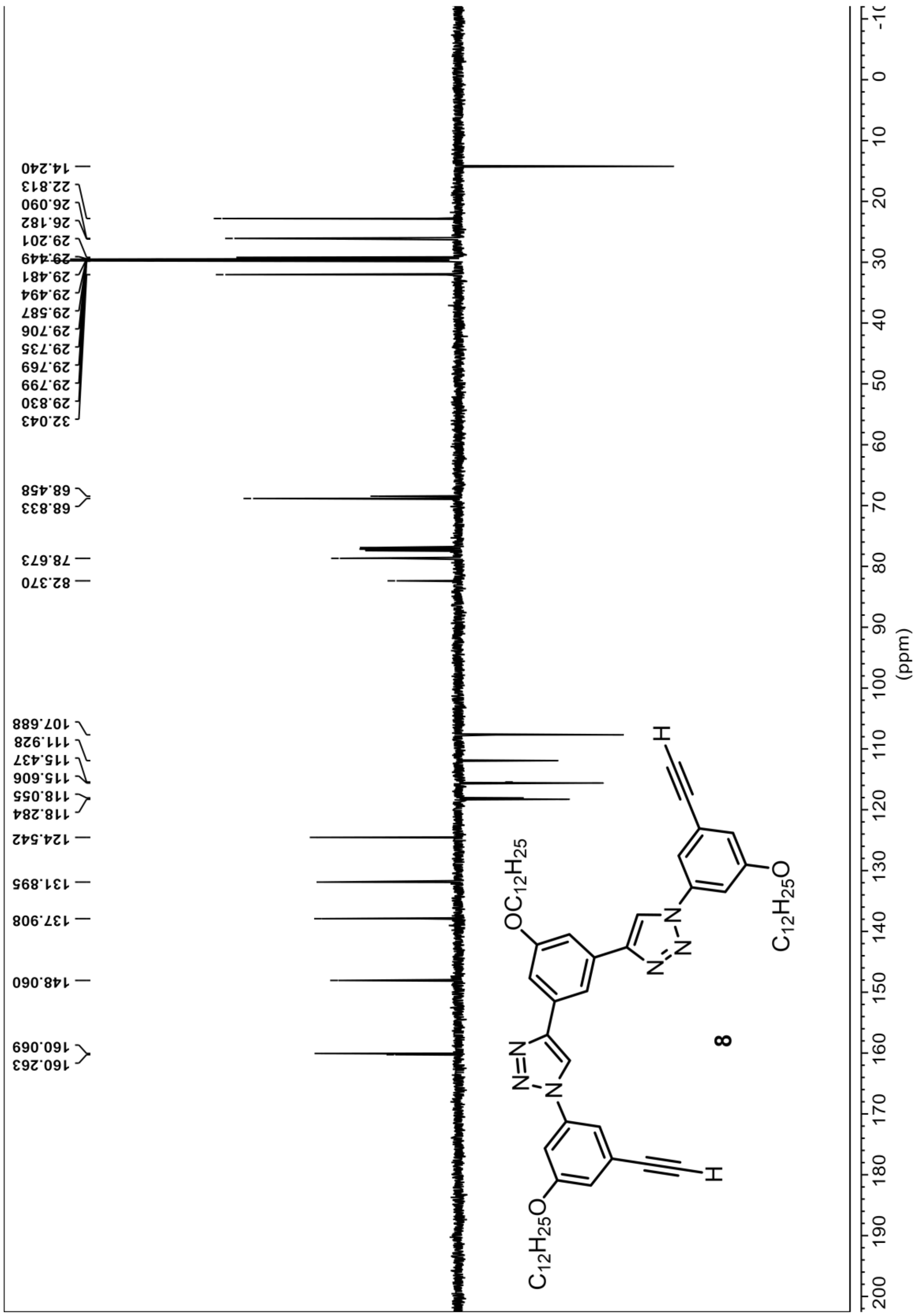

Figure S12. $^{13}C\{^{1}H\}$ APT NMR spectrum of compound **8** in $CDCl_3$ (126 MHz).

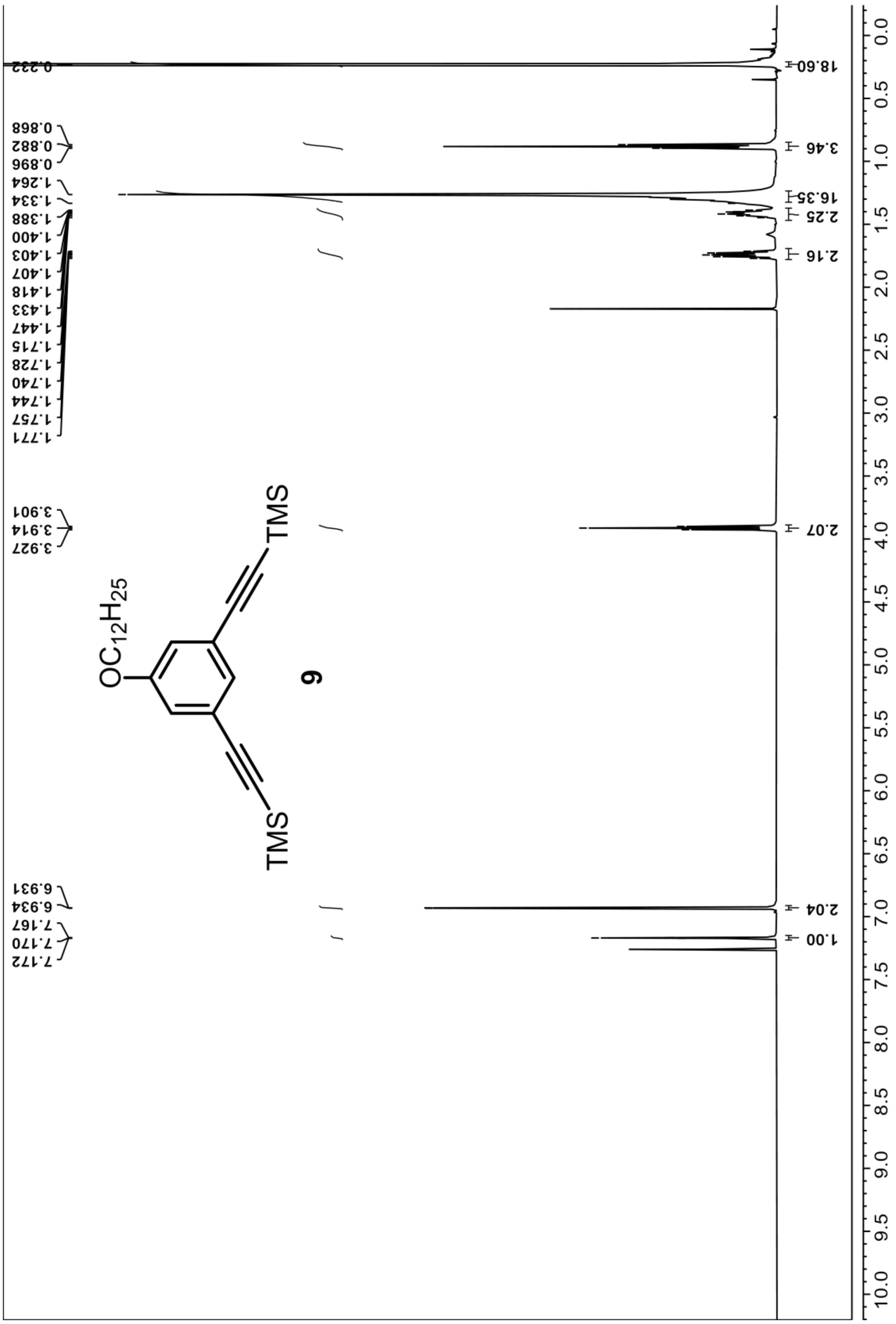

Figure S13. ^{1}H NMR spectrum of compound **9** in $CDCl_3$ (500 MHz).

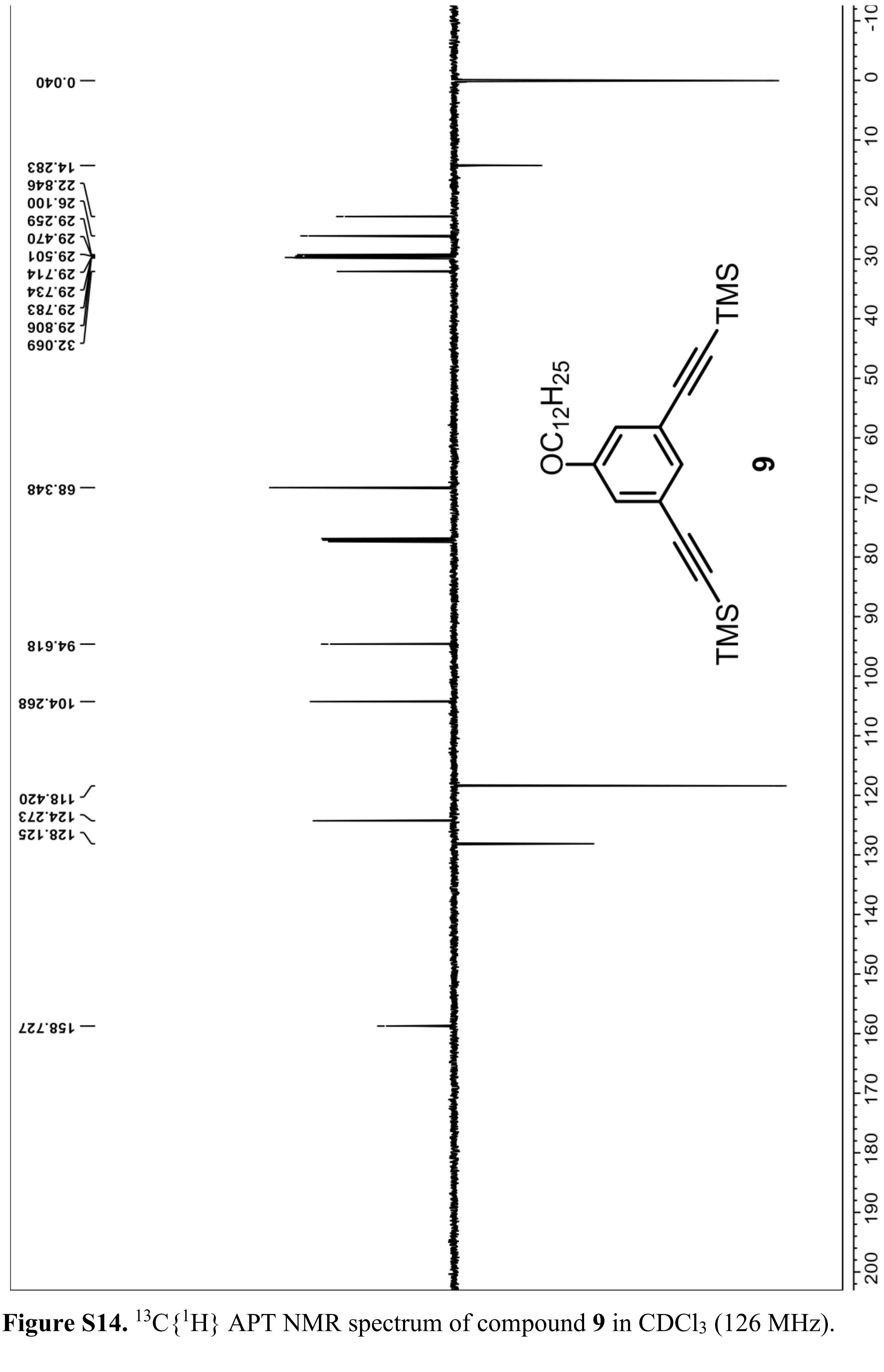

Figure S14. $^{13}C\{^1H\}$ APT NMR spectrum of compound **9** in $CDCl_3$ (126 MHz).

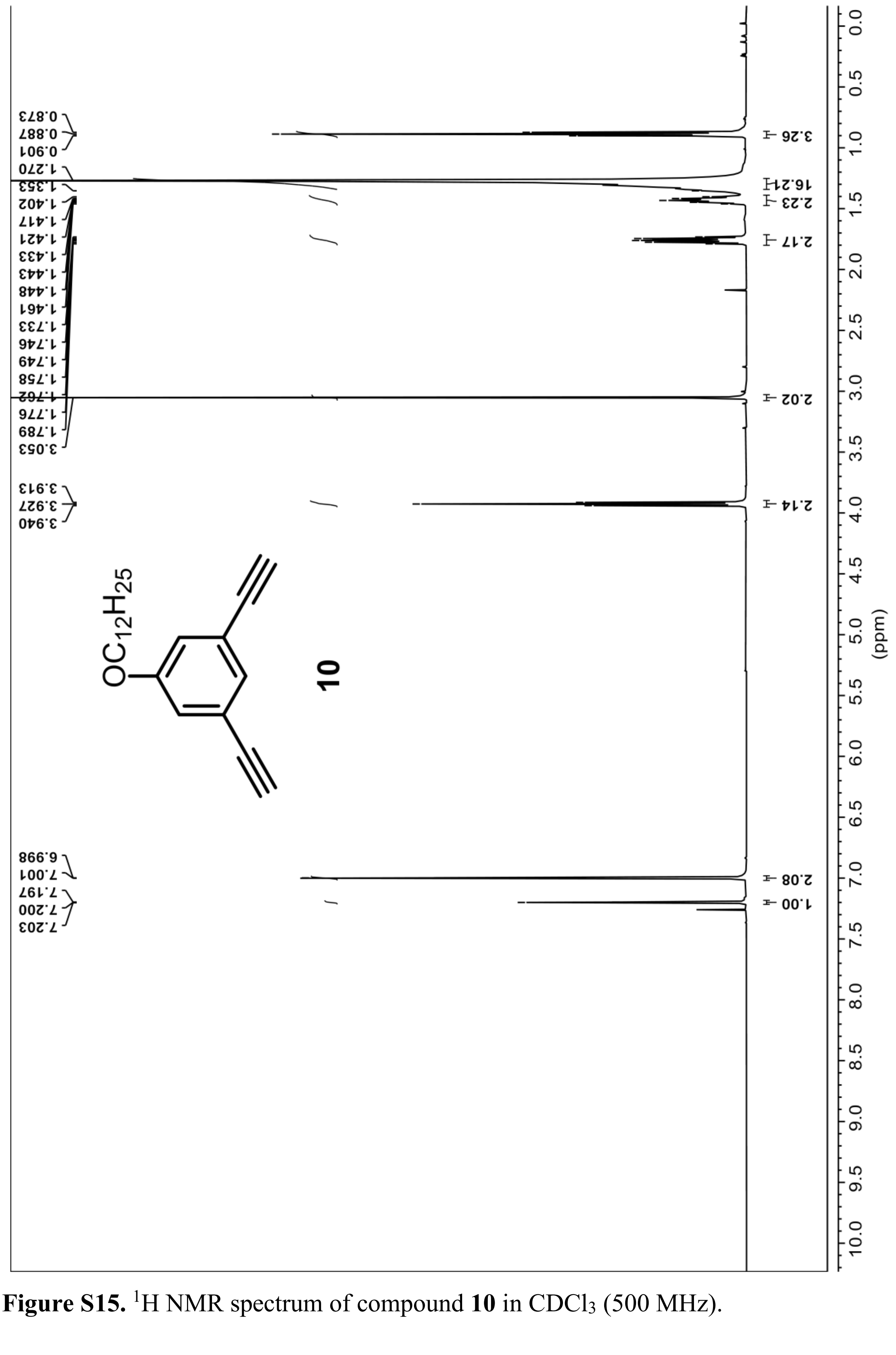

Figure S15. ^{1}H NMR spectrum of compound **10** in $CDCl_3$ (500 MHz).

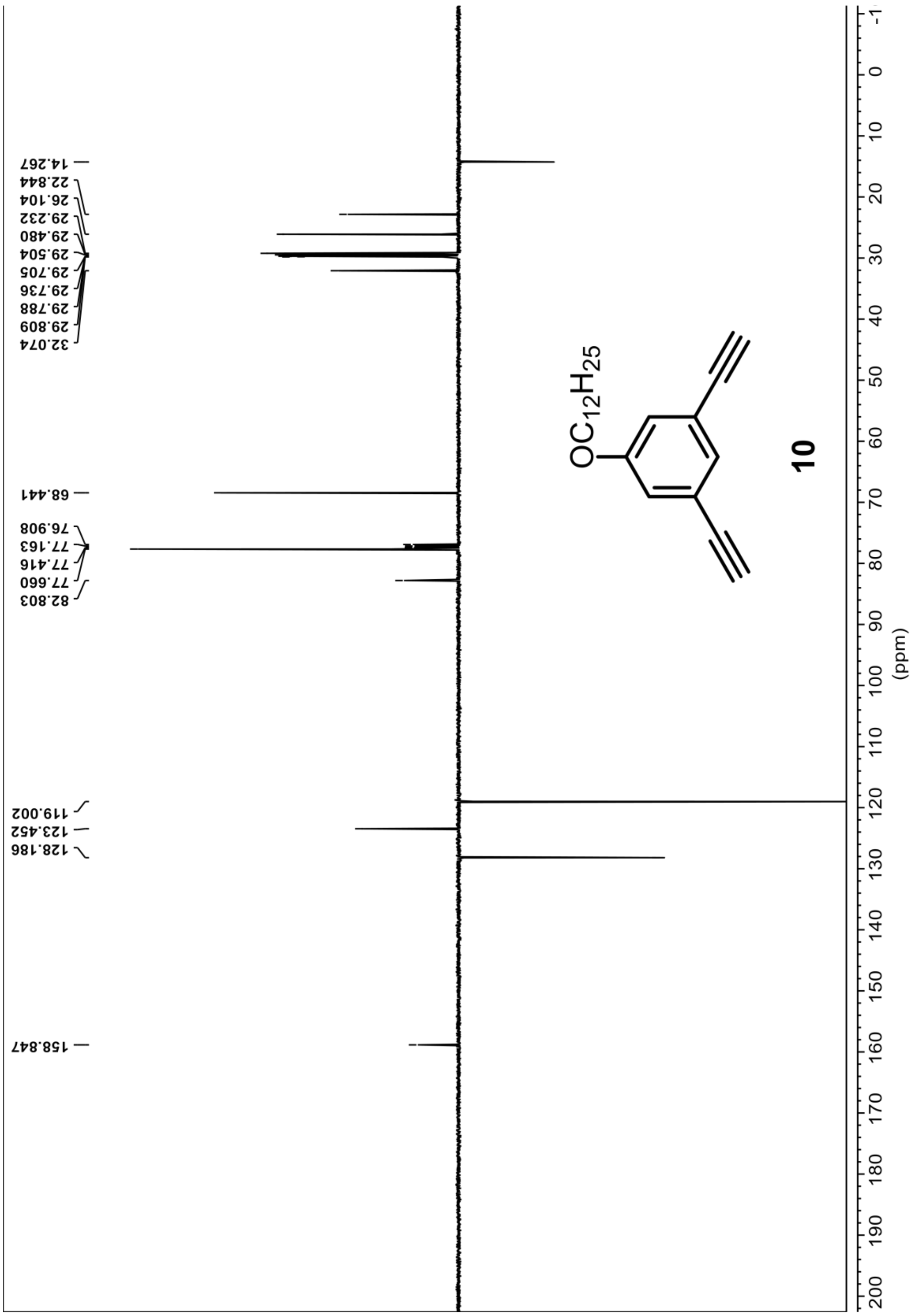

Figure S16. $^{13}C\{^{1}H\}$ APT NMR spectrum of compound **10** in $CDCl_3$ (126 MHz).

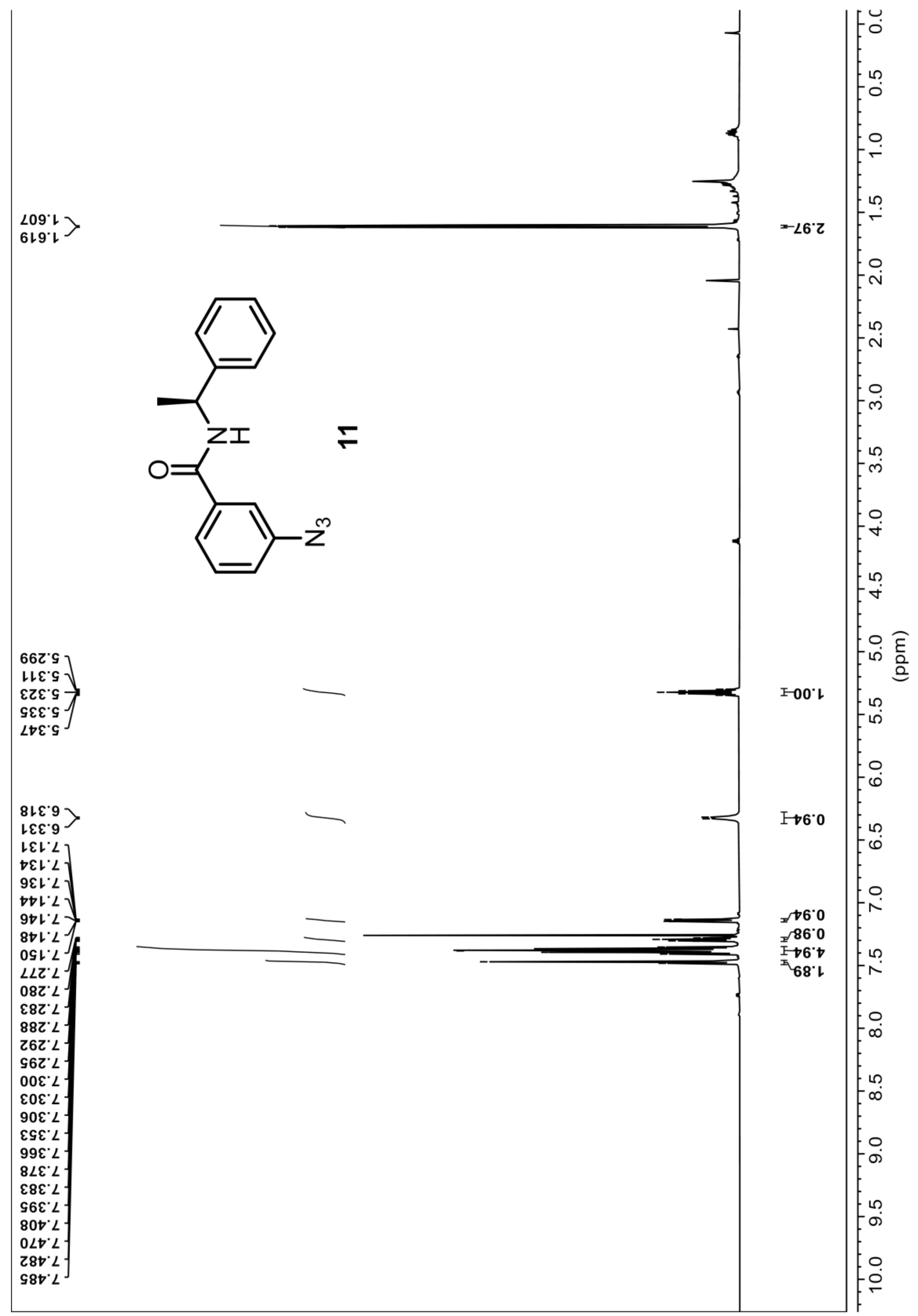

Figure S17. ^{1}H NMR spectrum of compound **11** in $CDCl_3$ (600 MHz).

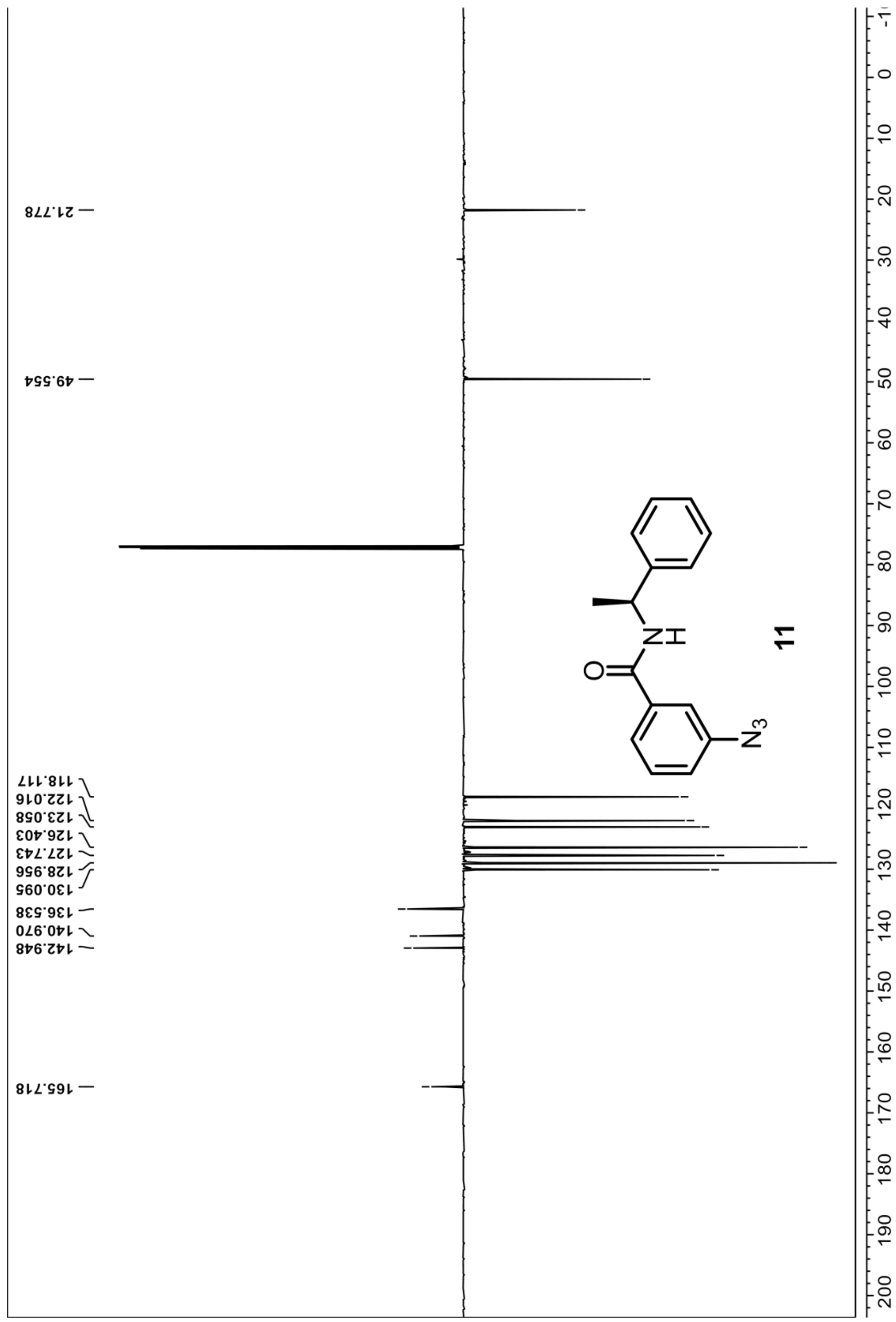

Figure S18. $^{13}C\{^{1}H\}$ APT NMR spectrum of compound **11** in $CDCl_3$ (151 MHz).

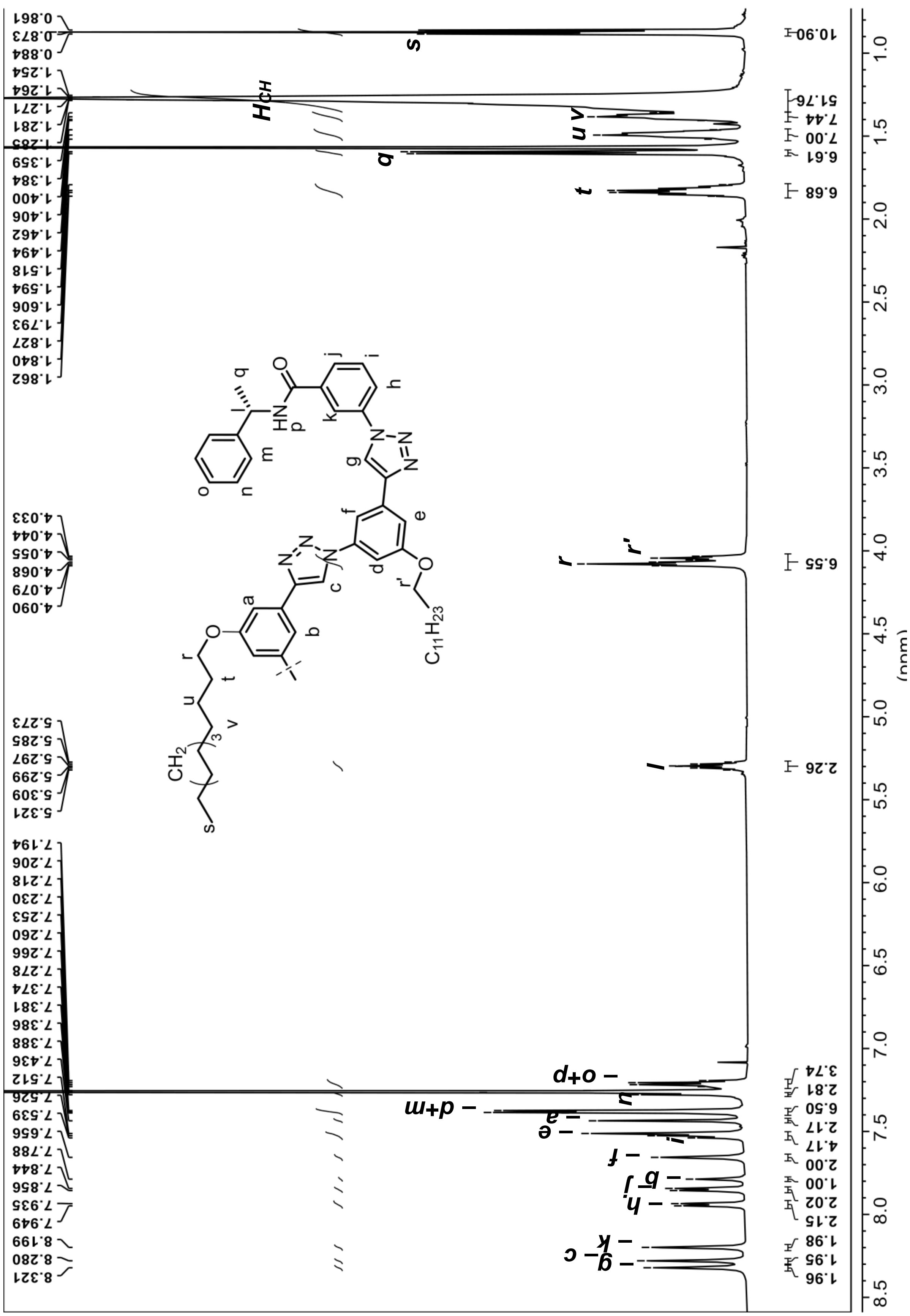

Figure S19. ^{1}H NMR spectrum of compound **1** in $CDCl_3$ (600 MHz).

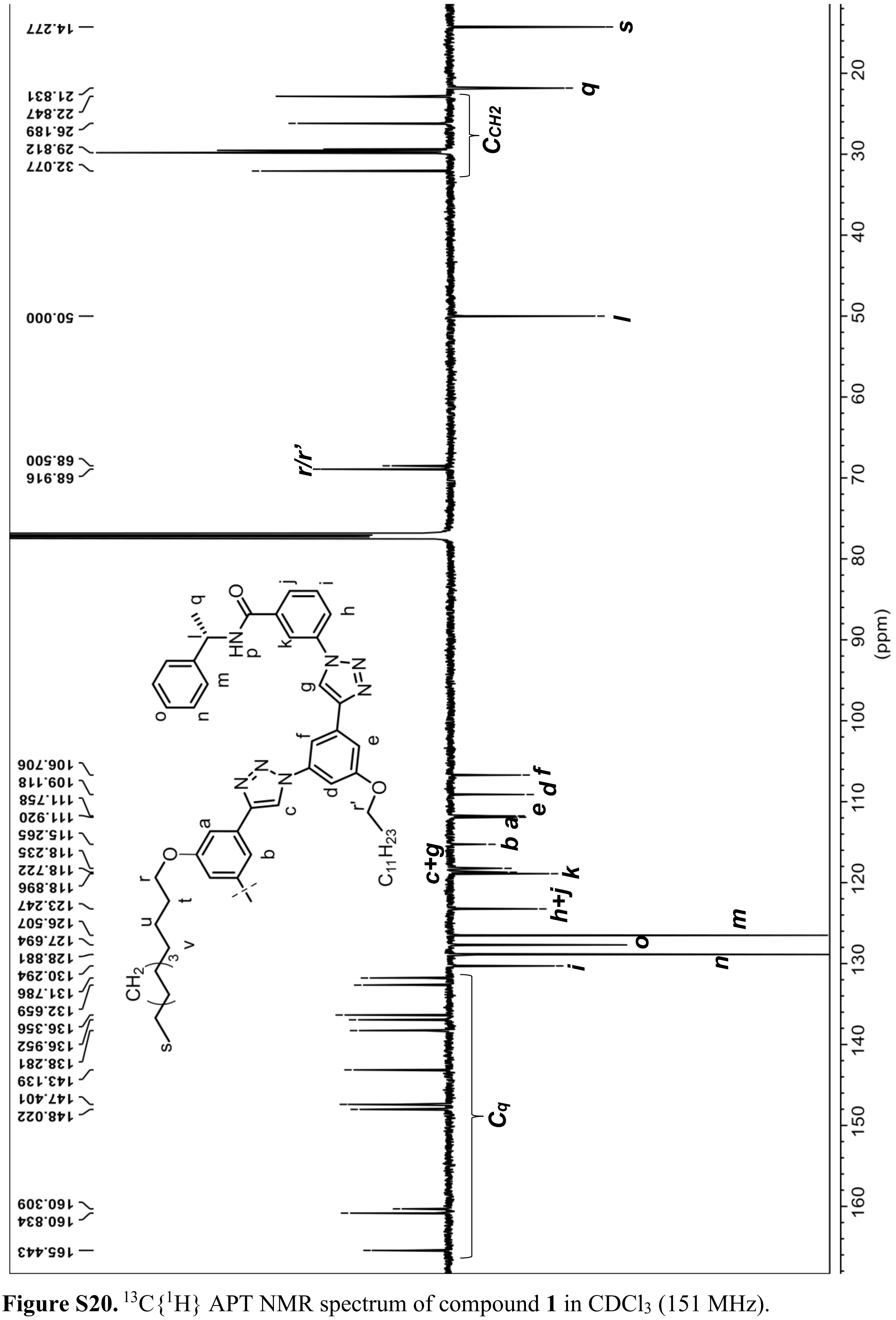

Figure S20. $^{13}C\{^{1}H\}$ APT NMR spectrum of compound **1** in $CDCl_3$ (151 MHz).

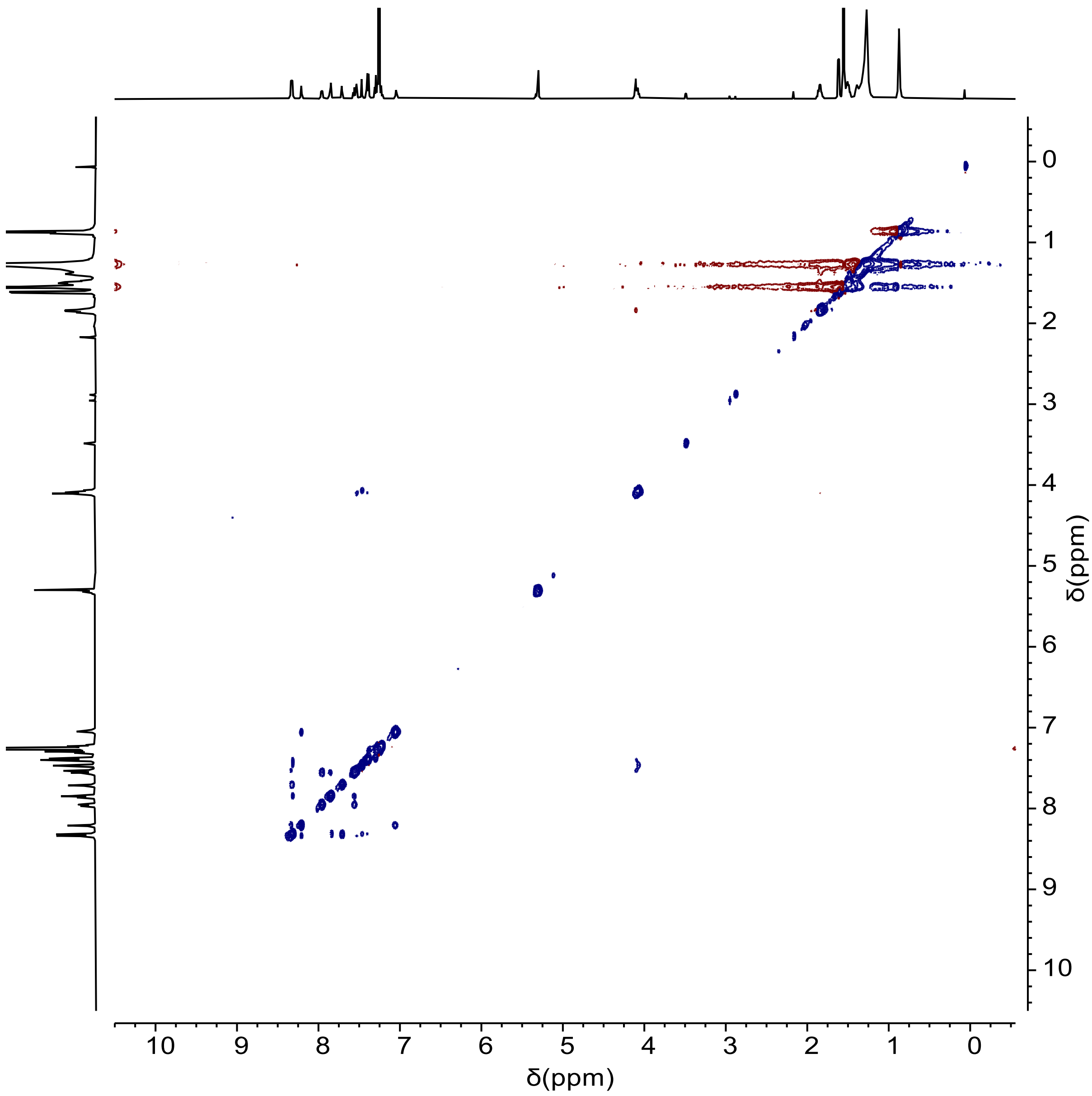

Figure S21. ^{1}H-^{1}H NOESY spectrum of compound of **1** in absence of chloride (600 MHz, $CDCl_3$, 1 mM).

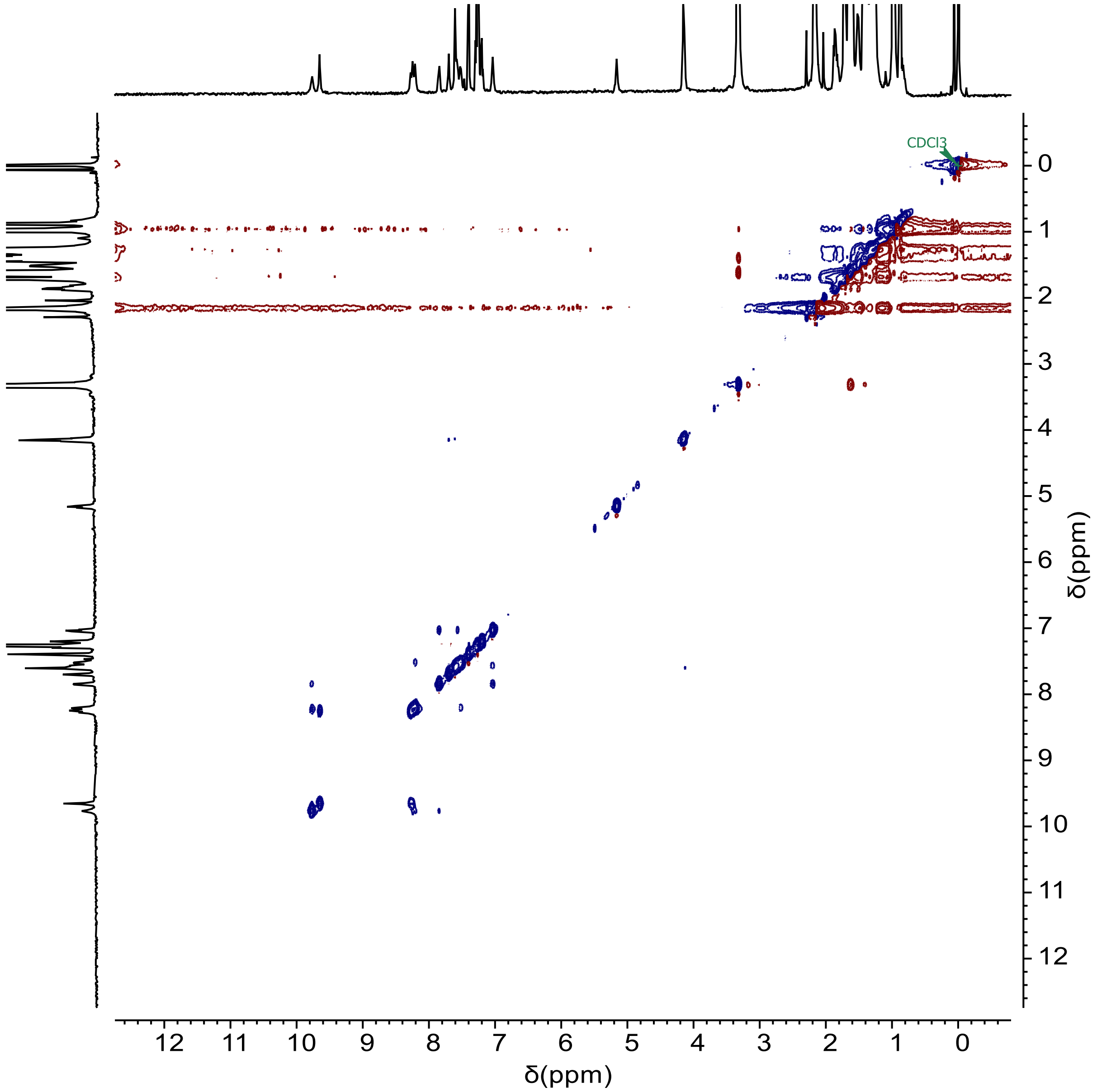

Figure S22. ^{1}H-^{1}H NOESY spectrum of compound **1** (1 mM) with 5.2 eq $[Bu_4N]^+[Cl]^-$ in $CDCl_3$ (600 MHz).

^{1}H NMR titration experiment of (*S*,*S*)-**1** with $[Bu_4N]^+[Cl]^-$

First, a solution of foldamer **1** was prepared in $CDCl_3$ at 1.0 mM. This solution was then used to prepare the $[Bu_4N]^+[Cl]^-$ solution at 10.0 mM, which was added stepwise to 0.5 mL of the foldamer solution. After each addition, a ^{1}H NMR spectrum (500 MHz) was recorded.

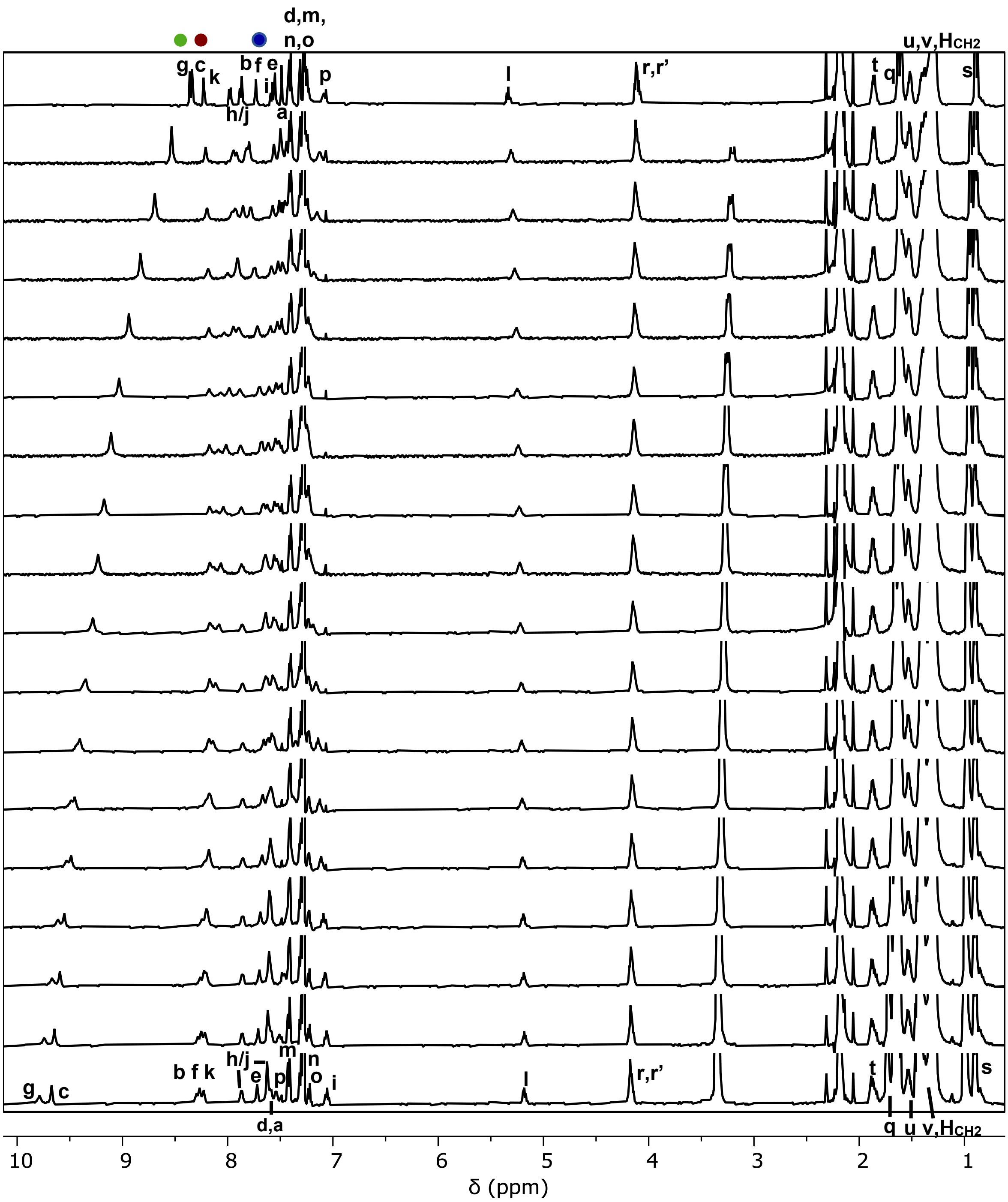

Figure S23. ^{1}H NMR spectral changes (500 MHz, 298 K) of (*S*,*S*)-**1** (1.0 mM in $CDCl_3$) upon incremental addition of $[Bu_4N]^+[Cl]^-$ (from top to bottom: 0.00, 0.21, 0.41, 0.61, 0.79, 0.97, 1.15, 1.32, 1.48, 1.63, 1.93, 2.21, 2.47, 2.72, 3.27, 3.76, 4.55, 5.19 equivalents).

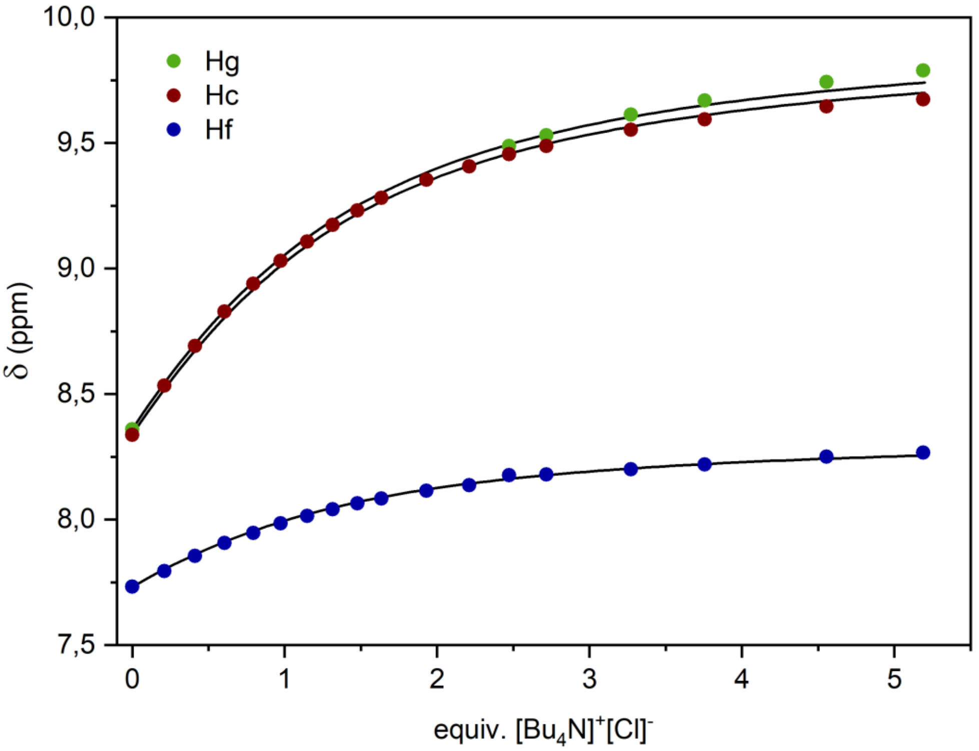

Figure S24. Titration curves for the addition of $[Bu_4N]^+[Cl]^-$ to (*S*,*S*)-**1**. Data fits were obtained using HypNMR by simultaneous analysis of H_c, H_g and H_f.[3] $K_a = 1.31 \times 10^3\ M^{-1}$.

^{1}H NMR titration experiment of (*S*,*S*)-1 with $[Et_4N]^+[Cl]^-$

First, a solution of foldamer **1** was prepared in $CDCl_3$ at 1.0 mM. This solution was then used to prepare the $[Et_4N]^+[Cl]^-$ solution at 22.9 mM, which was added stepwise to 0.5 mL of the foldamer solution. After each addition, a ^{1}H NMR spectrum (500 MHz) was recorded.

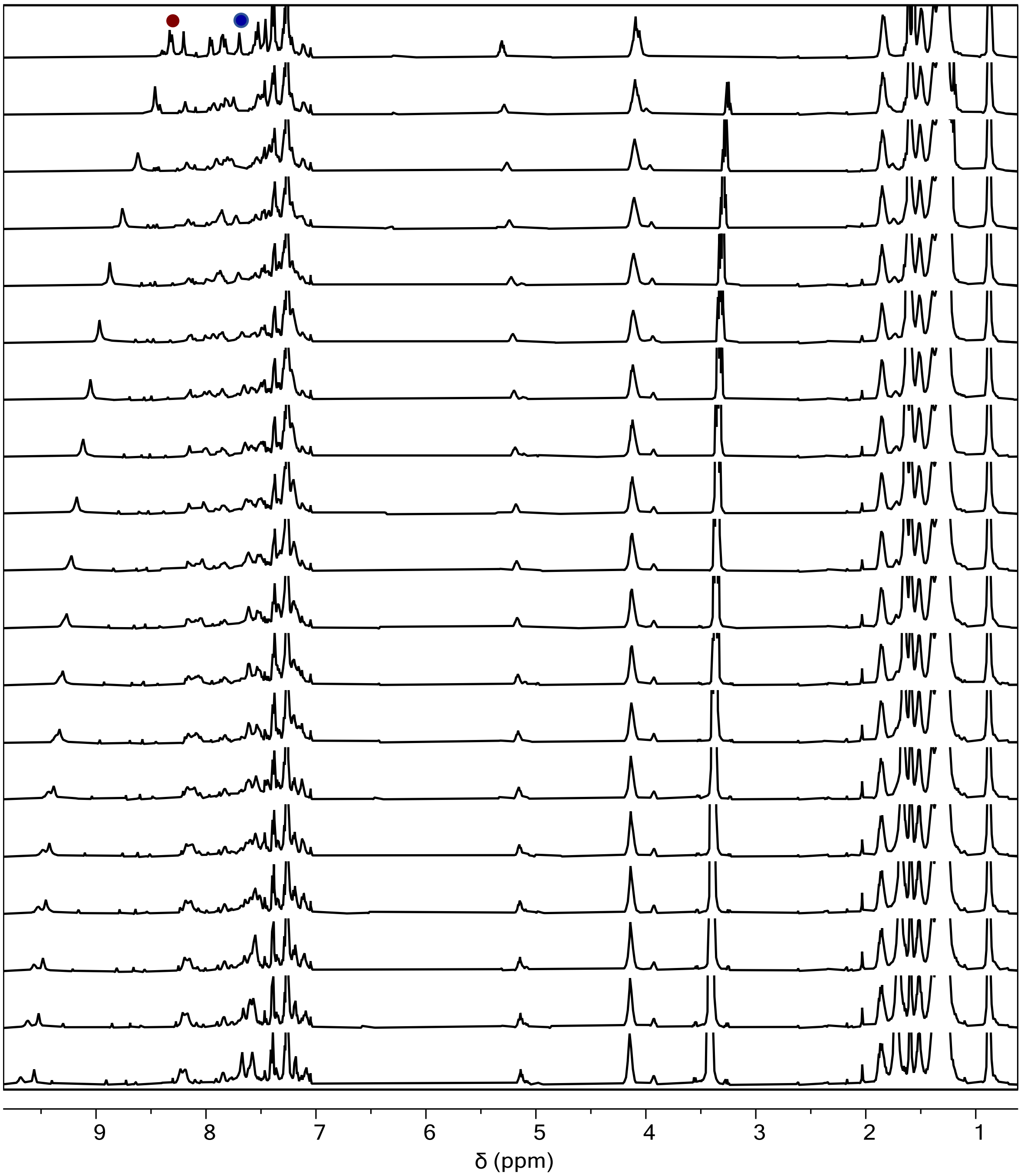

Figure S25. ^{1}H NMR spectral changes (500 MHz, 298 K) of (*S*,*S*)-**1** (1.0 mM in $CDCl_3$) upon incremental addition of $[Et_4N]^+[Cl]^-$ (from top to bottom: 0.00, 0.22, 0.43, 0.65, 0.85, 1.06, 1.25, 1.45, 1.64, 1.83, 2.01, 2.20, 2.37, 2.72, 3.06, 3.38, 3.69, 4.29, 5.11equivalents).

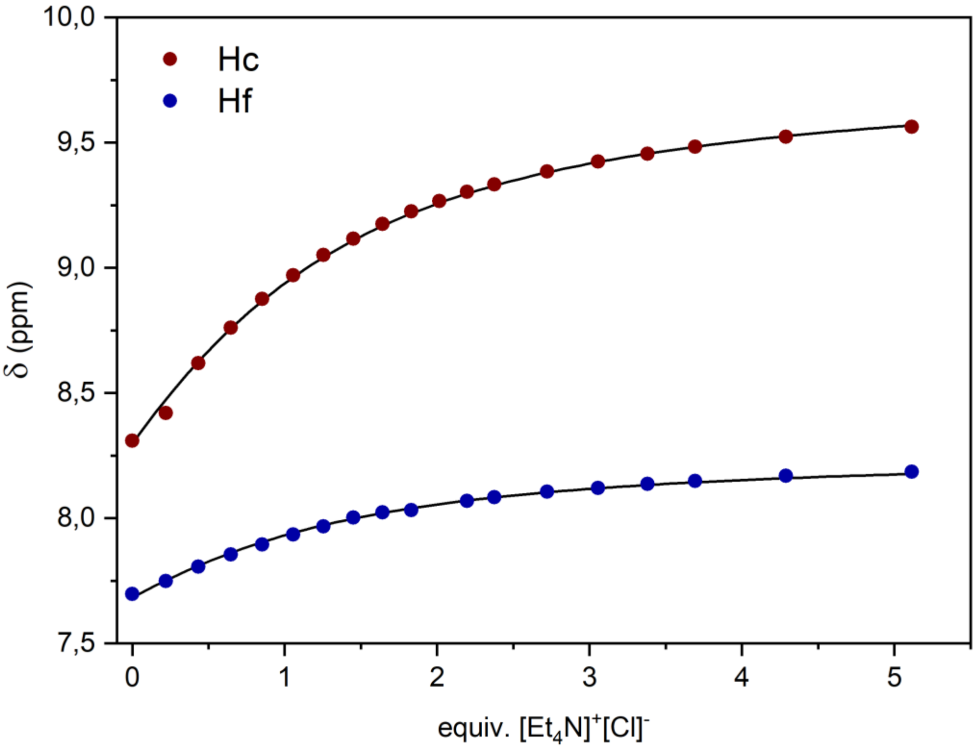

Figure S26. Titration curves for the addition of $[Et_4N]^+[Cl]^-$ to (*S*,*S*)-**1**. Data fits were obtained using HypNMR by simultaneous analysis of H_c and H_f.[3] $K_a = 1.26 \times 10^3$ M^{-1}.

Circular dichroism absorption studies of (*S*,*S*)-1 and (*R*,*R*)-1

Of either enantiomer of foldamer **1**, a 4.0 mM stock solution was prepared in $CHCl_3$ which was diluted using either CH_2Cl_2 or $CHCl_3$ to obtain respective 100 µM foldamer solutions in 2.5vol% $CHCl_3/CH_2Cl_2$ or $CHCl_3$. Dissolving the foldamer in pure CH_2Cl_2 was not possible. To 200 µL of each solution, a solution of $[Bu_4N]^+[Cl]^-$ (10 mM) in CH_2Cl_2 or $CHCl_3$ was added stepwise. After each addition, a CD spectrum was recorded.

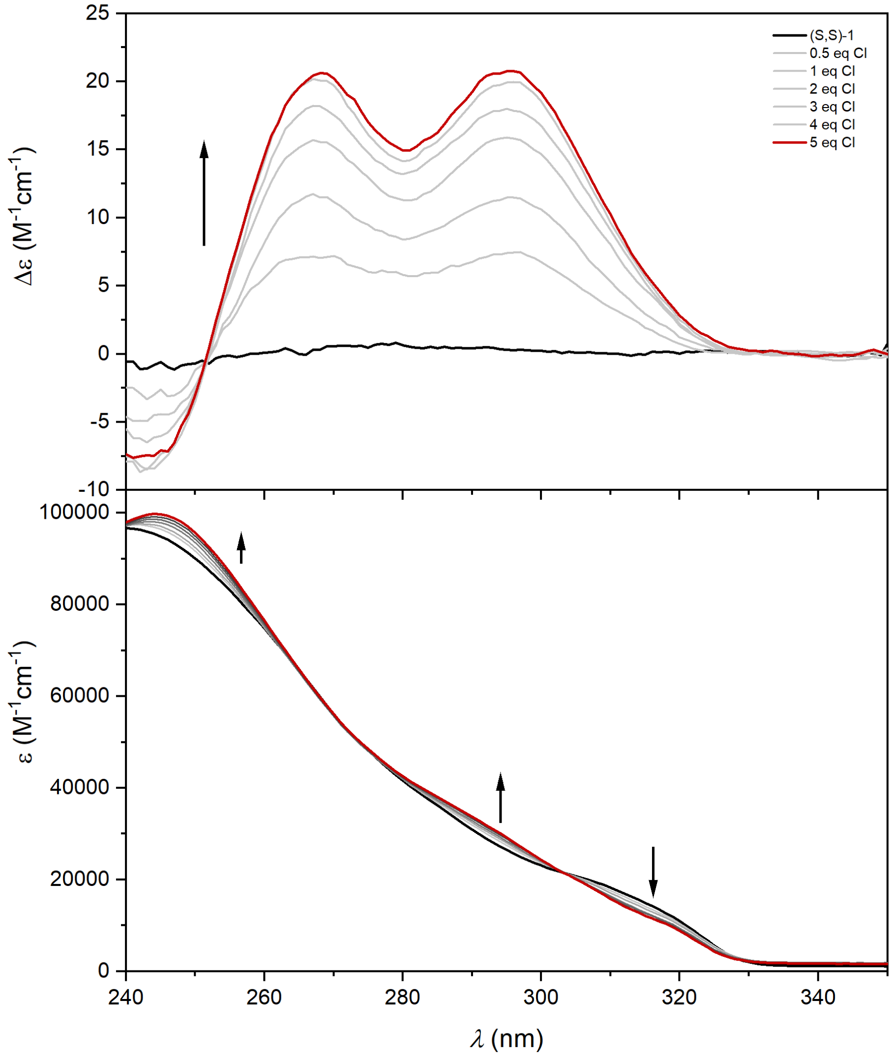

Figure S27. Circular dichroism spectra (top) and concomitant UV absorbance spectra (bottom) of (*S*,*S*)-**1** at 100 µM in 2.5vol% $CHCl_3/CH_2Cl_2$ with incremental addition of $[Bu_4N]^+[Cl]^-$. The clear isosbestic point in the UV-vis spectrum at 303 nm indicates a clean, unimolecular folding process.

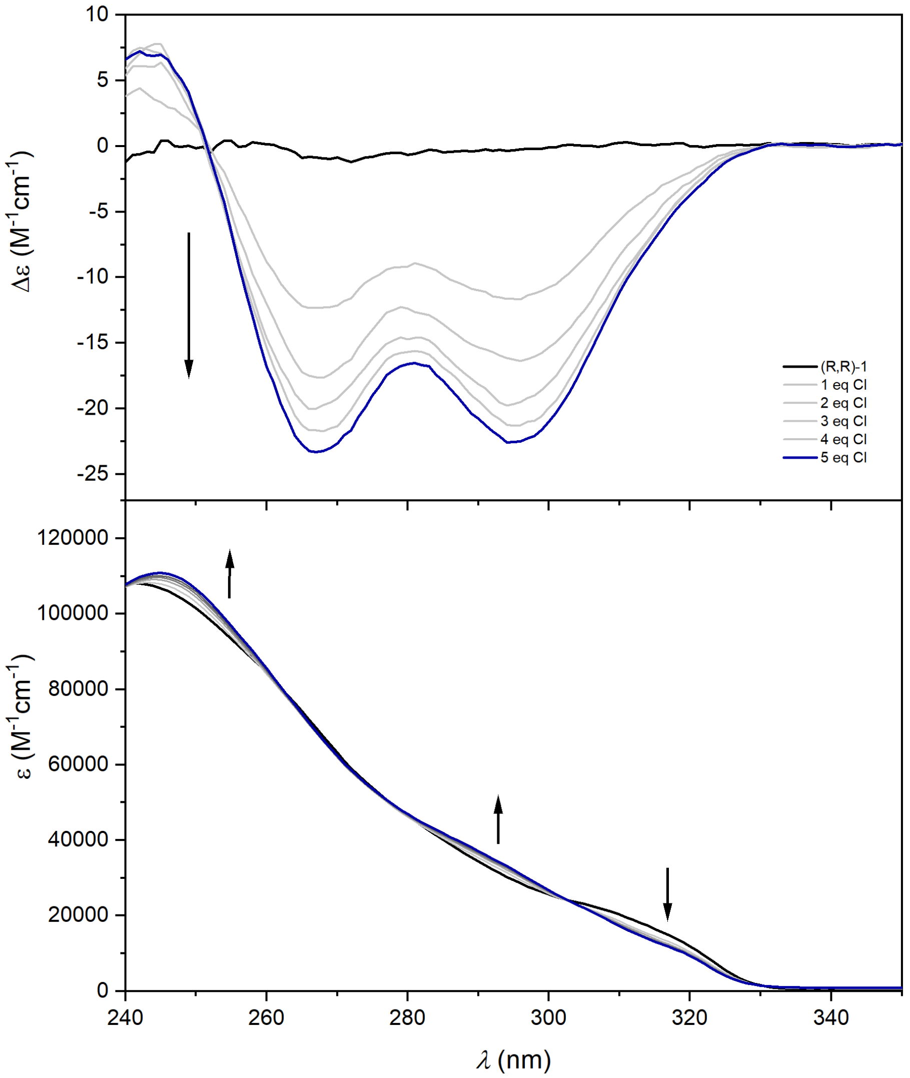

Figure S28. Circular dichroism spectra (top) and concomitant UV absorbance spectra (bottom) of (*R*,*R*)-**1** at 100 μM in 2.5vol% $CHCl_3/CH_2Cl_2$ with incremental addition of $[Bu_4N]^+[Cl]^-$. The clear isosbestic point in the UV-vis spectrum at 303 nm indicates a clean, unimolecular folding process.

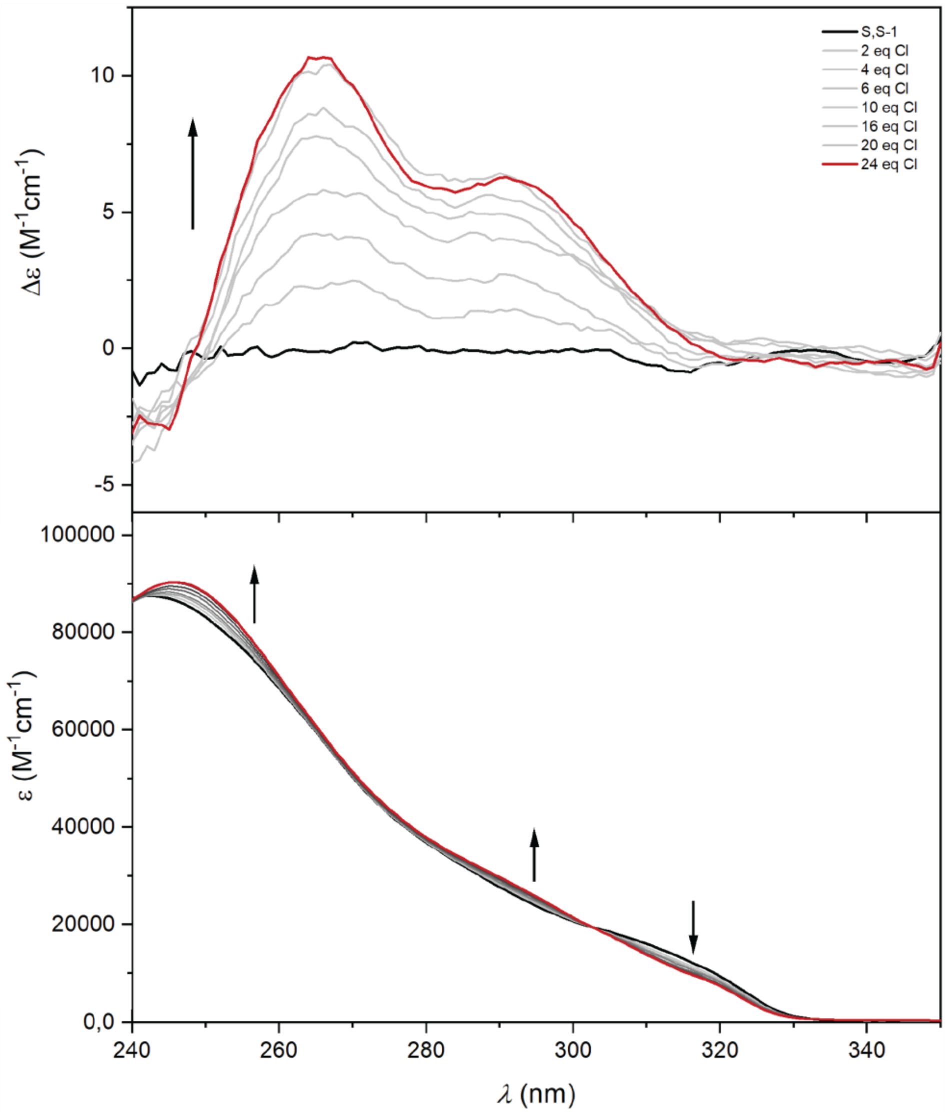

Figure S29. Circular dichroism spectra (top) and concomitant UV absorbance spectra (bottom) of (*S*,*S*)-**1** at 100 μM in $CHCl_3$ with incremental addition of $[Bu_4N]^+[Cl]^-$. The clear isosbestic point in the UV-vis spectrum at 303 nm indicates a clean, unimolecular folding process.

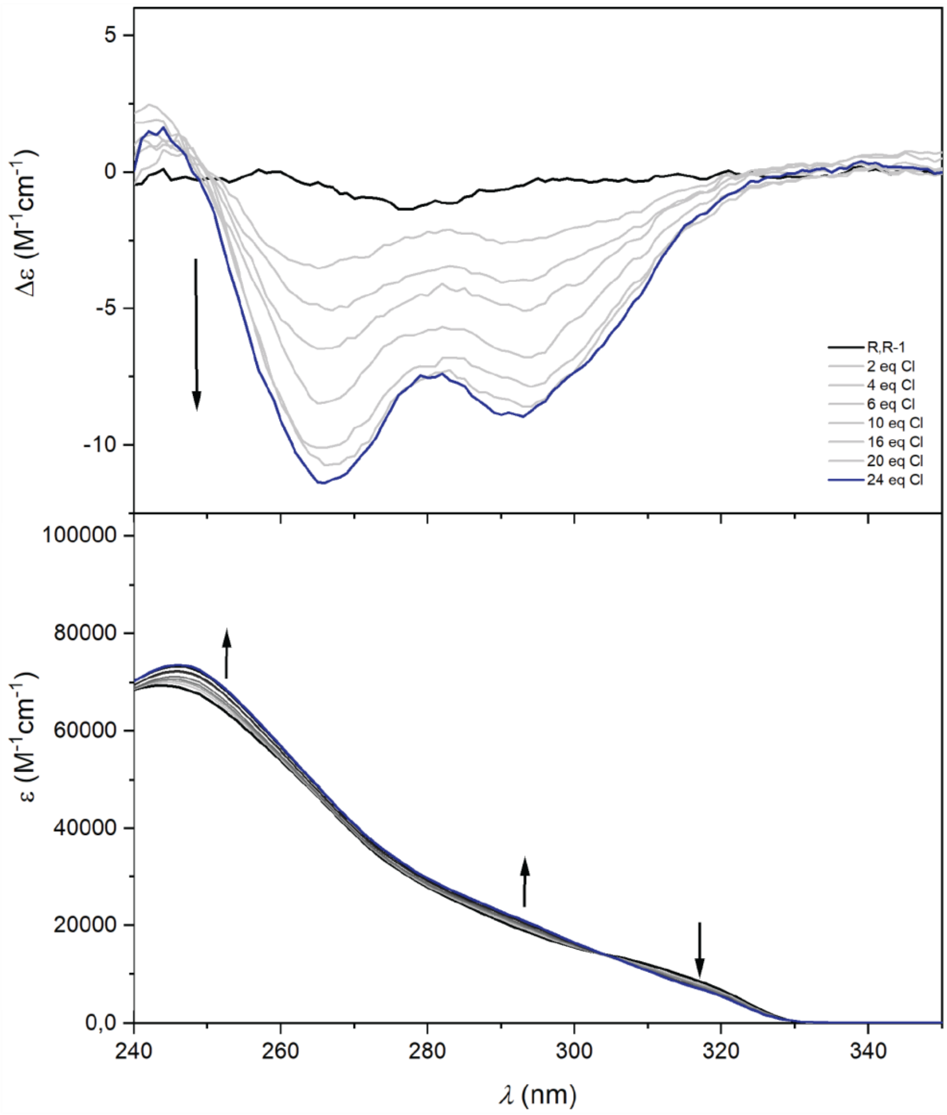

Figure S30. Circular dichroism spectra (top) and concomitant UV absorbance spectra (bottom) of (*R*,*R*)-**1** at 100 µM in $CHCl_3$ with incremental addition of $[Bu_4N]^+[Cl]^-$. The clear isosbestic point in the UV-vis spectrum at 303 nm indicates a clean, unimolecular folding process.

CD titration experiments of (*S*,*S*)-1 with $[Bu_4N]^+[Cl]^-$

A 4.0 mM stock solution of foldamer (*S*,*S*)-**1** was prepared in $CHCl_3$ which was diluted using either CH_2Cl_2 or $CHCl_3$ to obtain respective 100 µM foldamer solutions in 2.5vol% $CHCl_3/CH_2Cl_2$ or $CHCl_3$. Dissolving the foldamer in pure CH_2Cl_2 was not possible. To 200 µL of each solution, a solution of $[Bu_4N]^+[Cl]^-$ in CH_2Cl_2 (2.0 mM) or $CHCl_3$ (20 mM) was added stepwise. After each addition, a CD spectrum was recorded.

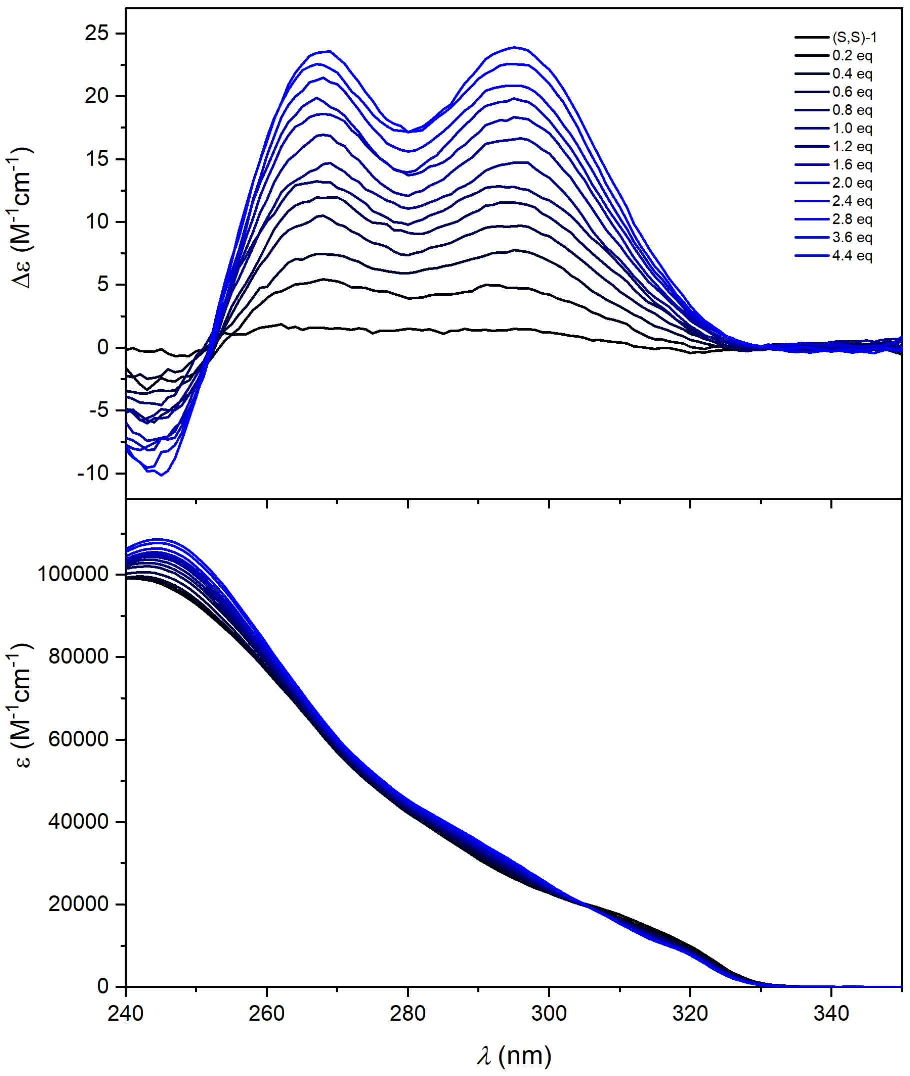

Figure S31. Circular dichroism titration (top) and concomitant UV absorbance spectra (bottom) of (*S*,*S*)-**1** at 100 µM in 2.5vol% $CHCl_3/CH_2Cl_2$ with incremental addition of $[Bu_4N]^+[Cl]^-$.

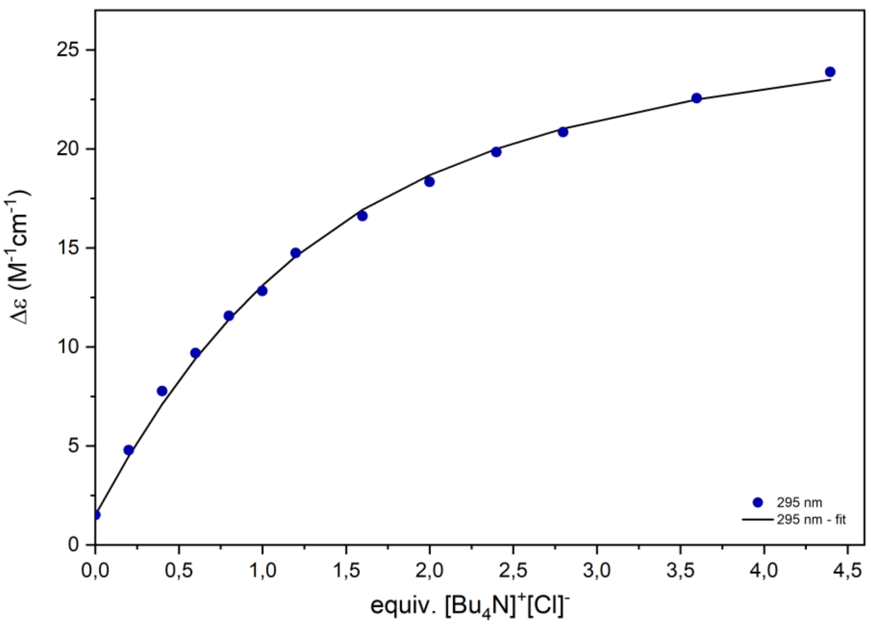

Figure S32. Titration curve for the addition of $[Bu_4N]^+[Cl]^-$ to (*S*,*S*)-**1** (100 μM in 2.5vol% $CHCl_3/CH_2Cl_2$) at 295 nm. The data fit was obtained using Bindfit v5.0.[4] $K_a = 1.30 \pm 0.16 \times 10^4$ M^{-1}.

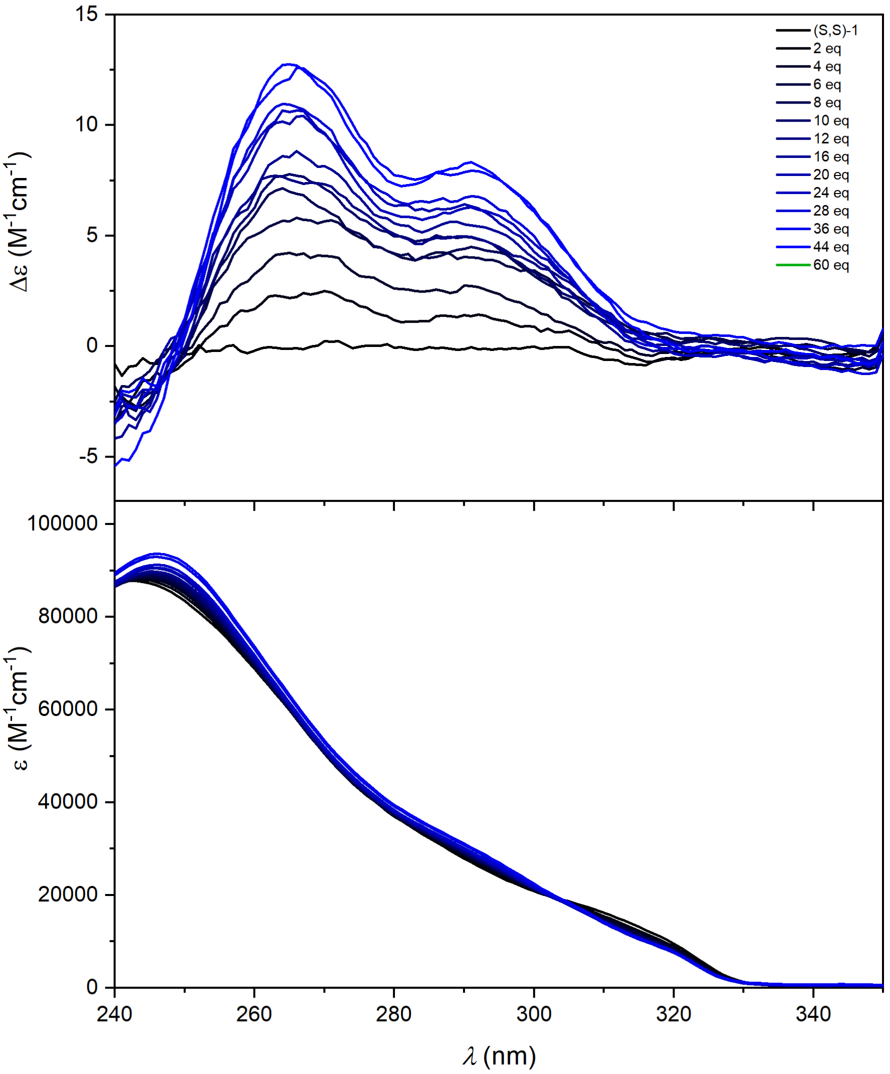

Figure S33. Circular dichroism titration (top) and concomitant UV absorbance spectra (bottom) of (*S*,*S*)-**1** at 100 μM in $CHCl_3$ with incremental addition of $[Bu_4N]^+[Cl]^-$.

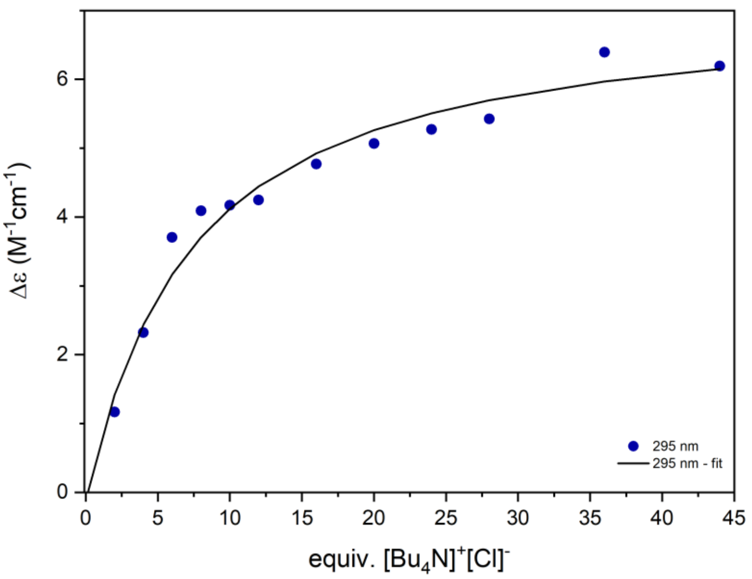

Figure S34. Titration curve for the addition of $[Bu_4N]^+[Cl]^-$ to (*S*,*S*)-**1** (100 μM in $CHCl_3$) at 295 nm. The data fit was obtained using Bindfit v5.0.[4] $K_a = 1.43 \pm 0.10 \times 10^3$ M^{-1}.

UV-vis photostability studies of foldamer 1

A 4.0 mM stock solution of foldamer (*S*,*S*)-**1** was prepared in $CHCl_3$ which was diluted using dry, degassed CH_2Cl_2 to obtain a 100 µM solution in 2.5vol% $CHCl_3/CH_2Cl_2$. Dissolving the foldamer in pure CH_2Cl_2 was not possible. Of this solution, 200 µL in a 1 mm cuvette was irradiated either with 365 nm or 340 nm at 1 cm from the sample. No changes in the absorbance spectra were observed upon irradiation, indicating that the foldamer is stable under these irradiation conditions.

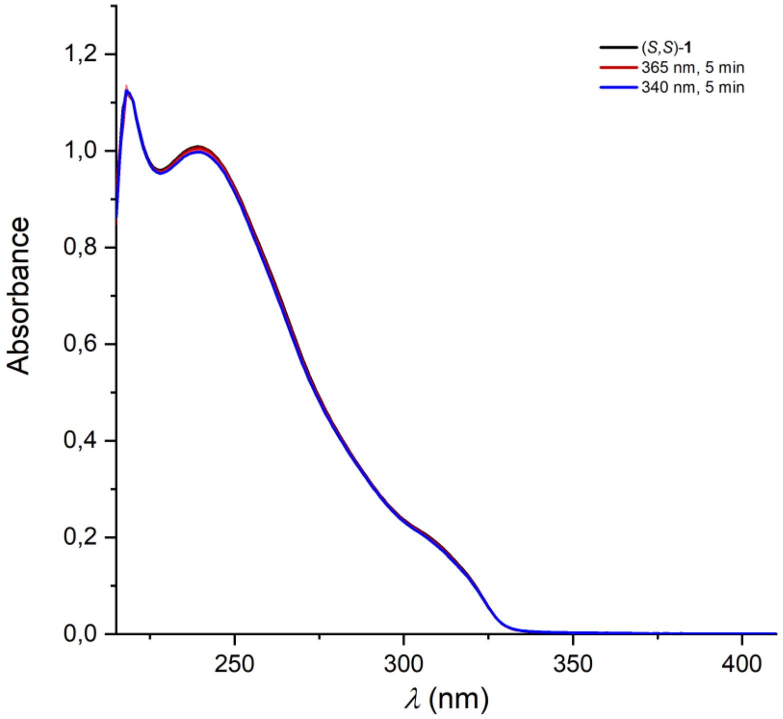

Figure S35. UV-vis spectra of (*S*,*S*)-**1** before and after irradiation with 365 nm (5 min) and 340 nm (5 min).

UV-vis photoisomerization studies of receptor 2

A 1 mM solution of receptor (*Z*)-**2** was prepared in dry, degassed CH_2Cl_2. 200 µL solution was placed in a 1 mm cuvette. Irradiation was performed using either 365 nm or 340 nm at 1 cm from the sample. UV-vis spectroscopy was measured after each irradiation iteration. Irradiation was stopped when no more changes in absorption were observed.

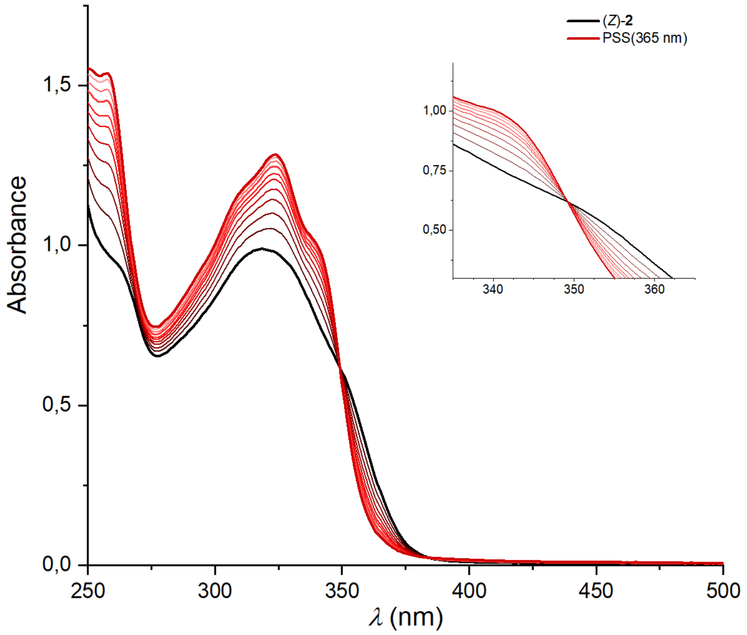

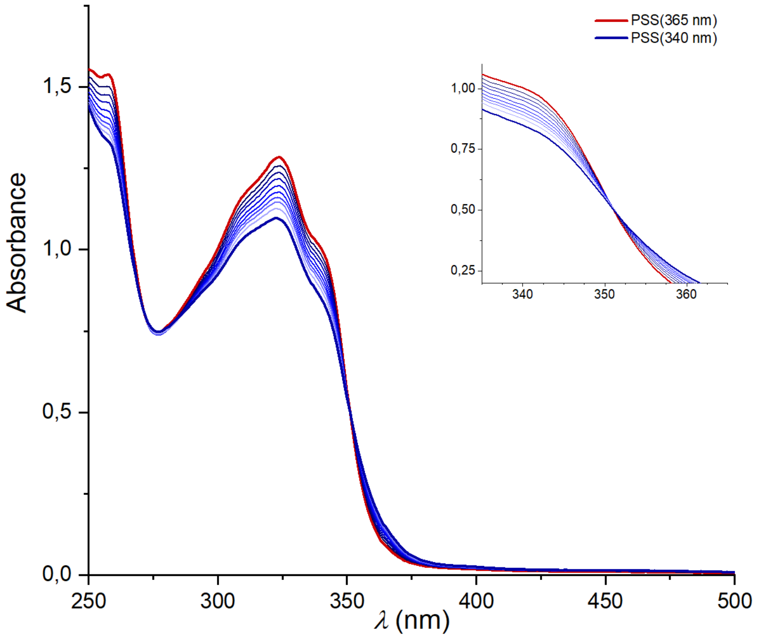

Figure S36. UV-vis spectra of (*Z*)-**2** before and after irradiation with 365 nm (top) and 340 nm (bottom). PSS_{365nm} is reached after 70 s and subsequent PSS_{340nm} after 180 s. The insert shows an amplification of the isosbestic points.

^{1}H NMR photoisomerization studies of receptor 2

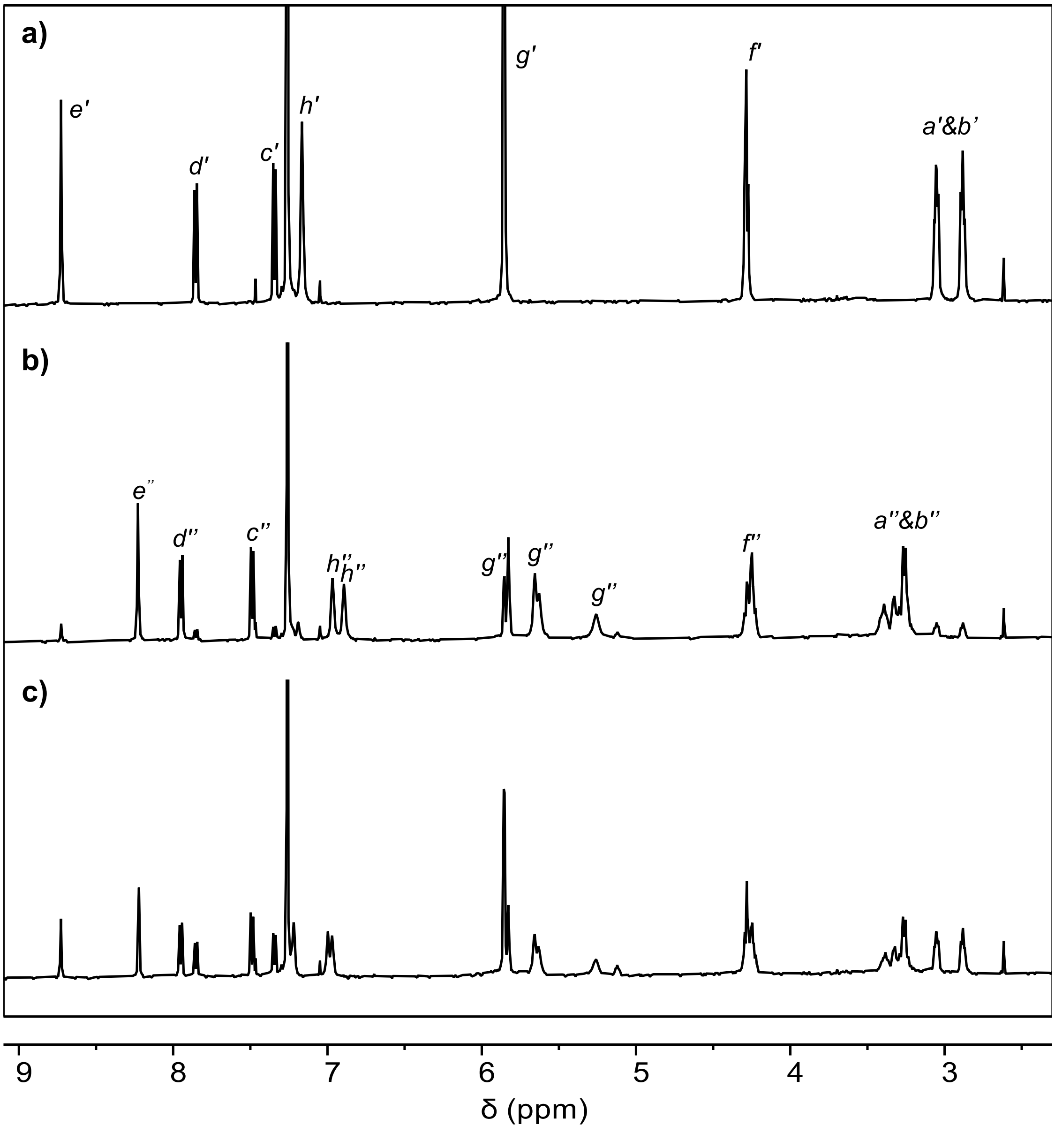

Figure S37. ^{1}H NMR (500 MHz) spectra of a) (*Z*)-**2** (1.0 mM in dry, Ar-degassed $CDCl_3$); b) after irradiation for 75 s with 365 nm, (*E*)/(*Z*) 81:19; c) after irradiation for 145 s with 340 nm, (*E*)/(*Z*) 58:42. The *E*/*Z*-ratio was determined through relative integration of all ^{1}H NMR proton signals.

^{1}H NMR titration experiments of (*Z*)-2 with $[Bu_4N]^+[Cl]^-$

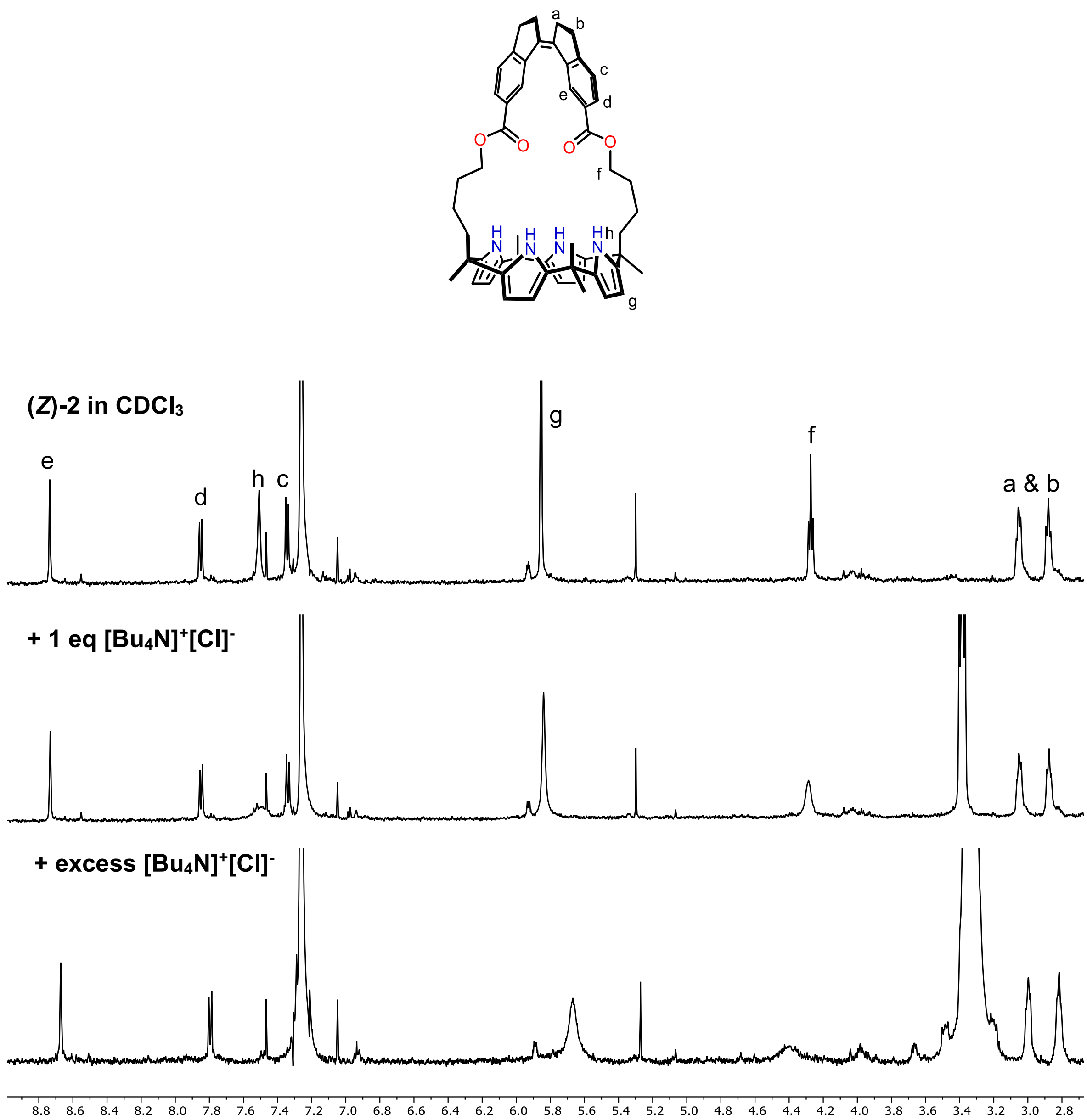

Figure S38. ^{1}H NMR (500 MHz) spectra of (*Z*)-**2** at 1.0 mM in $CDCl_3$ upon addition of $[Bu_4N]^+[Cl]^-$. Virtually no spectral changes are observed indicating no to very weak binding of Cl^- in the receptor.

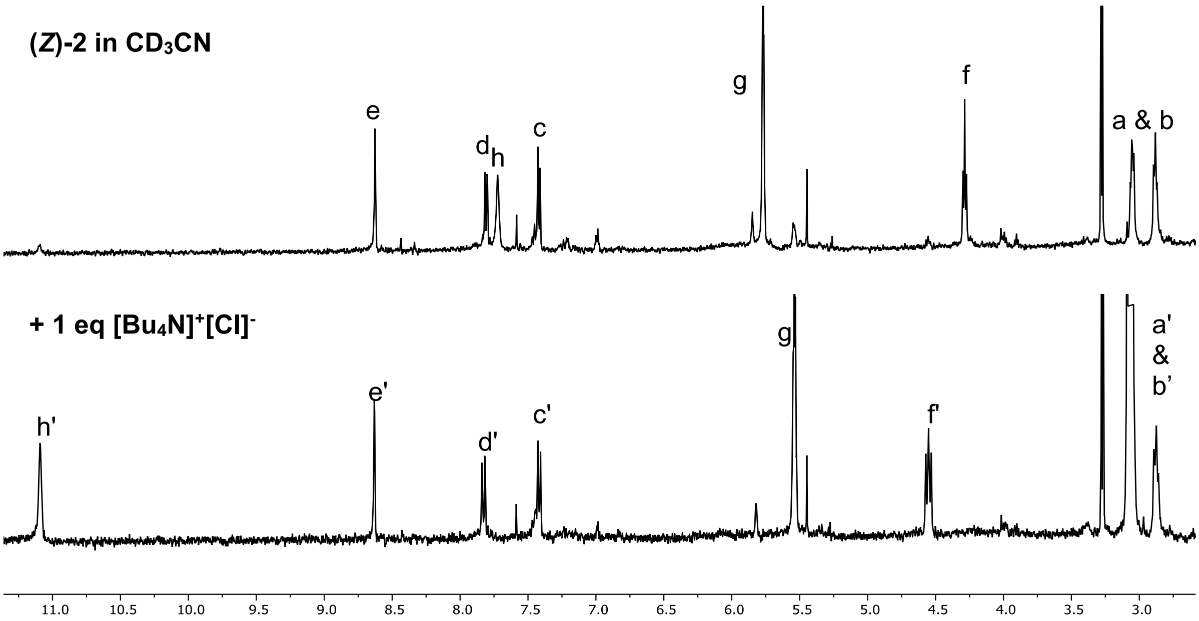

Figure S39. ^{1}H NMR (500 MHz) spectra of (*Z*)-**2** at 1.0 mM in CD_3CN upon addition of $[Bu_4N]^+[Cl]^-$. A new set of signals is observed upon chloride addition (indicated by hyphen) indicating saturation and thus very strong binding of the anion to the receptor.

^{1}H NMR titration experiments of (*Z*)-2 with $[Et_4N]^+[Cl]^-$

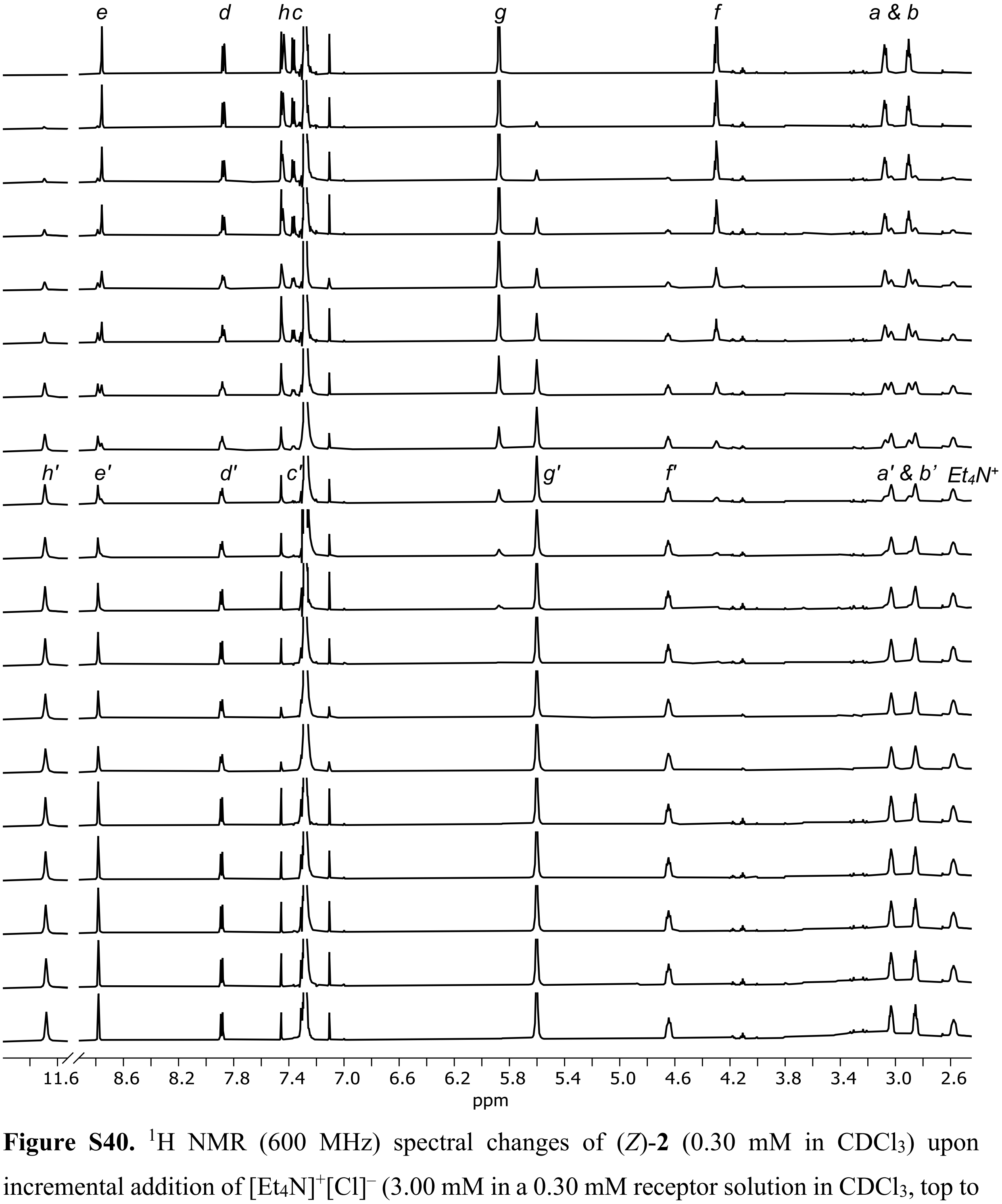

Figure S40. ^{1}H NMR (600 MHz) spectral changes of (*Z*)-**2** (0.30 mM in $CDCl_3$) upon incremental addition of $[Et_4N]^+[Cl]^-$ (3.00 mM in a 0.30 mM receptor solution in $CDCl_3$, top to bottom: 0.00, 0.10, 0.20, 0.29, 0.38, 0.48, 0.65, 0.83, 0.99, 1.15, 1.30, 1.52, 1.80, 2.06, 2.31, 2.54, 2.80, 3.29, 3.71 eq). The free (x) and bound (x’) receptor are in slow exchange causing two separate signals for the two species instead of a gradual change in shift. Signal c’ is under the $CHCl_3$ signal.

From the change in integral of each proton signal, a ratio between bound and unbound receptor was determined. From this ratio, a K_a value from each proton can be calculated using Formula S1. To yield the most accurate results, only those ratios in the linear part of the binding curve, between 20% and 80%, were used.[5] All obtained K_a values were then averaged to obtain the overall K_a value with errors determined by standard deviation; $K_a(Z) = 2.75 \pm 1.26 \times 10^4$ M^{-1}.

Formula S1 Equation to determine K_a for a slow-exchanging system

$$K_a = \frac{[HG]}{[H]_f[G]_f} = \frac{x_{bound}}{x_{unbound}([G]_{total} - x_{bound}[H]_{total})}$$

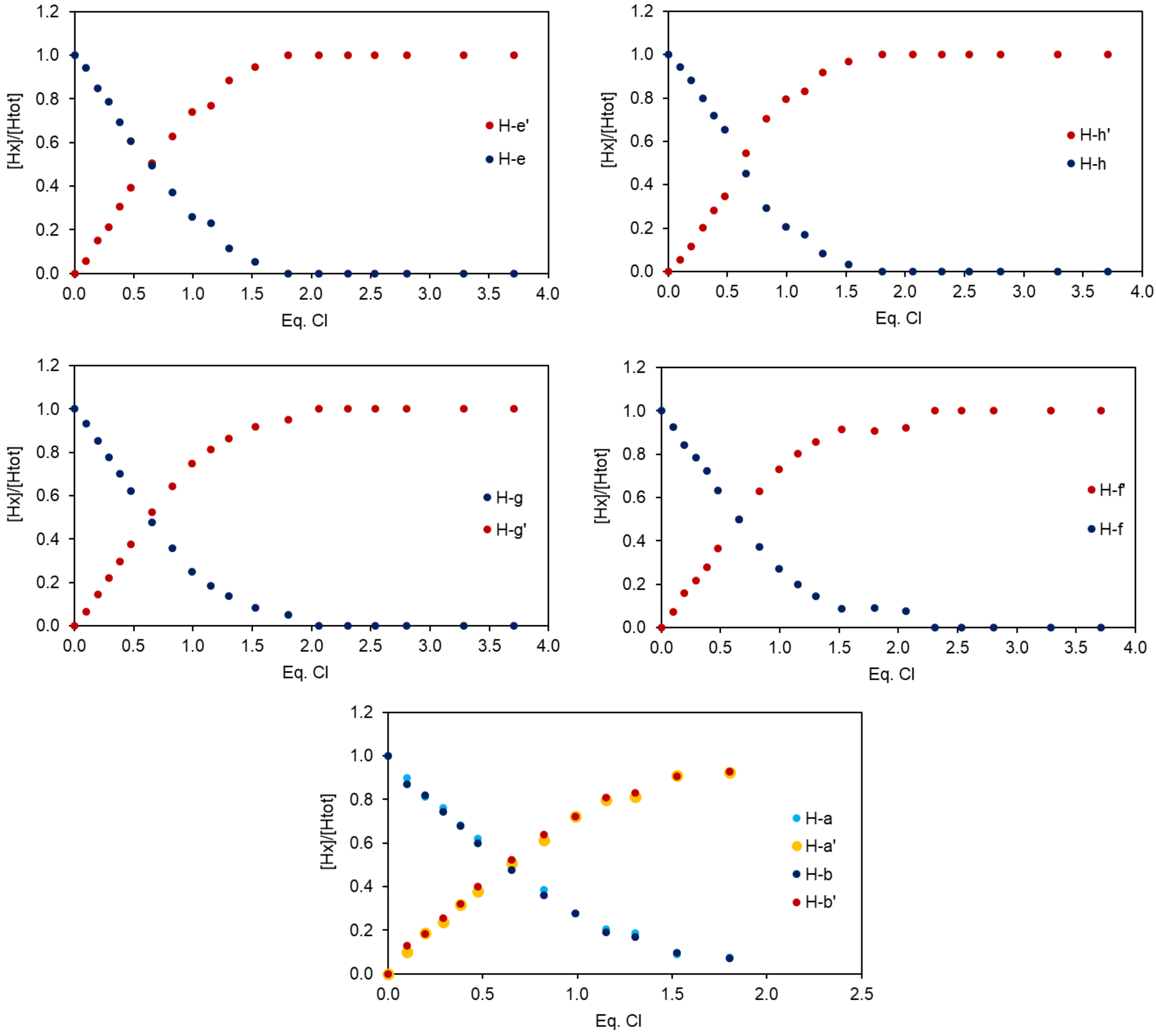

Figure S41. Ratio of the proton signals of bound and unbound species as determined by integral relative to the total host concentration, against equivalents of $[Et_4N]^+[Cl]^-$ added to the system.

^{1}H NMR titration experiments of (*E*)-2 with $[Et_4N]^+[Cl]^-$

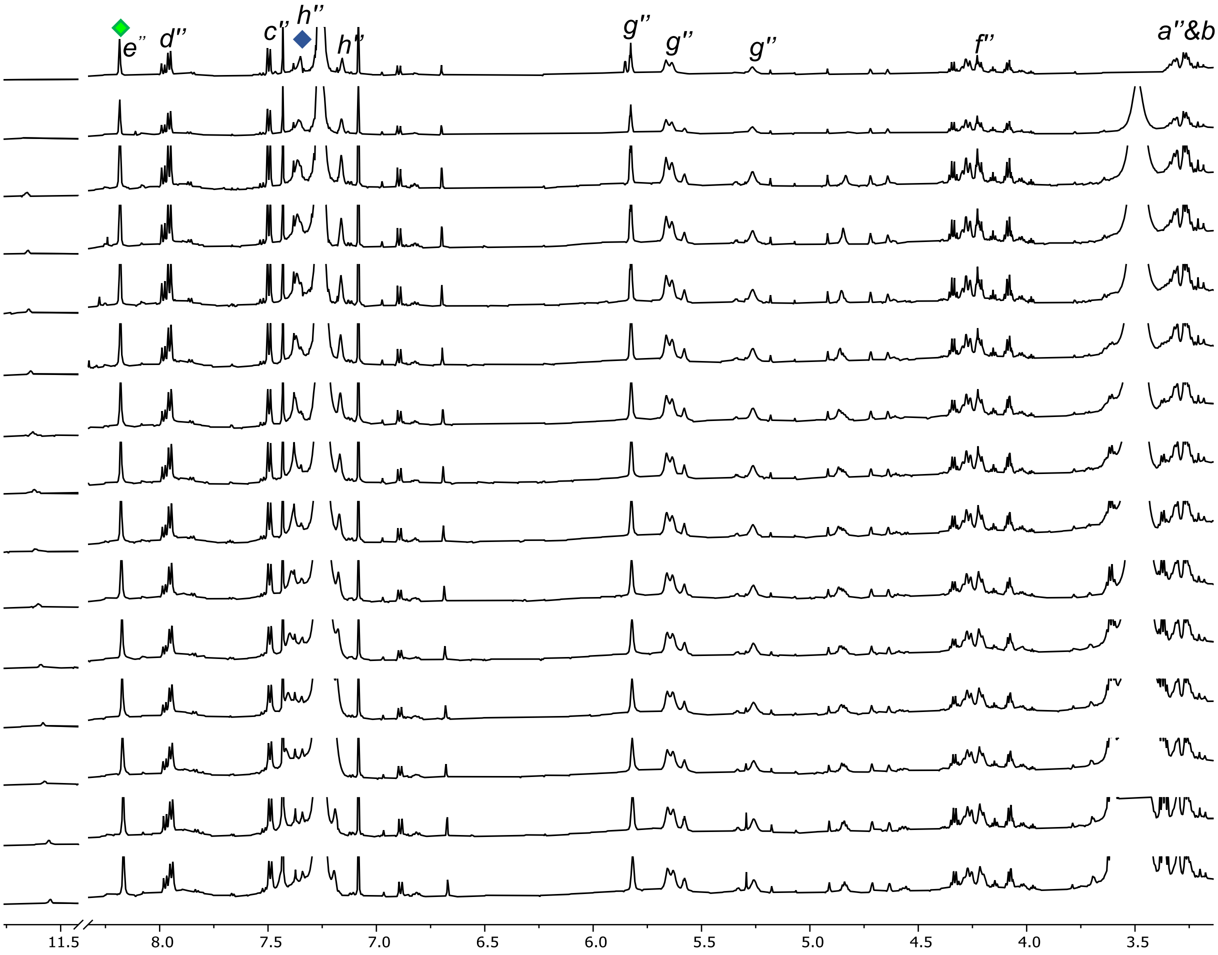

Figure S42. ^{1}H NMR (500 MHz, 298 K) spectral changes of (*E*)-**2** (0.3 mM in $CDCl_3$) upon incremental addition of a $[Et_4N]^+[Cl]^-$ solution (43 mM $[Et_4N]^+[Cl]^-$ and 0.3 mM (*E*)-**2** in $CDCl_3$, top to bottom: 0.00, 1.4, 2.8, 4.1, 5.5, 8.1, 10.5, 12.9, 15.3, 19.6, 23.7, 27.5, 31.1, 39.2, 42.8 eq). (*E*)-**2** was obtained by irradiation of a solution of (*Z*)-**2** with 365 nm for 150 sec. Signals for (*E*)-**2** are assigned with (x'').

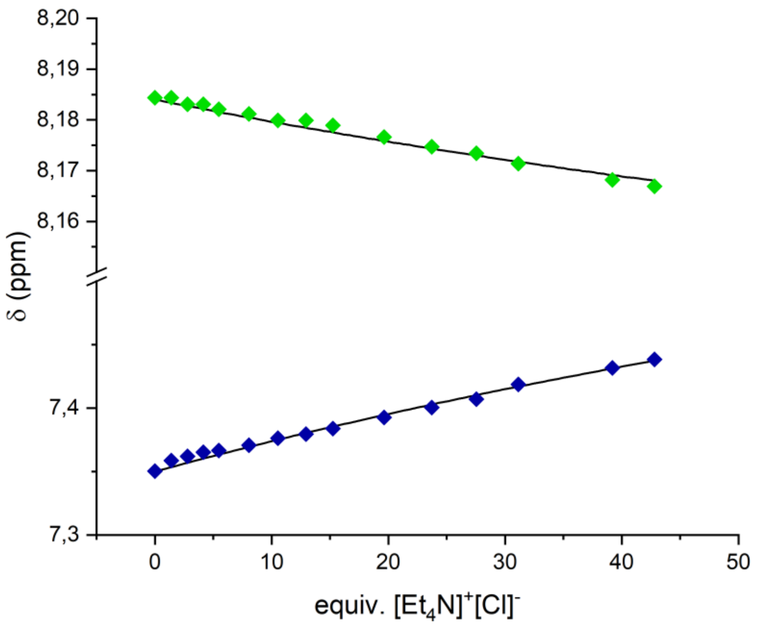

Figure S43. Titration curves for the addition of $[Et_4N]^+[Cl]^-$ to (*E*)-**2**, following the two signals that showed most (and only) significant shifts upon guest addition (indicated in green and blue in Figure S38). Data fits were obtained using HypNMR; $K_a(E)$ = 18.12 ± 1.38 M^{-1}.[3]

X-ray crystallographic data of receptor 2

X-ray crystallographic analysis of (*Z*)-2⊂$[Bu_4N]^+[Cl]^-$

All reflection intensities were measured at 110(2) K using a SuperNova diffractometer (equipped with Atlas detector) with Cu *K*α radiation (λ = 1.54178 Å) under the program CrysAlisPro (Version CrysAlisPro 1.171.39.29c, Rigaku OD, 2017). The same program was used to refine the cell dimensions and for data reduction. The structure was solved with the program SHELXS-2018/3 (Sheldrick, 2018) and was refined on F^2 with SHELXL-2018/3 (Sheldrick, 2018).[6] Analytical numeric absorption correction using a multifaceted crystal model was applied using CrysAlisPro. The temperature of the data collection was controlled using the system Cryojet (manufactured by Oxford Instruments). The H atoms were placed at calculated positions (unless otherwise specified) using the instructions AFIX 23, AFIX 43 or AFIX 137 with isotropic displacement parameters having values 1.2 or 1.5 U_{eq} of the attached C atoms. The H atom attached to N23, N31, N38 and N46 were found from difference Fourier maps, and their coordinates were refined pseudofreely using the DFIX instruction in order to keep the N–H distances within an acceptable range. The structure is ordered. Moreover, two helical enantiomers belonging to the (*Z*)-isomer of the stiff-stilbene [(*P*)-(*Z*) and (*M*)-(*Z*)] are present in the packing lattice with a dihedral angle of 6.6°.[7,8]

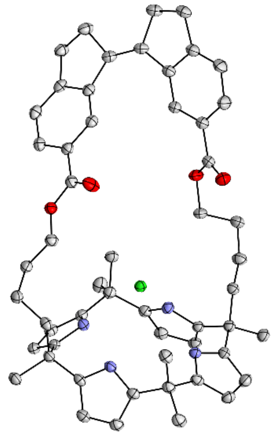

Figure S44. Displacement ellipsoid plot (50% probability level) of (*Z*)-**2**⊂$[Bu_4N]^+[Cl]^-$ at 110 K. H atoms were removed for clarity.

Specified hydrogen bonds (with esds except fixed and riding H)

D-H	H...A	D...A	<(DHA)	
0.90(2)	2.45(2)	3.3029(16)	158(2)	N23-H23N...Cl1
0.88(2)	2.42(2)	3.2758(16)	166(2)	N31-H31N...Cl1
0.91(2)	2.44(2)	3.2971(16)	158(2)	N38-H38N...Cl1
0.87(2)	2.53(2)	3.3875(15)	168(2)	N46-H46N...Cl1

Experimental details (*Z*)-2⊂[$Bu_4N]^+[Cl]^-$

Table S1. Experimental details of (*Z*)-**2**⊂[$Bu_4N]^+[Cl]^-$.

Crystal data	
Chemical formula	$C_{54}H_{60}N_4O_4 \cdot C_{16}H_{36}N \cdot Cl$
M_r	1106.96
Crystal system, space group	Triclinic, *P*-1
Temperature (K)	110
a, *b*, *c* (Å)	10.5224 (4), 15.0974 (6), 21.5264 (7)
α, *β*, *γ* (°)	93.833 (3), 101.716 (3), 108.886 (3)
V ($Å^3$)	3135.6 (2)
Z	2
Radiation type	Cu *K*α
μ (mm^{-1})	0.94
Crystal size (mm)	0.11 × 0.07 × 0.03
Data collection	
Diffractometer	SuperNova, Dual, Cu at zero, Atlas
Absorption correction	Analytical<br>*CrysAlis PRO* 1.171.41.93a (Rigaku Oxford Diffraction, 2020) Analytical numeric absorption correction using a multifaceted crystal model based on expressions derived by R.C. Clark & J.S. Reid. (Clark, R. C. & Reid, J. S. (1995). Acta Cryst. A51, 887-897) Empirical absorption correction using spherical harmonics, implemented in SCALE3 ABSPACK scaling algorithm.
T_{min}, T_{max}	0.936, 0.973
No. of measured, independent and observed [$I > 2\sigma(I)$] reflections	40426, 12243, 8630
R_{int}	0.072
$(\sin \theta/\lambda)_{max}$ ($Å^{-1}$)	0.616
Refinement	
$R[F^2 > 2\sigma(F^2)]$, $wR(F^2)$, *S*	0.046, 0.112, 1.01
No. of reflections	12243
No. of parameters	743
No. of restraints	4
H-atom treatment	H atoms treated by a mixture of independent and constrained refinement
$\Delta\rho_{max}$, $\Delta\rho_{min}$ (e $Å^{-3}$)	0.36, -0.26

Computer programs: *CrysAlis PRO* 1.171.39.29c (Rigaku OD, 2017), *SHELXS2018*/3 (Sheldrick, 2018), *SHELXL2018*/3 (Sheldrick, 2018), *SHELXTL* v6.10 (Sheldrick, 2008).[6]

X-ray crystallographic analysis of (*Z*)-2⊂[$Et_4N]^+[Cl]^-$

All reflection intensities were measured at 110(2) K using a SuperNova diffractometer (equipped with Atlas detector) with Cu *K*α radiation (λ = 1.54178 Å) under the program CrysAlisPro (Version CrysAlisPro 1.171.42.49, Rigaku OD, 2022). The same program was used to refine the cell dimensions and for data reduction. The structure was solved with the program SHELXS-2018/3 (Sheldrick, 2018) and was refined on F^2 with SHELXL-2018/3 (Sheldrick, 2018).[6] Analytical numeric absorption correction using a multifaceted crystal model was applied using CrysAlisPro. The temperature of the data collection was controlled using the system Cryojet (manufactured by Oxford Instruments). The H atoms were placed at calculated positions (unless otherwise specified) using the instructions AFIX 23, AFIX 43 or AFIX 137 with isotropic displacement parameters having values 1.2 or 1.5 U_{eq} of the attached C atoms. The H atoms attached to N23, N31, N38 and N46 were found from difference Fourier maps, and their coordinates were refined pseudofreely using the DFIX instruction in order to keep the N−H bond distances within an acceptable range.

The asymmetric unit contains one molecule of the target compound, one Cl^- anion, two halves of NEt_4^+ cations (both counterions are found at sites of special positions; one cation is found at one site of inversion symmetry while the other cation is found at one site of twofold axial symmetry) and some amount of lattice solvent molecules (see below for further details). The structure is partly disordered. The cation found at one site of inversion symmetry must be disordered. One lattice chloroform lattice solvent molecule is disordered over two orientations, and the occupancy factor of the major component of the disorder refines to 0.538(6). One lattice water molecule (possibly) is found at one site of twofold axial symmetry, and it is most likely disordered. The H atoms from the water molecule could not be retrieved from the difference Fourier map. The crystal that was mounted on the diffractometer was found to be non-merohedrally twinned with two components, and the twin relationship corresponds to a twofold axis along the **a** direction. The BASF scale factor refines to 0.4215(8). Moreover, two helical enantiomers belonging to the (*Z*)-isomer of the stiff-stilbene [(*P)*-(*Z*) and (*M*)-(*Z*)] are present in the packing lattice with a dihedral angle of 9.3°.[7,8]

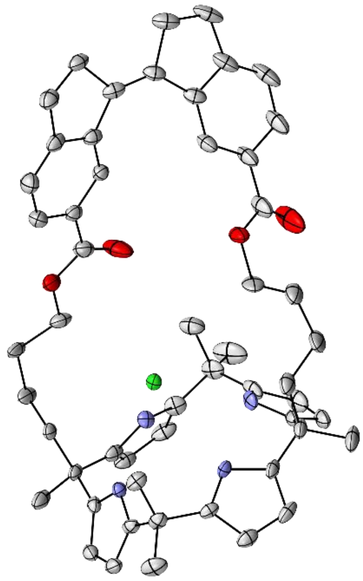

Figure S45. Displacement ellipsoid plot (50% probability level) of (*Z*)-**2**⊂$[Et_4N]^+[Cl]^-$ at 110 K. H atoms were removed for clarity.

Specified hydrogen bonds (with esds except fixed and riding H)

D-H	H...A	D...A	<(DHA)	
0.86(2)	2.44(3)	3.297(3)	175(4)	N23-H23...Cl1
0.85(3)	2.43(3)	3.283(4)	179(4)	N31-H31...Cl1
0.85(3)	2.54(3)	3.388(3)	174(4)	N38-H38...Cl1
0.90(3)	2.47(3)	3.370(4)	178(4)	N46-H46...Cl1

Experimental details (*Z*)-2⊂$[Et_4N]^+[Cl]^-$

Table S2. Experimental details of (*Z*)-**2**⊂$[Et_4N]^+[Cl]^-$.

Crystal data	
Chemical formula	$2(C_{54}H_{60}N_4O_4)\cdot 2(C_8H_{20}N)\cdot 2(CHCl_3)\cdot 2(Cl)\cdot O$
M_r	2244.25
Crystal system, space group	Monoclinic, *C*2/*c*
Temperature (K)	110
a, *b*, *c* (Å)	14.5245 (4), 20.2575 (7), 40.2891 (13)
β (°)	94.925 (3)
V (Å^3)	11810.5 (6)
Z	4
Radiation type	Cu *Kα*
μ (mm^{-1})	2.23
Crystal size (mm)	0.11 × 0.10 × 0.03
Data collection	
Diffractometer	SuperNova, Dual, Cu at zero, Atlas
Absorption correction	Analytical<br>*CrysAlis PRO* 1.171.42.49 (Rigaku Oxford Diffraction, 2022) Analytical numeric absorption correction using a multifaceted crystal model based on expressions derived by R.C. Clark & J.S. Reid. (Clark, R. C. & Reid, J. S. (1995). Acta Cryst. A51, 887-897) Empirical absorption correction using spherical harmonics, implemented in SCALE3 ABSPACK scaling algorithm.
T_{min}, T_{max}	0.828, 0.940
No. of measured, independent and observed [$I > 2\sigma(I)$] reflections	43988, 11279, 5977
R_{int}	0.083
$(\sin \theta/\lambda)_{max}$ (Å^{-1})	0.598
Refinement	
$R[F^2 > 2\sigma(F^2)]$, $wR(F^2)$, *S*	0.049, 0.100, 0.73
No. of reflections	11279
No. of parameters	782
No. of restraints	166
H-atom treatment	H atoms treated by a mixture of independent and constrained refinement
$\Delta\rho_{max}$, $\Delta\rho_{min}$ (e Å^{-3})	0.47, −0.26

Computer programs: *CrysAlis PRO* 1.171.42.49 (Rigaku OD, 2022), *SHELXS2018*/3 (Sheldrick, 2018), *SHELXL2018*/3 (Sheldrick, 2018), *SHELXTL* v6.10 (Sheldrick, 2008).[6]

CD communication experiment between (*S*,*S*)-1 and 2

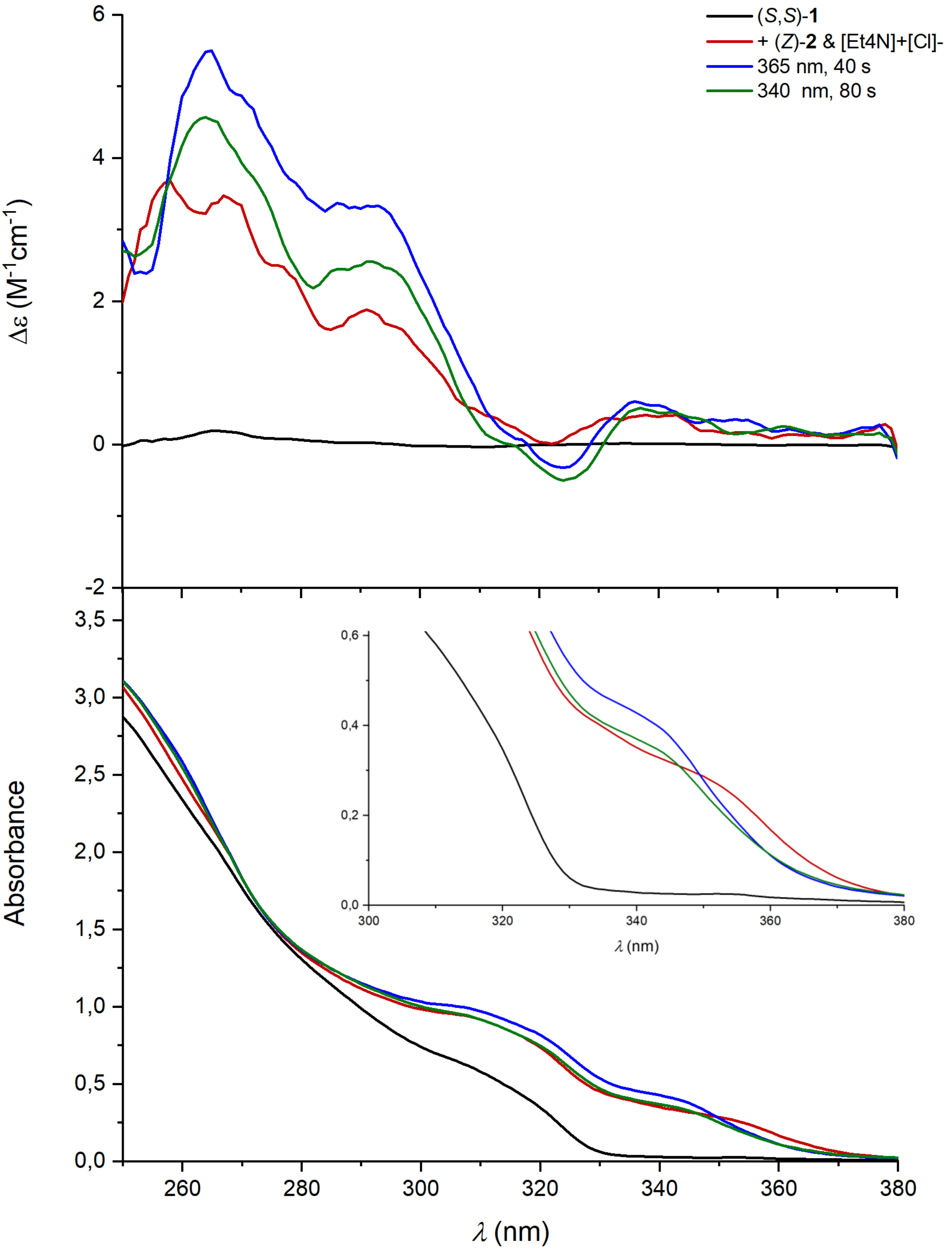

Figure S46. CD spectra and corresponding UV-vis absorbance for the communication process. [(*S*,*S*)-**1**] = [(*Z*)-**2**] = 2.0 × 10^{-4} M, $[[Et_4N]^+[Cl]^-]$ = 1.0 × 10^{-4} M, in dry, argon-degassed in 2.5vol% $CHCl_3/CH_2Cl_2$. T = 22 °C, sensitivity = 5 mdeg, 20 nm/min, range 250-380 nm.

^{1}H NMR communication experiment between (*S*,*S*)-1 and 2

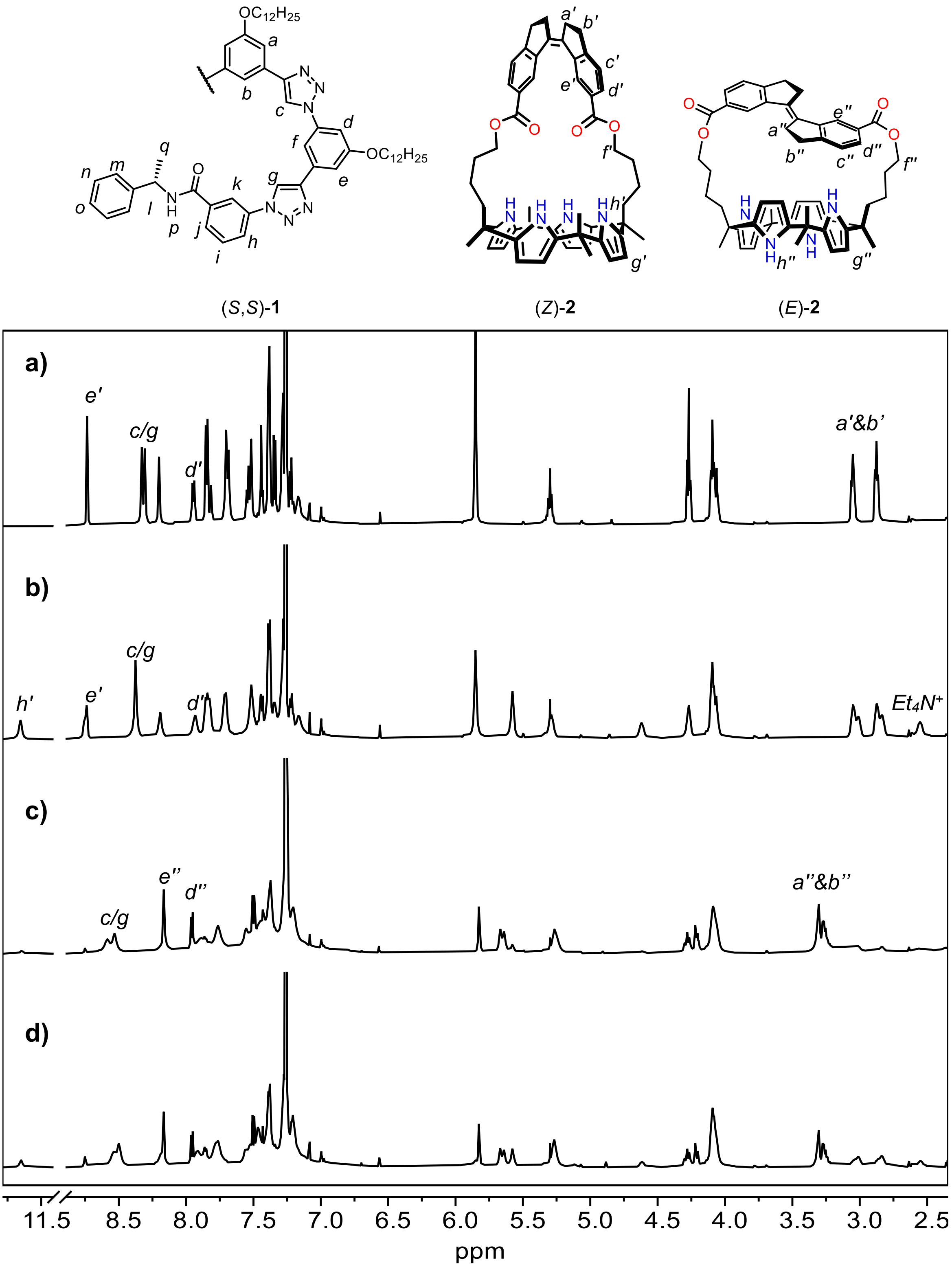

Figure S47. ^{1}H NMR (600 MHz) spectra of the communication process where (x) are signals of (*S*,*S*)-**1**, (x') are signals for (*Z*)-**2** and (x'') are signals for (*E*)-**2**. a) (*S*,*S*)-**1** and (*Z*)-**2** (1.05 mM in dry, Ar-degassed $CDCl_3$); b) addition of $[Et_4N]^+[Cl]^-$ in $CDCl_3$ (0.53 mM), 3% Cl^- bound to **1**; c) irradiation for 30 s with 365 nm, (*E*)/(*Z*) 90:10, 15% Cl^- bound to **1**; d) irradiation for 70 s with 340 nm, (*E*)/(*Z*) 70:30, 12% Cl^- bound to **1**. *E*/*Z*-ratios were determined through relative integration of H_a, H_b and H_d ^{1}H NMR signals of the isomers of **2**.

CD control experiment of isomerization of 2 in presence of $[Et_4N]^+[Cl]^-$

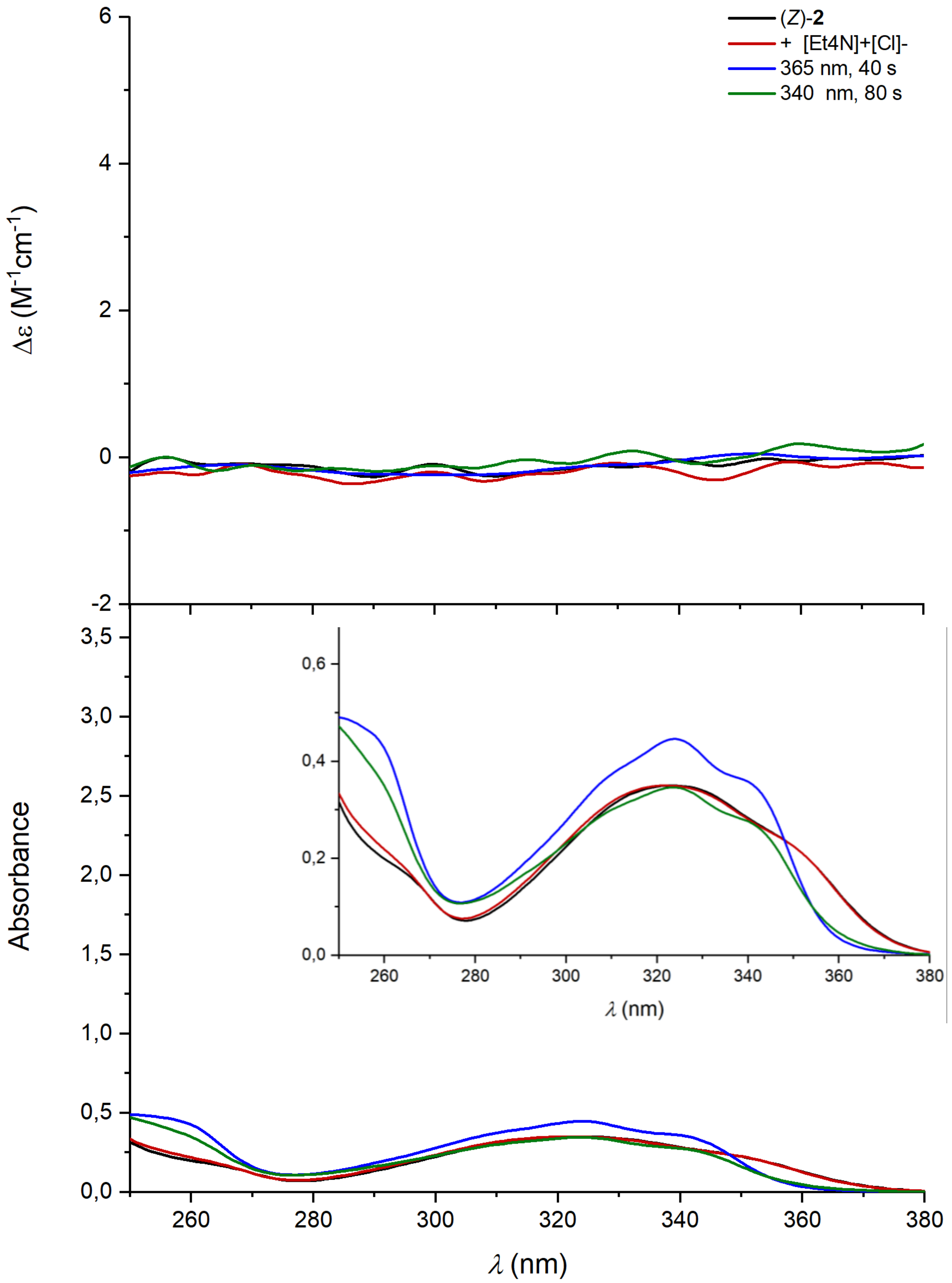

Figure S48. CD spectra and corresponding UV-vis absorbance of (*Z*)-**2** under the conditions of the communication experiment, in absence of foldamer **1**. [(*Z*)-**2**] = 2.0×10^{-4} M, $[[Et_4N]^+[Cl]^-]$ = 1.0×10^{-4} M, in dry, argon-degassed CH_2Cl_2. T = 22 °C, sensitivity = 5 mdeg, 20 nm/min, range 250-380 nm.

^{1}H NMR control experiment of isomerization of 2 in presence of $[Et_4N]^+[Cl]^-$

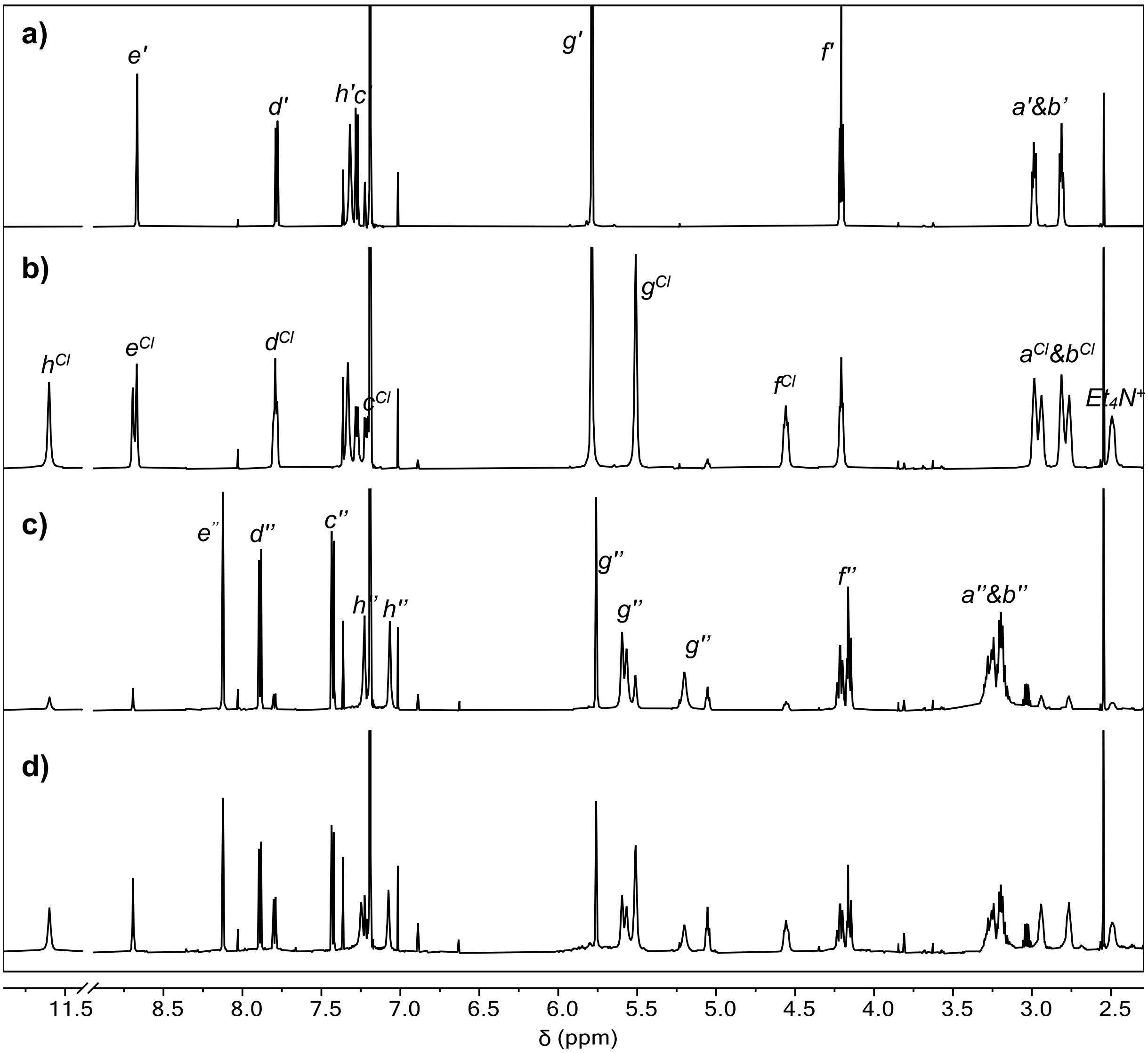

Figure S49. ^{1}H NMR (600 MHz) spectra of (*Z*)-**2** under the conditions of the communication experiment, in absence of foldamer **1**; (x') are signals for (*Z*)-**2**, (x^{Cl}) are signals for (*Z*)-**2**$\subset Cl^-$ and (x'') are signals for (*E*)-**2**. a) (*Z*)-**2** (1.0 mM in dry, Ar-degassed $CDCl_3$); b) addition of $[Et_4N]^+[Cl]^-$ in $CDCl_3$ (0.50 mM); c) irradiation for 75 s with 365 nm, (*E*)/(*Z*) 90:10; d) irradiation for 145 s with 340 nm, (*E*)/(*Z*) 67:33. *E*/*Z*-ratios were determined through relative integration of all ^{1}H NMR signals of the isomers of **2**.

Speciation analysis of ^{1}H NMR communication experiment

A speciation analysis was performed using Hyss software.[10] The concentrations chosen were similar as in the ^{1}H NMR communication experiment, i.e. (*S*,*S*)-**1** and (Z)-**2** were present at 1.0 mM and $[Et_4N]^+[Cl]^-$ at 0.5 mM. The binding constants for chloride, as determined in $CDCl_3$ were used, i.e. K_a(**1**, $CDCl_3$) = 1.31 × 10^3 M^{-1}; K_a((*Z*)-**2**, $CDCl_3$) = 2.80 × 10^4 M^{-1}; K_a((*E*)-**2**, $CDCl_3$) = 18 M^{-1}. The amount of formed complex for each of the species was then determined at the *E*/*Z* ratios of **2** present during the communication experiment, i.e. *E*/*Z* (start) = 0:100, *E*/*Z* (PSS_{365}) = 90:10 and *E*/*Z* ($PSS3_{40}$) = 70:30 (see Figure 6b and S46, as determined by ^{1}H NMR signal integration).

Table S3. Speciation analysis of the ^{1}H NMR communication experiment.

E/*Z* ratio of **2**	free **1** (mM)	free (*Z*)-**2** (mM)	free (*E*)-**2** (mM)	free Cl^- (mM)	**1**⊂Cl^- (mM)	(*Z*)-**2**⊂Cl^- (mM)	(*E*)-**2**⊂Cl^- (mM)	**1**⊂Cl^- (%)	**2**⊂Cl^- (%)
0:100	0.96	0.56	0.00	0.03	0.04	0.44	0.00	4	44
90:10	0.79	0.02	0.90	0.20	0.21	0.09	0.003	21	9
70:30	0.86	0.07	0.70	0.12	0.14	0.23	0.002	14	24

DFT calculations of foldamer 1

Geometry optimization of foldamer 1

Calculations were performed using Gaussian 09.[9] Geometry optimizations were performed at the B3LYP/6-31+G(d,p) level of theory using and IEFPCM CH_2Cl_2 solvation model. $[Me_4N]^+$ was used as countercation and the OC_{12} side chains were omitted to reduce computational costs. All computed stationary points were confirmed as local minima by frequency analysis.

First, the helically folded structure of *P*-(*S*,*S*)-**1**$\subset Cl^-$ [with its enantiomer *M*-(*R*,*R*)-**1**$\subset Cl^-$] was optimized. Next, this structure was used to generate the input structure of the less favored diastereomer by interchanging the methyl group and hydrogen atom at the stereogenic centers, giving *P*-(*R*,*R*)-**1**$\subset Cl^-$ (with its enantiomer *M*-(*S*,*S*)-**1**). Related input structures, with different dihedral angles between the amide group and the chiral methyl substituent all converged towards the same geometry. By comparing the energy of the two helical isomers of one enantiomer of the chiral amide, it was determined which helicity dominates. Through TD-DFT, the computational ECD was then compared with experimental data.

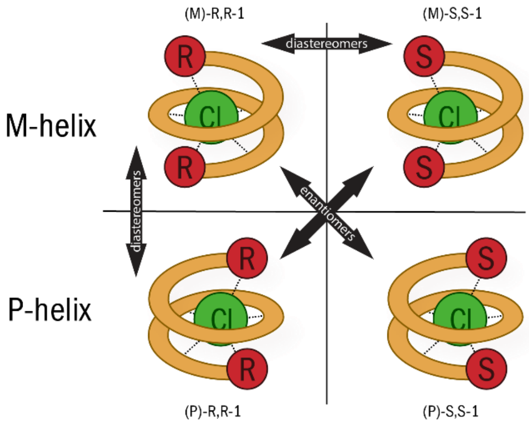

Scheme S1. Overview of enantiomeric and diastereomeric relations in **1**.

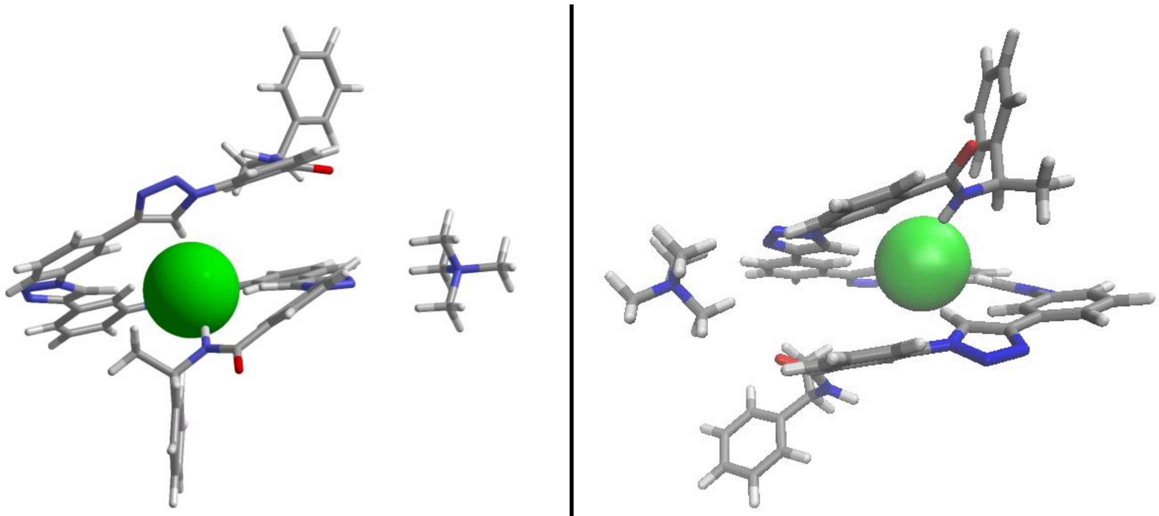

P-(*S*,*S*)-**1** (=*M*-(*R*,*R*)-**1**)
ΔG = -9849019.72 kJ/mol

P-(*R*,*R*)-**1** (=*M*-(*S*,*S*)-**1**)
ΔG = -9848990.61 kJ/mol

P-(*S*,*S*)-**1** vs *P*-(*R*,*R*)-**1** **=** *P*-(*S*,*S*)-**1** vs *M*-(*S*,*S*)-**1**: ΔΔG = -29.1 kJ/mol, $K = 1.542 \times 10^5$.

Figure S50. DFT-optimized structures of *P*-(*S*,*S*)-**1**⊂Cl^- and *P*-(*R*,*R*)-**1**⊂Cl^- with respective thermodynamic parameters.

Table S4 Cartesian coordinates of (*P*)-*S*,*S*-**1**.

Atom	X	Y	Z
Cl	1.63450	-0.06670	0.20840
H	3.82880	0.73210	4.73380
C	-1.36800	3.05790	1.71650
C	-1.18090	4.44690	1.70530
C	-2.28120	5.28230	1.96110
C	-3.53390	4.72150	2.22040
C	-3.71220	3.33630	2.23190
C	-2.62070	2.48830	1.97870
C	0.15470	4.99150	1.42670
C	1.33790	4.30880	1.20680
N	2.26330	5.27490	0.98270
N	1.69320	6.50450	1.06180
N	0.42710	6.33140	1.33150
C	3.64090	5.10290	0.65540
C	4.02340	3.94460	-0.02090
C	5.37140	3.72640	-0.33850
C	6.31800	4.70620	0.00480
C	5.91610	5.87020	0.66270
C	4.57810	6.07920	1.00460
C	5.75990	2.49590	-1.03930
C	5.05320	1.31530	-1.19830
N	5.86610	0.52720	-1.94900
N	7.02290	1.17800	-2.23980
N	6.95860	2.35760	-1.68860
C	5.61730	-0.78450	-2.45360
C	4.29860	-1.20410	-2.63250
C	4.03420	-2.48770	-3.13270
C	5.10990	-3.32630	-3.46920
C	6.42290	-2.88760	-3.29310
C	6.69210	-1.61880	-2.77740
C	-0.91700	-3.14970	-3.10400
C	-1.51820	-1.91790	-2.83270
C	-2.90710	-1.84930	-2.65920
C	-3.67610	-3.01670	-2.76300
C	-3.06460	-4.23700	-3.04990
C	-1.68150	-4.31500	-3.21610
C	-3.62550	-0.55960	-2.35500
O	-4.68060	-0.55870	-1.70630
N	-3.06110	0.57880	-2.83060
C	-3.62590	1.91140	-2.59380
C	-4.79320	2.24020	-3.52340
C	-4.76470	1.91280	-4.88620
C	-5.82060	2.27310	-5.72850
C	-6.92110	2.97010	-5.21930
C	-6.95850	3.30080	-3.86150
C	-5.90170	2.93560	-3.02200
H	-4.00640	1.90850	-1.56850
H	-0.52330	2.40570	1.52180
H	-2.15090	6.35900	1.95670
H	-4.68890	2.91020	2.43390
H	1.58490	3.25940	1.21090
H	3.27340	3.22320	-0.32350
H	7.36210	4.54980	-0.24260
H	4.26470	6.97260	1.53120
H	4.09690	0.97850	-0.82030
H	3.47760	-0.53680	-2.39660
H	4.91130	-4.31760	-3.86110
H	7.70910	-1.27800	-2.62790
H	-0.90030	-1.03120	-2.74360
H	-4.74910	-2.95320	-2.62130
H	-3.66410	-5.13750	-3.13680
H	-1.19500	-5.26030	-3.42370
H	-2.25480	0.50910	-3.43560
C	-2.50210	2.95320	-2.70520
H	-3.91910	1.36690	-5.29550
H	-5.78330	2.00860	-6.78160
H	-7.74230	3.24860	-5.87340
H	-7.81120	3.83650	-3.45370
H	-1.70410	2.73810	-1.98810
H	-2.89670	3.95100	-2.49710
H	-2.07660	2.96590	-3.71510
H	-5.94120	3.18850	-1.96500
H	7.24880	-3.54410	-3.54830
H	6.65530	6.61970	0.92730
N	2.31280	-3.96080	-4.16160
C	2.64650	-2.93200	-3.31930
C	1.47780	-2.45350	-2.74960
H	1.29900	-1.69910	-1.99360
N	0.49510	-3.22180	-3.29010
N	1.02230	-4.13470	-4.14980
H	-4.38110	5.37220	2.41620
H	5.04230	-4.06990	4.43310
H	5.63660	-2.77330	6.45890
O	1.84300	-5.23170	2.63130
C	4.77930	-3.01670	4.49530
C	4.61910	-2.77780	0.96630
C	5.11490	-2.28520	5.64040
C	1.48450	-4.14740	2.14350
C	3.77660	-3.21560	2.17280
C	0.02550	-3.89290	1.86410
C	4.10440	-2.41000	3.42790
C	-0.80310	-4.98220	1.55920
N	2.34590	-3.14600	1.84270
H	-0.36860	-5.97380	1.49660

C	-0.53040	-2.61480	1.97790	N	-3.88730	0.37610	2.41540
H	0.08690	-1.76860	2.25450	H	4.41200	-3.41790	0.10340
C	4.77420	-0.93320	5.73150	H	3.97890	-4.26740	2.38500
C	-2.16720	-4.78650	1.34100	H	-7.37170	0.63910	4.49970
C	-1.89870	-2.43420	1.76210	C	-7.23500	0.24990	3.49070
H	-0.81970	0.11410	1.24670	H	-6.21830	0.43980	3.14320
H	3.24040	-0.56180	2.71110	H	-7.96170	0.70070	2.81470
C	-1.82240	0.06080	1.65320	N	-7.46550	-1.24120	3.51640
H	4.40080	-1.74270	0.68840	C	-6.47060	-1.88530	4.44880
C	-2.72670	-3.51020	1.43520	H	-5.46690	-1.67210	4.08160
H	5.03100	-0.36280	6.61960	H	-6.61080	-1.47340	5.44780
H	5.68350	-2.85140	1.20860	H	-6.64920	-2.96050	4.45780
H	-2.80160	-5.62990	1.08730	C	-7.27450	-1.79950	2.12870
N	-2.44320	-1.12190	1.89020	H	-6.25200	-1.59490	1.81310
C	-2.75580	1.02520	1.99870	H	-7.98430	-1.31810	1.45660
H	2.01390	-2.31040	1.35990	H	-7.45440	-2.87410	2.15880
C	3.76680	-1.05040	3.52790	C	-8.86460	-1.52850	3.99120
N	-3.70040	-0.91610	2.35480	H	-8.98730	-1.11450	4.99200
H	-3.78250	-3.34690	1.25550	H	-9.57060	-1.06350	3.30340
C	4.09770	-0.31880	4.67110	H	-9.01610	-2.60750	4.01120

Sum of electronic and zero-point Energies = -3751.169293
Sum of electronic and thermal Energies = -3751.098996
Sum of electronic and thermal Enthalpies = -3751.098052
Sum of electronic and thermal Free Energies = -3751.292982

Table S5 Cartesian coordinates of (*P*)-*R*,*R*-**1**.

Atom	X	Y	Z
Cl	-1.67740	0.30900	-0.14400
H	-5.85090	0.55750	-4.83860
C	1.37420	3.06290	-1.95360
C	1.31770	4.46030	-1.87310
C	2.42620	5.21000	-2.30140
C	3.55520	4.55620	-2.80040
C	3.60030	3.16250	-2.88570
C	2.49990	2.40010	-2.45940
C	0.10060	5.09930	-1.35500
C	-1.09490	4.50670	-0.98780
N	-1.88310	5.53300	-0.58070
N	-1.21890	6.71230	-0.68830
N	-0.02820	6.44920	-1.15610
C	-3.21360	5.46340	-0.07090
C	-3.62530	4.28950	0.56000
C	-4.93420	4.17440	1.04990
C	-5.80720	5.26750	0.92220
C	-5.37380	6.44290	0.30570
C	-4.07880	6.55320	-0.20550
C	-5.35450	2.92810	1.70300
C	-4.74360	1.68530	1.69230
N	-5.53270	0.90820	2.47860
N	-6.58620	1.62490	2.95160
N	-6.47930	2.83590	2.48060
C	-5.33830	-0.45240	2.86330
C	-4.04850	-0.98410	2.83850
C	-3.83140	-2.31610	3.22150
C	-4.92290	-3.09190	3.64650
C	-6.20540	-2.54210	3.67350
C	-6.42990	-1.22260	3.27710
C	1.02280	-3.27890	2.55780
C	1.69810	-2.05900	2.46510
C	3.06050	-2.04620	2.13900
C	3.72980	-3.25690	1.91310
C	3.04820	-4.46840	2.03110
C	1.68920	-4.48940	2.34790
C	3.84680	-0.76480	2.01350
O	4.76180	-0.66100	1.18590
N	3.49760	0.22940	2.86750
C	4.10360	1.57550	2.90220
C	5.58700	1.52530	3.28840
C	5.98860	0.72650	4.37170
C	7.31950	0.69690	4.79150
C	8.28080	1.47490	4.13640
C	7.89290	2.27770	3.06190
C	6.55700	2.30280	2.64280
C	3.77830	2.36460	1.62800
H	0.52360	2.47850	-1.62030
H	2.39650	6.29290	-2.24710
H	4.47900	2.66590	-3.28290
H	-1.43430	3.48290	-1.00150
H	-2.92630	3.47140	0.69000
H	-6.81950	5.19000	1.30300
H	-3.74440	7.45680	-0.70050
H	-3.86820	1.30570	1.18080
H	-3.21180	-0.36580	2.53410
H	-4.76120	-4.12060	3.94860
H	-7.42490	-0.79500	3.28750
H	1.15730	-1.13360	2.63110
H	4.78290	-3.23680	1.65510
H	3.57250	-5.40430	1.86650
H	1.14640	-5.42400	2.42380
H	2.79820	0.02690	3.56680
H	5.25300	0.11620	4.88860
H	7.60530	0.06980	5.63140
H	9.31670	1.45620	4.46220
H	8.62750	2.88870	2.54470
H	6.28360	2.93320	1.80390
H	-7.04460	-3.15000	3.99670
H	-6.05660	7.28100	0.20780
N	-2.13550	-4.02680	3.85470
C	-2.47220	-2.87270	3.19590
C	-1.33300	-2.39590	2.56800
H	-1.16930	-1.55900	1.90180
N	-0.36390	-3.29190	2.89080
N	-0.87030	-4.27990	3.67670
H	4.40830	5.14070	-3.13230
H	-5.25630	-4.16950	-3.44420
H	-6.22770	-3.65150	-5.65740
O	-2.91450	-4.69590	-2.45100
C	-5.39250	-3.13590	-3.74110
C	-5.93520	-2.84100	-4.99540
C	-2.31870	-3.68320	-2.05180
C	-4.40450	-2.37850	-1.48890
C	-0.80990	-3.62630	-2.10540
C	-5.00520	-2.10640	-2.86930
C	-0.08930	-4.82740	-2.04480
N	-2.94030	-2.58890	-1.55470
H	-0.63010	-5.76140	-1.94030
C	-0.12140	-2.42030	-2.26910
H	-0.66400	-1.48780	-2.36390
C	-6.10200	-1.51260	-5.40000
C	1.30430	-4.81390	-2.11750
C	1.27410	-2.41980	-2.33450
H	0.56280	0.20110	-1.59970

H	-4.88810	0.03260	-2.62150
C	1.49580	0.05750	-2.12990
C	1.99980	-3.61060	-2.25550
H	-6.52630	-1.28610	-6.37420
H	1.85830	-5.74530	-2.05640
N	1.94790	-1.17110	-2.48480
C	2.48300	0.93350	-2.54940
H	-2.38400	-1.82010	-1.18050
C	-5.17920	-0.77750	-3.28580
N	3.15880	-1.07790	-3.08770
H	3.08200	-3.59190	-2.29910
C	-5.72230	-0.47890	-4.53910
N	3.48100	0.18970	-3.12150
C	-5.12890	-3.46920	-0.68650
H	7.35830	-0.16360	-4.64590
C	6.98850	0.09670	-3.65410
H	5.91170	0.27070	-3.67810
H	7.50900	0.97850	-3.28040
N	7.26280	-1.05480	-2.71900
C	6.56260	-2.28720	-3.23320
H	5.49210	-2.08550	-3.27740
H	6.94800	-2.51800	-4.22630
H	6.76530	-3.11380	-2.55220
C	6.73630	-0.71680	-1.34610
H	5.66480	-0.54150	-1.41340
H	7.24430	0.17860	-0.98920
H	6.93540	-1.55200	-0.67650
C	8.74420	-1.30820	-2.64320
H	9.11250	-1.54670	-3.64080
H	9.23500	-0.41090	-2.26680
H	8.92450	-2.14350	-1.96660
H	2.69960	2.34030	1.45280
H	4.07540	3.41130	1.73820
H	4.28080	1.94440	0.75440
H	3.58340	2.07150	3.72970
H	-5.06710	-4.44380	-1.17140
H	-4.68680	-3.55040	0.31100
H	-6.18300	-3.19690	-0.57340
H	-4.49450	-1.44680	-0.92360

Sum of electronic and zero-point Energies = -3751.159632
Sum of electronic and thermal Energies = -3751.089485
Sum of electronic and thermal Enthalpies = -3751.088541
Sum of electronic and thermal Free Energies = -3751.281892

Time-dependent DFT calculations for *P*-(*S*,*S*)-1 vs *P*-(*R*,*R*)-1

Using the DFT geometry-optimized structures, TD-DFT calculations (solving for 30 singlet excited states) were performed at the same B3LYP/6-311++G(d,p) level of theory and IEFPCM dichloromethane solvation model using Gaussian 09.[9]

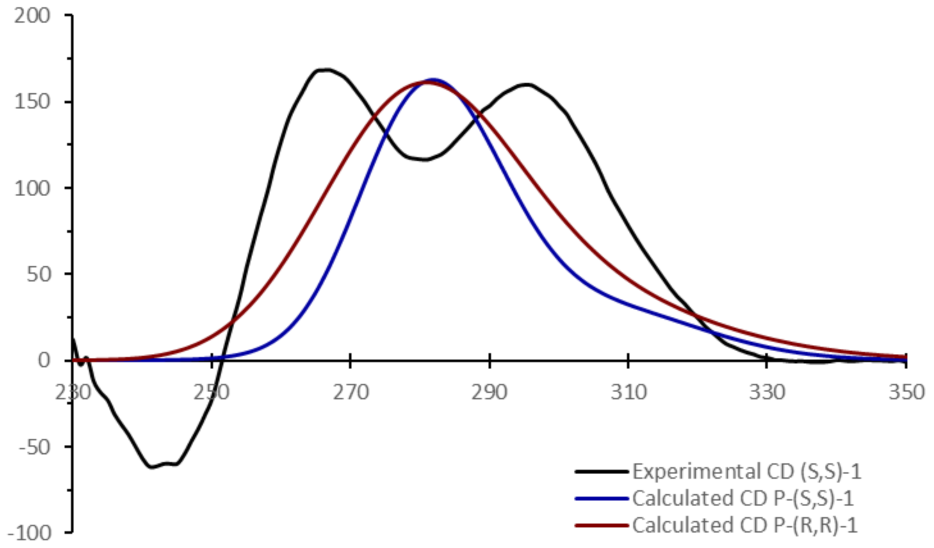

Figure S51. Experimental CD of (*S*,*S*)-**1** and computed ECD spectra plotted with peak half-width at half height set to 0.25 eV of *P*-(*S*,*S*)-**1** and *P*-(*R*,*R*)-**1**. The calculated positive CD signal for the *P*-helix corresponds with the experimentally found spectrum for (*S*,*S*)-**1**.

References

(1) D. Villarón, M. A. Siegler and S. J. Wezenberg, *Chem. Sci.* **2021**, *12*, 3188–3193.

(2) V. R. Naidu, M. C. Kim, J. Suk, H.-J. Kim, M. Lee, E. Sim and K.-S. Jeong, *Org. Lett.*, **2008**, *10*, 5373–5376.

(3) C. Frassineti, S. Ghelli, P. Gans, A. Sabatini, M. S. Moruzzi and A. Vacca, *Anal. Biochem.* **1995**, *231*, 374–382.

(4) P. Thordarson, *Chem. Soc. Rev.* **2011**, *40*, 1305–1323.

(5) K. Hirose, In *Analytical Methods in Supramolecular Chemistry*, Wiley, 2012; pp 27–66.

(6) G. M. Sheldrick, *Acta Crystallogr. Sect. C Struct. Chem*. **2015**, *71*, 3–8.

(7) D. H. Waldeck, *Chem. Rev*. **1991**, *91*, 415–436.

(8) D. Villarón and S. J. Wezenberg, *Angew. Chem. Int. Ed*. **2020**, *59*, 13192–13202.

(9) M. J. Frisch, G. W. Trucks, H. B. Schlegel, G. E. Scuseria, M. A. Robb, J. R. Cheeseman, G. Scalmani, V. Barone, G. A. Petersson, H. Nakatsuji, X. Li, M. Caricato, A. Marenich, J. Bloino, B. Janesko, G. Gomperts, R. B. Mennucci, H. P. Hratchian, J. V. Ort, J. W. Ochterski, R. L. Martin, K. Morokuma, O. Farkas, J. B. Foresman and D. J. Fox, Gaussian 09, Revision A.02. Gaussian, Inc.: Wallingford CT 2016.

(10) L. Alderighi, P. Gans, A. Ienco, D. Peters, A. Sabatini, and A. Vacca, *Coord. Chem. Rev.* **1999**, *184*, 311–318.
